# Marine-Inspired Bis-indoles Possessing Antiproliferative Activity against Breast Cancer; Design, Synthesis, and Biological Evaluation

**DOI:** 10.3390/md18040190

**Published:** 2020-04-02

**Authors:** Wagdy M. Eldehna, Ghada S. Hassan, Sara T. Al-Rashood, Hamad M. Alkahtani, Abdulrahman A. Almehizia, Ghada H. Al-Ansary

**Affiliations:** 1 Department of Pharmaceutical Chemistry, Faculty of Pharmacy, Kafrelsheikh University, Kafr El-Sheikh 33516, Egypt; wagdy2000@gmail.com; 2 Department of Medicinal Chemistry, Faculty of Pharmacy, Mansoura University, Mansoura 35516, Egypt; Ghadak25@yahoo.com; 3 Department of Pharmaceutical Chemistry, College of Pharmacy, King Saud University, P.O. Box 2457, Riyadh 11451, Saudi Arabia; haalkahtani@ksu.edu.sa (H.M.A.); mehizia@ksu.edu.sa (A.A.A.); 4 Department of Pharmaceutical Chemistry, Faculty of Pharmacy, Ain Shams University, Cairo P.O. Box 11566, Egypt; ghada.mohamed@pharma.asu.edu.eg; 5 Department of Pharmaceutical Chemistry, Pharmacy Program, Batterejee Medical College, Jeddah P.O. Box 6231, Saudi Arabia

**Keywords:** marine-inspired, breast cancer, bis-indoles, synthesis, apoptosis

## Abstract

Diverse indoles and bis-indoles extracted from marine sources have been identified as promising anticancer leads. Herein, we designed and synthesized novel bis-indole series **7a**–**f** and **9a**–**h** as Topsentin and Nortopsentin analogs. Our design is based on replacing the heterocyclic spacer in the natural leads by a more flexible hydrazide linker while sparing the two peripheral indole rings. All the synthesized bis-indoles were examined for their antiproliferative action against human breast cancer (MCF-7 and MDA-MB-231) cell lines. The most potent congeners **7e** and **9a** against MCF-7 cells (IC_50_ = 0.44 ± 0.01 and 1.28 ± 0.04 μM, respectively) induced apoptosis in MCF-7 cells (23.7-, and 16.8-fold increase in the total apoptosis percentage) as evident by the externalization of plasma membrane phosphatidylserine detected by Annexin V-FITC/PI assay. This evidence was supported by the Bax/Bcl-2 ratio augmentation (18.65- and 11.1-fold compared to control) with a concomitant increase in the level of caspase-3 (11.7- and 9.5-fold) and p53 (15.4- and 11.75-fold). Both compounds arrested the cell cycle mainly in the G2/M phase. Furthermore, **7e** and **9a** displayed good selectivity toward tumor cells (*S.I*. = 38.7 and 18.3), upon testing of their cytotoxicity toward non-tumorigenic breast MCF-10A cells. Finally, compounds **7a**, **7b**, **7d**, **7e**, and **9a** were examined for their plausible CDK2 inhibitory action. The obtained results (% inhibition range: 16%–58%) unveiled incompetence of the target bis-indoles to inhibit CDK2 significantly. Collectively, these results suggested that herein reported bis-indoles are good lead compounds for further optimization and development as potential efficient anti-breast cancer drugs.

## 1. Introduction

Drug discovery from marine sources is a prehistoric praxis. Recently, the identification and development of novel molecules based on natural heterocyclic scaffold have been an area of growing focus. Surveying the literature reveals that the anticancer activity of diverse bis-indole compounds extracted from marine sources including plants, fungi, algae, and marine mollusks was broadly discussed in diverse manuscripts [1,2]. 

**Figure 1:**
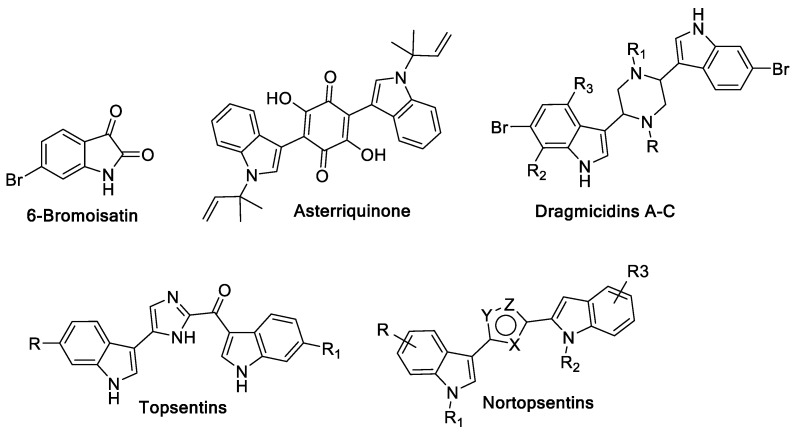
Indole and bis-indole marine products that have reported anticancer activity.

Diverse substituted indole and bis-indole derivatives extracted from marine sources have been shown to exhibit significant antiproliferative activity [3,4]. Recently, Edwards et al. [5] conducted a study on purified 6-bromoisatin (Figure 1) extracted from the Australian marine mollusk *Dicathais orbita* known for its antineoplastic activity. The study revealed that 6-bromoisatin markedly reduced the proliferation and concomitantly induced apoptosis in human colon cancer cell lines HT29 and Caco2 cells [5]. A latter study was conducted on the same isatin derivative by Esmaeelian et al. [6] which supported the efficacy of 6-bromoisatin at a concentration of 0.05 mg/g to induce apoptosis in colorectal cancer cells leading ultimately to inhibition of cancer proliferation [6].

Moreover, many bis-indole derivatives isolated from marine sources manifest anticancer activity. Asterriquinone (Figure 1), isolated from Aspergillus fungi, possesses symmetrical bis-indole moieties separated by quinone spacer and showed in vivo activity against Ehrlich carcinoma, ascites hepatoma AH13, and mouse P388 leukemia [7]. In addition, Dragmacidins A–C, isolated from a large number of deepwater sponges showed modest cytotoxic activity, Figure 1 [8,9,10]. Topsentins (Figure 1), extracted from the Mediterranean sponge *Topsentia genitrix*, exhibited antitumor and antiviral activities [11,12]. Nortopsentins A–C (Figure 1), that feature imidazole ring spacer, were isolated from *Spongosorites ruetzleri* and showed in vitro cytotoxicity against P388 cells [13,14].

As the reservoir of living organisms is inevitably limited, it became an urgent necessity for medicinal chemists to synthesize biologically active natural molecules and their derivatives to meet the expanding need of such medicinal agents.

**Figure 2:**
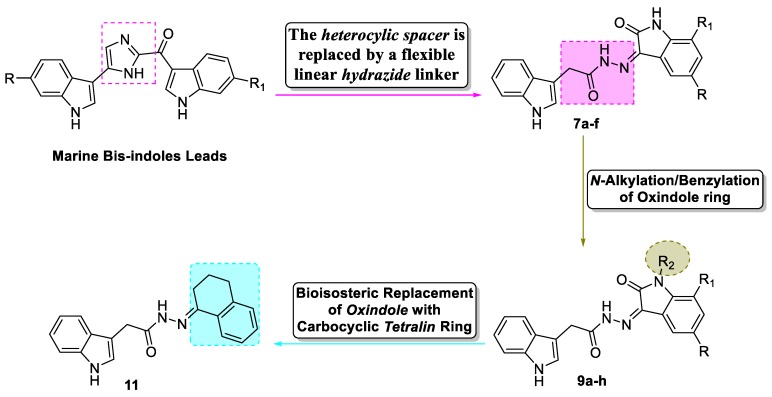
Structure-based design of target bis-indole derivatives (**7a**–**f** and **9a**–**h**), and **11**.

Inspired by the aforementioned discoveries and in connection with our research work concerning the development of effective anti-breast cancer agents [15,16,17,18], we were endeavored to design novel indole derivatives that promisingly possess antiproliferative activity. Perceiving the significance of these facts and based on our growing research interest in marine natural products, we were persuaded to tackle this study to design and synthesize marine-inspired bis-indole derivatives that have potential in vitro antitumor activity against breast cancer. Herein, we designed and synthesized three novel bis-indole sets **7a**–**f**, **9a**–**h**, and **11** as Topsentin and Nortopsentin analogs. Our design was based on replacing the rigid heterocyclic spacer in the natural products by a more flexible hydrazide linker while sparing the two peripheral indole rings to furnish the first set of target compounds **7a**–**f** (Figure 2). Thereafter, the oxindole moiety was decorated with different *N*-alkyl (allyl, n-propyl, iso-butyl; compounds **9a**–**c**) and *N*-benzyl (compounds **9d**–**h**) substituents to fulfill further elaboration for the target bis-indoles and to probe a worthy structure-activity relationship (SAR). Furthermore, a bioisosteric replacement approach was adopted to replace the oxindole ring with carbocyclic tetralin ring (compound **11**), to explore the significance of the bis-indole scaffold, Figure 2.

In this study, all of the synthesized compounds **7**, **9** and **11** were evaluated for their antiproliferative activity against MCF-7 cells and MDA-MB-231 cancer cell lines. Three of the most potent compounds induced apoptosis in MCF-7 cells as evidenced by the externalization of plasma membrane phosphatidylserine detected by Annexin V-FITC/PI dual staining assay. This evidence was supported by the Bax/Bcl-2 ratio augmentation with a concomitant increase in the level of caspase-3 and p53. Moreover, scrutinizing the results of cell cycle analysis unraveled that these compounds arrest the cell cycle in the G0/G1 phase.

## 2. Results

### 2.1. Chemistry

**Scheme 1:**
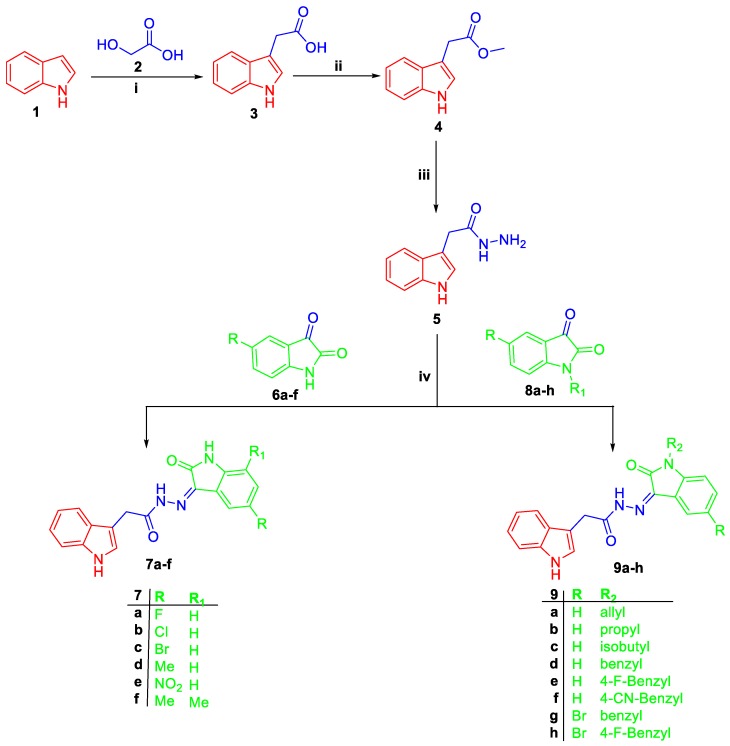
Synthesis of target bis-indole derivatives **7a**–**f** and **9a**–**h**; Reagents and conditions: (i) (a) KOH / heating at 250 °C 18 h, (b) H_2_O, cooling to 10 °C, HCl; (ii) MeOH/H_2_SO_4_ (catalytic)/reflux 8 h; (iii) 99% NH_2_NH_2_.H_2_O/EtOH/reflux 3 h; (iv) EtOH/AcOH (catalytic)/reflux 2 h.

**Scheme 2:**
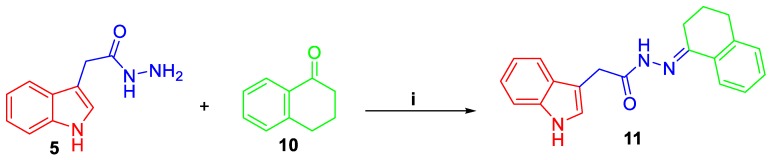
Synthesis of target compound **11**; Reagents and conditions: (i) EtOH/AcOH (catalytic)/reflux 2 h.

The synthetic pathways proposed to obtain the target bis-indole derivatives (**7a**–**f** and **9a**–**h**), and **11** were depicted in Scheme 1 and Scheme 2. In Scheme 1, the Fischer esterification procedure was applied to 3-indoleacetic acid 3 [19] to afford methyl 1*H*-indole-2-carboxylate **4**, which subsequently undergone hydrazinolysis through reaction with 99% hydrazine hydrate in ethyl alcohol under reflux temperature to furnish key intermediate 1*H*-indole-2-carbohydrazide **5** in 82% yield. 

Thereafter, 1*H*-indole-2-carbohydrazide **5** was condensed with different *N*-unsubstituted 1*H*-indole-2,3-diones **6a**–**f**, *N*-substituted 1*H*-indole-2,3-diones **8a**–**h**, or 1-tetralone **10** in absolute ethyl alcohol with catalytic drops of acetic acid to produce target indole derivatives **7a**–**f**, **9a**–**h**, and **11**, respectively (Scheme 1 and Scheme 2).

Postulated structures of the herein reported indole derivatives **7a**–**f**, **9a**–**h**, and **11** are in full agreement with the spectral and elemental analyses data.

### 2.2. Biological Evaluation

#### 2.2.1. Antiproliferative Activity against Breast Cancer MCF-7 and MDA-MB-231

The biological evaluation journey started by exploring the antiproliferative activity of the pursed indole derivatives (**7a**–**f**, **9a**–**h**, and **11**) against breast cancer cell line; MCF-7 and triple-negative breast cancer cell line; MDA-MB-231, adopting procedures of the sulforhodamine B colorimetric (SRB) assay [20]. Staurosporine was utilized as the reference drug for its well-known broad anticancer activity against diverse tumors. 

All of the tested indole derivatives exhibited gradual cellular log kill with IC_50_ values ranging from 0.44 μM to 47.1 μM against breast cancer cell line; MCF-7, while they exerted a much wider range of antiproliferative activity against MDA-MB-231 cell line with IC_50_ values ranging from 0.34 μM up to 77.30 μM, aside from compound **11** which showed very weak antiproliferative activity against MCF-7 cell line (IC_50_ = 84.70 μM) and no antiproliferative activity against MDA-MB-231 cell line (IC_50_ > 100 μM) in a proof of concept of the importance of oxindole moiety for boosting the antiproliferative activity.

Scrutinizing the IC_50_ values of series **7a**–**f** against MCF-7 cell line revealed that grafting a halide atom on the isatin moiety interestingly influences the antiproliferative activity, where the activity significantly decreased by increasing the size of the halide detected by the IC_50_ values of the floro (**5a**), chloro (**5b**), and bromo (**5c**) derivatives (IC_50_ = 1.53 μM, 8.87 μM, and 36.19 μM, respectively). This suggested that a floro substitution on the isatin group is advantageous for the antiproliferative activity where compound **5a** (IC_50_ = 1.53 μM) is 4.45 times more potent than the reference drug (IC_50_ = 6.81 μM), Table 1.

Furthermore, the influence of grafting electron-donating and electron-withdrawing groups within the oxindole moiety on the antiproliferative activity of the MCF-7 cell line was closely investigated. Interestingly, substitution with the electron-donating (CH_3_) group, compound **5d**, decreased the activity in comparison to Staurosporine (IC_50_ = 10.95 μM vs. 6.81 μM), whereas, grafting an electron-withdrawing nitro group on the oxindole moiety, compound **5e,** markedly enhanced the antiproliferative potency (IC_50_ = 0.44 μM), which is, fortunately, 15.5-times the potency of Staurosporine. Noteworthy, decoration of the oxindole moiety with 5,7-dimethyl substitution, compound **7f**, resulted in the abolishment of the growth inhibitory action toward MCF-7 cells (IC_50_ > 100 μM), Table 1. Conclusively, grafting a fluorine atom or a nitro group on C-5 of the oxindole moiety significantly boosts the activity against MCF-7 cells with a more pronounced effect for the nitro group.

As part of our work, we extended our investigation to explore the effect of substituting the nitrogen atom of the oxindole moiety by different alkyl and benzyl moieties in series **9**. Exploring the IC_50_ values unraveled that substitution of the nitrogen atom with an allyl group in **9a** significantly increased the activity 5.3-times compared to the reference drug (IC_50_ = 1.28 μM vs. 6.81 μM, respectively). Conversely, *N*-substitution with propyl group in **9b** (IC_50_ = 28.24 μM) and isobutyl group in **9c** (IC_50_ = 47.1 μM) markedly dwindled the growth inhibitory activity toward MCF-7 cell line.

Alternatively, substitution of the nitrogen atom with a benzyl moiety in **9d** (IC_50_ = 1.51 μM) significantly increased the activity 4.5-times in comparison to Staurosporine. Conversely, utilizing substituted benzyl groups in **9e** (4-F-benzyl) and **9f** (4-cyanobenzyl) was not advantageous for the activity as their IC_50_ values were less than the reference drug (IC_50_ = 10.43 μM and 8.72 μM, respectively). Moreover, two compounds (**9g** and **9h**) were synthesized as analogues of compounds **9d** and **9e**, where the oxindole moiety was further substituted with a bromo group in the 5-position. Investigation of the IC_50_ values of **9g** and **9h** (IC_50_ = 2.76 and 20.89 μM, respectively) clearly depicts a deterioration of the activity to the half as compared to **9d** and **9e**. This suggested that bromination of oxindole ring is not advantageous for the antiproliferative activity, an observation that is in accordance with the structure activity relationship extracted from series **7** (compound **7c**, IC_50_ = 36.19 μM).

Triple negative breast cancer (TNBC) is a stubborn type of cancer resistant to many chemotherapeutic agents, thus it represents a powerful challenge for medicinal chemists. Accordingly, we evaluated the potential antiproliferative activity for our compounds against TNBC cell line; MDA-MB-231 (Table 1). Analyzing the IC_50_ values of series **7a**–**f** and **9a**–**h** reveals very interesting results as many of the synthesized derivatives (**7a**, **7b**, **7d**, **7f**, **9d**, **9g** and **9h**) exhibited superior potencies compared to Staurosporine (IC_50_ = 9.04 μM, 2.88 μM, 0.34 μM, 1.32 μM, 4.14 μM, 2.85 μM, 2.29 μM and 10.29 μM, respectively). The IC_50_ values of series **7** unraveled that grafting a fluoro (**7a**) or a chloro (**7b**) group on the oxindole ring results in activity enhancement (IC_50_ = 9.04 μM and 2.88 μM, respectively), while grafting of a bromo group (**7c**) markedly decreased the activity (IC_50_ = 36.57 μM) in comparison to Staurosporine (IC_50_ = 10.29 μM). Moreover, substitution with a methyl group (**7d**) and a nitro group (**7e**) interestingly resulted in boosting the activity by 30.3- and 7.8-times, respectively. The effect of the *N*-substituent of the oxindole ring was further investigated in series **9.** Substitution of the nitrogen atom with an allyl group (**9a**) did not result in enhancement of the antiproliferative activity compared to Staurosporine (IC_50_ = 18.24 μM vs. 10.29 μM). Extending the substituent to propyl or isobutyl even worsens the case producing much less potent derivatives (**9b**) (IC_50_ = 51.27 μM) and (**9c**) which failed to produce any marked cytotoxic effect up to 100 μM. These results are in accordance with the observed results for series **7** where the addition of a larger or branched alkyl group proved to be detrimental to the antiproliferative action against MDA-MB-231 cell line (Table 1).

In addition, the impact of substitution of the nitrogen atom by un/substituted benzyl moieties was explored, revealing that unsubstituted benzyl moiety (**9d**) is advantageous for activity as it enhanced the activity by 2.5-times while utilizing 4-F-benzyl group (**9e**) or 4-CN-benzyl group (**9f**) resulted in a marked decrease of the activity compared to compound **9d** (IC_50_ = 17.66 μM and 25.41 μM vs. 4.14 μM). The antiproliferative activity for the 5-bromo substituted analogs (**9g** and **9h**) against the MDA-MB-231 cell line was compared to that of **9d** and **9e**. The results revealed that, in contrast to the results observed for the antiproliferative activity against MCF-7 cell line, the bromo substitution of the oxindole ring was advantageous for the activity as compound **9g** (IC_50_ = 2.85 μM) proved to be 1.5-times more potent than its unsubstituted bioisostere **9d**, also, compound **9h** (IC_50_ = 2.29 μM) proved even to be 7.7-times more potent than **9e** counterpart.

#### 2.2.2. In Vitro Cytotoxic Activity against Non-Tumorigenic Human Breast Cell Line

To investigate the selectivity and safety profile for the here reported bis-indoles toward the normal cells, compounds that displayed good activity towards MCF-7 and/or MDA-MB-231 cells were examined for their cytotoxic activity against non-tumorigenic human breast epithelial (MCF-10A) cell line (Table 2).

The examined bis-indoles exerted non-significant or modest cytotoxic action against non-tumorigenic MCF-10A cells with IC_50_ range: 14.06–54.92 µM, respectively. Bis-indoles **7e** and **9a** showed excellent selectivity indexes (SIs) equal to 38.7 and 18.3, respectively, whereas the remaining compounds, except **9h**, displayed good SIs spanning in the range 4.5–12.7 (Table 2).

#### 2.2.3. Cell Cycle Analysis

Anticancer agents exert their cytotoxic action by aborting cellular proliferation at certain checkpoints. These checkpoints are distinguishable phases in the cell cycle, whose suppression results in termination of the cell proliferation. To deeply comprehend the antiproliferative activity of our tested compounds, the most active two compounds (**7e** and **9a**) toward MCF-7 cells were further investigated for their effect on the different phases of the cell cycle in MCF-7 cell line. MCF-7 cells were treated with IC_50_ concentrations of the two compounds and their effect on the cell population in different cell phases was recorded and displayed in Table 3. Interestingly, exposure of MCF-7 cells to **7e** and **9a** resulted in marked augmentation in the proportion of cells in the G2/M phase by 3- and 2.21-fold, and in the Sub-G1 phase by 18.77- and 13.42-fold, respectively, in comparison to the control. This clearly indicates that the target bis-indoles arrested the cell cycle proliferation of MCF-7 cells in the G2/M phase.

#### 2.2.4. Effect of **5e**, **5f**, and **8a** on the Level of the Apoptotic Markers (Bax, Bcl-2, caspase-3, and p53)

**Figure 3:**
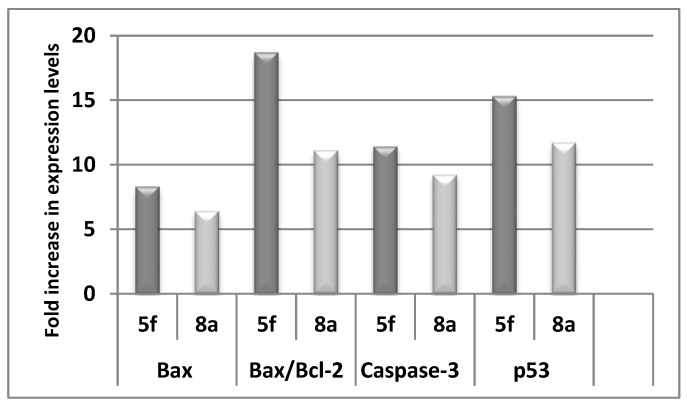
The numbers of fold increase in Bax/Bcl-2 ratio and expression levels of Bax, caspase-3, and p53 in MCF-7 cancer cells upon treatment with compounds **7e** and **9a** in comparison to the control.

Synchronization of the mitochondrial pathway is headed by the Bcl-2 family of proteins. These proteins are classified into two groups: anti-apoptotic proteins exemplified by Bcl-2 protein and the counteracting pro-apoptotic proteins including Bax protein [21]. As induction of the apoptotic machinery is one of the most useful strategies in cancer therapy [22,23], we investigated the effect of our compounds to boost the pro-apoptotic protein; Bax and reduce the anti-apoptotic protein; Bcl-2 in an attempt to explore the underlying mechanism for their cytotoxic activity (Table 1). As **7e** and **9a** proved to be the most active compounds, their effect on the level of Bax and Bcl-2 was investigated. Fortunately, both compounds markedly boosted the level of Bax by 8.3- and 6.4-fold, respectively (Table 4, Figure 3). Conformingly, they decreased the level of Bcl-2 by 2.25- and 1.74-fold, respectively. A more indicative parameter is the Bax/Bcl-2 ratio [24], which proved to be augmented by **7e** and **9a** 18.65- and 11.1-fold, respectively (Table 4, Figure 3). This further emphasizes that target bis-indoles trigger apoptosis by significantly boosting the Bax/Bcl-2 ratio.

Moreover, their effect on the level of caspase-3; the executioner caspase and p53 was evaluated in the MCF-7 cell line. Results revealed that **7e** and **9a** up-regulated the level of caspase-3 by 11.7- and 9.5-fold, respectively as compared to the control (Table 5). In addition, they augmented the level of p53 by 15.4- and 11.75-fold, respectively in comparison to the control (Table 5, Figure 3).

#### 2.2.5. Annexin V-FITC Apoptosis Assay

**Figure 4:**
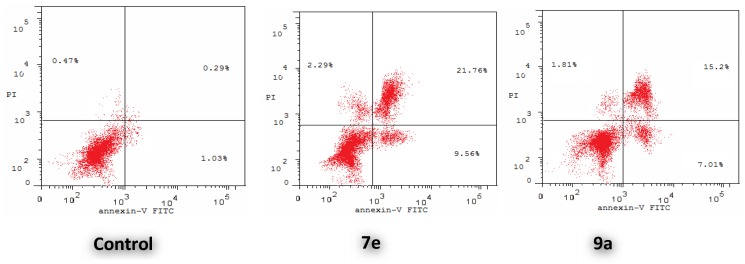
Influence of bis-indoles **7e** and **9a** on the percentage of annexin V-FITC-positive staining in MCF-7 cells. (Lower right: early apoptotic; upper right: late apoptotic; lower left: viable; upper left: necrotic).

Annexin V-based flow cytometry analysis is a useful tool to investigate whether cell death is pertaining to physiological apoptosis or nonspecific necrosis. Evaluation of the apoptotic effect of bis-indoles **7e** and **9a** was carried out using AnxV-FITC/DAPI dual staining assay (Figure 4). 

Treatment of MCF-7 cells with IC_50_ concentration of **7e** and **9a** exerted marked increase in the AnxV-FITC apoptotic cells percentage in both early (from 1.03% to 9.56% and 7.01%, respectively) and late apoptosis (from 0.29% to 21.76% and 15.20%, respectively) phases, Table 6. This corresponds to an increase in the total apoptosis percentage by 23.7-, and 16.8-fold, respectively compared to the control. This proved that the antiproliferative activity of here reported bis-indoles is due to physiological apoptosis, not nonspecific necrosis.

#### 2.2.6. CDK2 Inhibitory Activity

The efficient cell cycle disturbance influence of the herein reported bis-indoles (Table 7) prompted a more examination for their plausible inhibitory action toward the cell cycle regulator CDK2 protein kinase, in an attempt to gain further mechanistic insights for their promising growth inhibitory effect. The potent antiproliferative agents **7a**, **7b**, **7d**, **7e**, and **9a** were examined for their % inhibition of CDK2 at a single dose of 10 μM, (Table 7).

As displayed in Table 7, the examined bis-indoles exerted moderate to weak CDK2 inhibition with a % inhibition range of 16%–58%. Compound **7d** displayed the best % inhibition against CDK2 equals 58, whereas, both **7a** and **7b** showed % inhibition equals 44, Table 7. 

These results unveiled incompetence of the target bis-indoles to inhibit CDK2 significantly, highlighting that the cell growth inhibitory and cell cycle arrest capabilities of the target bis-indoles toward the examined human breast cancer cell lines is attributable to another target rather than CDK. Accordingly, further optimization for the herein reported bis-indoles including many mechanistic investigations are in progress and will be reported upon in the future.

## 3. Experimental

### 3.1. Chemistry

#### 3.1.1. General

Melting points were measured with a Stuart melting point apparatus and were uncorrected. Infrared spectra were recorded on Schimadzu FT-IR 8400S spectrophotometer. The NMR spectra were obtained on JEOL ECA-500 II spectrophotometer (500 MHz ^1^H and 125 MHz ^13^C NMR), in deuterated dimethylsulfoxide (DMSO-*d*_6_). Chemical shifts (*δ*_H_) are reported relative to TMS as the internal standard. All coupling constant (*J*) values are given in hertz. Chemical shifts (*δ*_C_) are reported relative to DMSO-*d_6_* as internal standards. Elemental analyses were carried out at the Regional Center for Microbiology and Biotechnology, Al-Azhar University. Compounds methyl 1*H*-indole-2-carboxylate **4** and 1*H*-indole-2-carbohydrazide **5** were prepared as reported earlier [25].

#### 3.1.2. General Procedure for Synthesis of the Target Bis-indoles (**7a**–**f** and **9a**–**h**), and **11**

To a hot stirred solution of key intermediate 1*H*-indole-2-carbohydrazide **5** (0.18 gm, 1 mmoL) in absolute ethyl alcohol (7 mL) and glacial acetic acid (catalytic amount), the appropriate *N*-unsubstituted 1*H*-indole-2,3-dione **6a**–**f**, *N*-substituted 1*H*-indole-2,3-dione **8a**–**h**, or 1-tetralone 10 (1 mmoL) was added. The resulting mixture was refluxed for 2 hours, and then the formed solid was filtered off while hot, washed with cold isopropyl alcohol, dried and recrystallized from DMF to afford target bis-indoles (**7a**–**f** and **9a**–**h**), and **11**, respectively.

##### *N′-(5-Fluoro-2-oxoindolin-3-ylidene)-2-(1H-indol-3-yl)acetohydrazide* (**7a**)

Red powder, m.p. 281–283 °C; (yield 70%), IR: 3327, 3270 (NH) and 1694 (C=O); ^1^H NMR *δ ppm*: 4.19 (brs, 2H, CH_2_-C=O), 6.85–6.88 (m, 1H, Ar-H), 6.95 (m, 1H, Ar-H), 7.05 (t, 1H, Ar-H, *J* = 7.5 Hz), 7.19 (t, 1H, Ar-H, *J* = 7.5 Hz), 7.32 (m, 2H, Ar-H), 7.58 (d, 1H, Ar-H, *J* = 7.5 Hz), 8.14 (s, 1H, Ar-H), 10.82 (s, 1H, NH of isatin, D_2_O exchangeable), 10.96 (s, 1H, NH of hydrazide, D_2_O exchangeable), 11.23 (s, 1H, NH of indol, D_2_O exchangeable); ^13^C NMR *δ ppm*: 28.26 (CH_2_-C=O), 107.26, 107.89, 111.33, 111.42, 113.06, 113.26, 115.58, 115.65, 18.52, 118.63, 121.07, 124.56, 127.28, 136.02, 138.54, 139.95, 156.56, 158.44, 162.57 (C=O of isatin), 164.81 (C=O of hydrazide); MS *m/z* [%]: 356.15 [M^+^, 100], 157.03 [11.35], 130.12 [25.10]; Anal. Calcd. for C_18_H_13_FN_4_O_2_: C, 64.28; H, 3.90; N, 16.66; found C, 63.93; H, 3.94; N, 16.73.

##### *N′-(5-Chloro-2-oxoindolin-3-ylidene)-2-(1H-indol-3-yl)acetohydrazide* (**7b**)

Orange powder, m.p. 292–294 °C; (yield 74%), IR: 3450, 3338 (NH) and 1694 (C=O); ^1^H NMR *δ ppm*: 4.11 (brs, 2H, CH_2_-C=O), 6.87 (d, 1H, Ar-H, *J* = 8.5 Hz), 6.96-6.99 (m, 1H, Ar-H), 7.05 (t, 1H, Ar-H, *J* = 7.5 Hz), 7.31-7.35 (m, 2H, Ar-H), 7.38 (d, 1H, Ar-H, *J* = 8.0 Hz), 7.57 (d, 1H, Ar-H, *J* = 8.0 Hz), 8.33 (s, 1H, Ar-H), 10.92 (s, 1H, NH of isatin, D_2_O exchangeable), 11.20 (s, 1H, NH of hydrazide, D_2_O exchangeable), 11.33 (s, 1H, NH of indol, D_2_O exchangeable); Anal. Calcd. for C_18_H_13_ClN_4_O_2_: C, 61.28; H, 3.71; N, 15.88; found C, 60.95; H, 3.66; N, 15.97.

##### *N′-(5-Bromo-2-oxoindolin-3-ylidene)-2-(1H-indol-3-yl)acetohydrazide* (**7c**)

Orange powder, m.p. > 300 °C; (yield 85%), IR: 3365, 3219, 3182 (NH) and 1726, 1692 (C=O); ^1^H NMR *δ ppm*: 4.11 (brs, 2H, CH_2_-C=O), 6.83 (d, 1H, Ar-H, *J* = 8.5 Hz), 6.96–6.99 (m, 1H, Ar-H), 7.04 (t, 1H, Ar-H, *J* = 7.5 Hz), 7.31-7.35 (m, 2H, Ar-H), 7.51 (d, 1H, Ar-H, *J* = 8.0 Hz), 7.57 (d, 1H, Ar-H, *J* = 8.0 Hz), 8.43 (s, 1H, Ar-H), 10.93 (s, 1H, NH of isatin, D_2_O exchangeable), 10.96 (s, 1H, NH of hydrazide, D_2_O exchangeable), 11.33 (s, 1H, NH of indol, D_2_O exchangeable); Anal. Calcd. for C_18_H_13_BrN_4_O_2_: C, 54.43; H, 3.30; N, 14.10; found C, 54.85; H, 3.28; N, 14.02.

##### *2-(1H-Indol-3-yl)-N′-(5-methyl-2-oxoindolin-3-ylidene)acetohydrazide* (**7d**)

Red powder, m.p. > 300 °C; (yield 78%), IR: 3363, 3309, 3191 (NH) and 1691 (C=O); ^1^H NMR *δ ppm*: 2.24, 2.50 (2s, 3H, CH_3_), 3.87, 4.18 (2s, 2H, CH_2_-C=O), 6.76-6.81 (m, 1H, Ar-H), 6.97 (t, 1H, Ar-H, *J* = 7.5 Hz), 7.07-7.14 (m, 2H, Ar-H), 7.30–7.59 (m, 4H, Ar-H), 10.95 (s, 1H, NH of isatin, D_2_O exchangeable), 11.08, 11.12 (2s, 1H, NH of hydrazide, D_2_O exchangeable), 12.51, 12.96 (2s, 1H, NH of indol, D_2_O exchangeable); ^13^C NMR *δ ppm*: 18.57, 20.53, 18.14, 32.32, 106.38, 107.03, 110.83, 111.42, 111.52, 118.45, 118.67, 119.87, 120.92, 121.04, 121.29, 124.32, 124.81, 126.99, 127.27, 131.61, 131.79, 140.02, 162.55, 168.32, 173.32; MS *m/z* [%]: 332.11 [M^+^, 80.08], 157.24 [40.03], 130.19 [100]; Anal. Calcd. for C_19_H_16_N_4_O_2_: C, 68.66; H, 4.85; N, 16.86; found C, 68.83; H, 4.80; N, 16.97.

##### *2-(1H-Indol-3-yl)-N′-(5-nitro-2-oxoindolin-3-ylidene)acetohydrazide* (**7e**)

Yellow powder, m.p. 289–291 °C; (yield 80%), IR: 3399, 3147 (NH) and 1728, 1686 (C=O); ^1^H NMR *δ ppm*: 4.13 (brs, 2H, CH_2_-C=O), 6.95-7.00 (m, 1H, Ar-H), 7.04–7.08 (m, 2H, Ar-H), 7.33–7.35 (m, 2H, Ar-H), 7.58 (d, 1H, Ar-H, *J* = 8.0 Hz), 8.19-8.28 (m, 1H, Ar-H), 9.04 (s, 1H, Ar-H), 10.96 (s, 1H, NH of isatin, D_2_O exchangeable), 11.50 (s, 1H, NH of hydrazide, D_2_O exchangeable), 11.81 (s, 1H, NH of indol, D_2_O exchangeable); ^13^C NMR *δ ppm*: 18.57 (CH_2_-C=O), 111.31, 111.40, 111.49, 115.08, 115.62, 118.51, 118.63, 120.70, 121.03, 121.48, 127.21, 136.00, 136.12, 141.99, 142.75, 147.42, 149.13, 162.76 (C=O of isatin), 165.04 (C=O of hydrazide); Anal. Calcd. for C_18_H_13_N_5_O_4_: C, 59.50; H, 3.61; N, 19.28; found C, 59.67; H, 3.57; N, 9.34.

##### *N′-(5,7-Dimethyl-2-oxoindolin-3-ylidene)-2-(1H-indol-3-yl)acetohydrazide* (**7f**)

Brown powder, m.p. 294–296 °C; (yield 73%), IR: 3432, 3267, 3180 (NH) and 1725, 1692 (C=O); ^1^H NMR *δ ppm*: 2.13 (s, 3H, CH_3_ of isatin), 2.20 (s, 3H, CH_3_ of isatin), 4.14 (brs, 2H, CH_2_-C=O), 6.96–6.99 (m, 2H, Ar-H), 7.07 (brs, 1H, Ar-H), 7.35 (br s, 2H, Ar-H), 7.58 (d, 1H, Ar-H, *J* = 8.0 Hz), 7.84 (s, 1H, Ar-H), 10.71 (s, 1H, NH of isatin, D_2_O exchangeable), 10.98 (s, 1H, NH of hydrazide, D_2_O exchangeable), 11.06 (s, 1H, NH of indol, D_2_O exchangeable); ^13^C NMR *δ ppm*: 15.80, 15.99, 20.34, 107.21, 111.40, 111.48, 115.01, 118.32, 118.59, 119.59, 121.05, 121.27, 124.67, 127.28, 130.55, 131.55, 134.05, 136.05, 139.85, 162.20; Anal. Calcd. for C_20_H_18_N_4_O_2_: C, 69.35; H, 5.24; N, 16.17; found C, 69.46; H, 5.20; N, 16.11.

##### *N′-(1-Allyl-2-oxoindolin-3-ylidene)-2-(1H-indol-3-yl)acetohydrazide* (**9a**)

Orange powder, m.p. 218–220 °C; (yield 75%), IR: 3354, 3238 (NH) and 1704 (C=O); ^1^H NMR *δ ppm*: 4.12 (brs, 2H, CH_2_-C=O), 4.36 (s, 2H, *N*-CH_2_), 5.13-5.16 (m, 2H, CH_2_=CH-), 5.82-5.87 (m, 1H, *N*-CH_2_-CH), 6.96-7.09 (m, 4H, Ar-H), 7.34 (s, 2H, Ar-H), 7.39 (t, 1H, Ar-H, *J* = 8.0 Hz), 7.58 (d, 1H, Ar-H, *J* = 8.0 Hz), 8.16 (s, 1H, Ar-H), 10.99 (s, 1H, NH of hydrazide, D_2_O exchangeable), 11.02 (s, 1H, NH of indol, D_2_O exchangeable); ^13^C NMR *δ ppm*: 29.84 (CH_2_-C=O), 41.48 (*N*-CH_2_), 107.22, 109.84, 111.45, 114.73, 116.93, 118.64, 121.15, 122.18, 124.62, 125.60, 131.80, 132.27, 136.06, 143.75, 163.09; MS *m/z* [%]: 358.23 [M^+^, 41.07], 157.15 [34.19], 130.26 [100]; Anal. Calcd. for C_21_H_18_N_4_O_2_: C, 70.38; H, 5.06; N, 15.63; found C, 70.21; H, 5.13; N, 15.75.

##### *2-(1H-Indol-3-yl)-N′-(2-oxo-1-propylindolin-3-ylidene)acetohydrazide* (**9b**)

Orange powder m.p. 207–208 °C; (yield 77%), IR: 3317, 3282 (NH) and 1705 (C=O); ^1^H NMR *δ ppm*: 0.85 (brs, 3H, CH_2_-CH_3_), 1.59 (brs, 2H, CH_2_-CH_3_), 3.68 (t, 2H, *N*-CH_2_), 3.89, 4.20 (2s, 2H, CH_2_-C=O), 6.96 (t, 1H, Ar-H, *J* = 7.5 Hz), 7.07–7.19 (m, 2H, Ar-H), 7.30–7.43 (m, 4H, Ar-H), 7.58 (d, 1H, Ar-H, *J* = 7.5 Hz), 7.69 (s, 1H, Ar-H), 10.95, 11.09 (2s, 1H, NH of hydrazide, D_2_O exchangeable), 12.44, 12.91 (2s, 1H, NH of indol, D_2_O exchangeable); Anal. Calcd. for C_21_H_20_N_4_O_2_: C, 69.98; H, 5.59; N, 15.55; found C, 70.12; H, 5.62; N, 15.43.

##### *N′-(1-(sec-Butyl)-2-oxoindolin-3-ylidene)-2-(1H-indol-3-yl)acetohydrazide* (**9c**)

Orange powder, m.p. 199–201 °C; (yield 68%), IR: 3451, 3294 (NH) and 1707 (C=O); ^1^H NMR *δ ppm*: 0.87 (d, 6H, -CH(CH_3_)_2_, *J* = 2.5 Hz), 2.03 (brs, 1H, -CH(CH_3_)_2_), 3.52 (d, 2H, *N*-CH_2_-CH(CH_3_)_2_, *J* = 2.5 Hz), 4.13 (brs, 2H, CH_2_-C=O), 6.97–7.07 (m, 3H, Ar-H), 7.12 (d, 1H, Ar-H, *J* = 8.5 Hz), 7.34 (brs, 2H, Ar-H), 7.39 (t, 1H, Ar-H, *J* = 8.5 Hz), 7.59 (d, 1H, Ar-H, *J* = 7.5 Hz), 8.14 (brs, 1H, Ar-H), 10.98 (s, 1H, NH of hydrazide, D_2_O exchangeable), 11.18 (s, 1H, NH of indol, D_2_O exchangeable); ^13^C NMR *δ ppm*: 19.91 (-CH(CH_3_)_2_), 26.62 (-CH(CH_3_)_2_), 46.58 (*N*-CH_2_), 107.23, 109.74, 111.44, 114.60, 118.66, 121.12, 122.01, 124.61, 125.58, 127.24, 132.31, 132.45, 136.06, 144.38, 163.55; MS *m/z* [%]: 374.23 [M^+^, 100], 157.10 [18.76], 130.38 [59.05]; Anal. Calcd. for C_22_H_22_N_4_O_2_: C, 70.57; H, 5.92; N, 14.96; found C, 70.69; H, 5.87; N, 15.02. 

##### *N′-(1-Benzyl-2-oxoindolin-3-ylidene)-2-(1H-indol-3-yl)acetohydrazide* (**9d**)

Red powder, m.p. 223–225 °C; (yield 76%), IR: 3308, 3245 (NH) and 1706 (C=O); ^1^H NMR *δ ppm*: 4.15 (brs, 2H, CH_2_-C=O), 4.96 (s, 2H, benzylic protons), 6.98 (d, 3H, Ar-H, *J* = 7.5 Hz), 7.06 (t, 1H, Ar-H, *J* = 7.5 Hz), 7.25-7.35 (m, 8H, Ar-H), 7.60 (d, 1H, Ar-H, *J* = 8.5 Hz), 8.17 (brs, 1H, Ar-H), 10.99 (s, 1H, NH of hydrazide, D_2_O exchangeable), 11.23 (s, 1H, NH of indol, D_2_O exchangeable); ^13^C NMR *δ ppm*: 42.64, 107.22, 109.86, 111.45, 114.82, 118.66, 121.14, 122.32, 124.64, 125.68, 127.23, 127.51, 128.72, 132.23, 136.08, 136.18, 143.60, 163.54; MS *m/z* [%]: 408.25 [M^+^, 32.23], 157.11 [100], 130.05 [48.95]; Anal. Calcd. for C_25_H_20_N_4_O_2_: C, 73.51; H, 4.94; N, 13.72; found C, 73.63; H, 4.91; N, 13.63.

##### *N′-(1-(4-Fluorobenzyl)-2-oxoindolin-3-ylidene)-2-(1H-indol-3-yl)acetohydrazide* (**9e**)

Red powder, m.p. 227–229 °C; (yield 72%), IR: 3353, 3115 (NH) and 1723 (C=O); ^1^H NMR *δ ppm*: 4.15 (brs, 2H, CH_2_-C=O), 4.94 (s, 2H, benzylic protons), 6.95–7.02 (m, 3H, Ar-H), 7.06 (t, 1H, Ar-H, *J* = 8.0 Hz), 7.13 (t, 2H, Ar-H, *J* = 8.0 Hz), 7.34–7.37 (m, 5H, Ar-H), 7.59 (d, 1H, Ar-H, *J* = 7.5 Hz), 8.16 (brs, 1H, Ar-H), 10.99 (s, 1H, NH of hydrazide, D_2_O exchangeable), 11.23 (s, 1H, NH of indol, D_2_O exchangeable); Anal. Calcd. for C_25_H_19_FN_4_O_2_ (344.38): C, 70.41; H, 4.49; N, 13.14; found C, 70.73; H, 4.46; N, 13.11.

##### *N′-(1-(4-Cyanobenzyl)-2-oxoindolin-3-ylidene)-2-(1H-indol-3-yl)acetohydrazide* (**9f**)

Yellow powder, m.p. 233–234 °C; (yield 80%), IR: 3290, 3247 (NH), 2230 (C≡N) and 1704 (C=O); ^1^H NMR *δ ppm*: 4.14 (brs, 2H, CH_2_-C=O), 4.95 (s, 2H, benzylic protons), 6.99 (d, 2H, Ar-H, *J* = 7.5 Hz), 7.06 (t, 2H, Ar-H, *J* = 7.5 Hz), 7.34 (t, 3H, Ar-H, *J* = 7.5 Hz), 7.52 (t, 1H, Ar-H, *J* = 7.5 Hz), 7.60 (d, 1H, Ar-H, *J* = 7.5 Hz), 7.64 (d, 1H, Ar-H, *J* = 7.5 Hz), 7.73 (d, 1H, Ar-H, *J* = 7.5 Hz), 7.85 (s, 1H, Ar-H), 8.14 (brs, 1H, Ar-H), 11.00 (s, 1H, NH of hydrazide, D_2_O exchangeable), 11.24 (s, 1H, NH of indol, D_2_O exchangeable); ^13^C NMR *δ ppm*: 42.01 (CH_2_), 107.21, 109.68, 111.46, 111.62, 115.02, 118.63, 122.46, 123.28, 124.64, 129.90, 129.98, 130.91, 131.40, 132.10, 137.96, 142.32, 143.32, 160.79 (C=O of isatin), 163.67 (C=O of hydrazide); Anal. Calcd. for C_26_H_19_N_5_O_2_: C, 72.04; H, 4.42; N, 16.16; found C, 71.82; H, 4.48; N, 16.28.

##### *N′-(1-Benzyl-5-bromo-2-oxoindolin-3-ylidene)-2-(1H-indol-3-yl)acetohydrazide* (**9g**)

Orange powder, m.p. 210–211 °C; (yield 82%), IR: 3367, 3282 (NH) and 1739, 1669 (C=O); ^1^H NMR *δ ppm*: 4.14 (brs, 2H, CH_2_-C=O), 4.96 (s, 2H, benzylic protons), 6.93 (d, 1H, Ar-H, *J* = 8.0 Hz), 6.96 (t, 1H, Ar-H, *J* = 7.5 Hz), 7.05 (t, 1H, Ar-H, *J* = 7.5 Hz), 7.25–7.36 (m, 7H, Ar-H), 7.53 (d, 1H, Ar-H, *J* = 8.0 Hz), 7.59 (d, 1H, Ar-H, *J* = 8.0 Hz), 8.47 (brs, 1H, Ar-H), 10.97 (s, 1H, NH of hydrazide, D_2_O exchangeable), 11.48 (s, 1H, NH of indol, D_2_O exchangeable); ^13^C NMR *δ ppm*: 42.71 (CH_2_), 107.21, 111.44, 111.62, 114.35, 116.46, 118.53, 118.63, 121.06, 124.54, 127.19, 127.55, 128.03, 1289.74, 134.24, 135.89, 136.01, 142.65, 163.26; Anal. Calcd. for C_25_H_19_BrN_4_O_2_: C, 61.61; H, 3.93; N, 11.50; found C, 61.79; H, 3.88; N, 11.57.

##### *N′-(5-Bromo-1-(4-fluorobenzyl)-2-oxoindolin-3-ylidene)-2-(1H-indol-3-yl)acetohydrazide* (**9h**)

Brown powder, m.p. 183–185 °C; (yield 80%), IR: 3423, 3343 (NH) and 1730 (C=O); ^1^H NMR *δ ppm*: 4.13 (s, 2H, CH_2_-C=O), 4.95 (s, 2H, benzylic protons), 6.97 (d, 2H, Ar-H, *J* = 8.5 Hz), 7.05 (t, 1H, Ar-H, *J* = 8.0 Hz), 7.13 (t, 2H, Ar-H, *J* = 8.5 Hz), 7.33–7.37 (m, 4H, Ar-H), 7.55 (d, 1H, Ar-H, *J* = 8.0 Hz), 7.58 (d, 1H, Ar-H, *J* = 7.5 Hz), 8.45 (s, 1H, Ar-H), 10.97 (s, 1H, NH of hydrazide, D_2_O exchangeable), 11.49 (s, 1H, NH of indol, D_2_O exchangeable); Anal. Calcd. for C_25_H_18_BrFN_4_O_2_: C, 59.42; H, 3.59; N, 11.09; found C, 59.58; H, 3.55; N, 11.16.

##### *N′-(3,4-Dihydronaphthalen-1(2H)-ylidene)-2-(1H-indol-3-yl)acetohydrazide* (**11**)

White crystals, m.p. 244–245 °C; (yield 84%), IR: 3315, 3246 (NH) and 1692 (C=O); ^1^H NMR *δ ppm*: 1.81 (d, 2H, CH_2_, *J* = 6.0 Hz), 2.61 (m, 2H, CH_2_), 2.74 (d, 2H, CH_2_, *J* = 5.6 Hz), 3.78, 4.13 (2s, 2H, CH_2_-C=O), 6.95–7.09 (m, 2H, Ar-H), 7.19–7.27 (m, 4H, Ar-H), 7.34 (t, 1H, Ar-H, *J* = 7.6 Hz), 7.57, 7.62 (2d, 1H, Ar-H, *J* = 8.0, 8.0 Hz), 8.00, 8.10 (2d, 1H, Ar-H, *J* = 7.2, 7.6 Hz), 10.41, 10.43 (s, 1H, NH of hydrazide, D_2_O exchangeable), 10.86, 10.90 (s, 1H, NH of indol, D_2_O exchangeable); Anal. Calcd. for C_20_H_19_N_3_O: C, 75.69; H, 6.03; N, 13.24; found C, 75.86; H, 5.99; N, 13.32.

### 3.2. Biological Evaluation

The detailed experimental procedures adopted in the different biological assays for target bis-indoles (**7a**–**f** and **9a**–**h**); **11** were supplied in the Supplementary Materials.

#### 3.2.1. Cytotoxic Activity against Human Breast Cancer and Non-Tumorigenic Cell Lines

The two examined human breast cancer cell lines (MCF-7 and Breast MDA-MB-231), and non-tumorigenic human breast epithelial cell line (MCF-10A) have been obtained from the American Type Culture Collection (ATCC). Assessment of cytotoxicity for target indole derivatives has been performed following the SRB colorimetric assay procedures [20], as reported earlier [26].

#### 3.2.2. Cell Cycle Analysis

The influence of bis-indoles **7e** and **9a** on cell cycle progression was examined in breast cancer MCF-7 cells, after 24 h of treatment, through DNA flow cytometric assay by the use of BD FACS Caliber flow cytometer, as described previously [27]. The cell cycle distributions were calculated using CellQuest software (Becton Dickinson).

#### 3.2.3. ELISA Immunoassay

Effects of treatment of breast cancer MCF-7 cells with bis-indoles **7e** and **9a** on the expression levels of the pro-apoptotic markers (Bax, caspase-3, and p53) in addition to the anti-apoptotic protein Bcl-2 marker was assessed by the use of ELISA colorimetric kits as per the manufacturer’s instructions, as described earlier [28].

#### 3.2.4. Annexin V-FITC/PI Apoptosis Assay

The apoptotic action of bis-indoles **7e** and **9a** was further explored through the investigation of their effect on the phosphatidylserine externalization in breast cancer MCF-7 cells, using Annexin-V-FITC Apoptosis Detection Kit according to manufacturer’s protocol, as reported earlier [27].

#### 3.2.5. CDK2 Kinase Inhibitory Activity

The in vitro CDK2 kinase inhibition assay was carried out by Reaction Biology Corp. (Reaction Biology Corp., Chester, PA, USA) Kinase HotSpotSM service (http://www.reactionbiology.com).

## 4. Conclusions

In summary, the adopted approach of replacing the rigid heterocyclic spacer in the marine natural products Topsentin and Nortopsentin by the flexible hydrazide linker resulted in the discovery of promising marine-inspired bis-indole scaffold with good in vitro antitumor activities toward breast cancer cell lines. All the synthesized bis-indoles (**7a**–**f** and **9a**–**h**), and indole **11** were characterized for their antiproliferative action against human breast cancer (MCF-7 and MDA-MB-231) cell lines. All the examined bis-indoles **7a**–**f** and **9a**–**h** displayed excellent to low antiproliferative activities against MCF-7 and MDA-MB-231 cells with IC_50_ values in ranges 0.44–47.1 and 0.34–77.30 μM, respectively. Bioisosteric replacement of the oxindole ring with the carbocyclic tetralin ring (compound **11**) resulted in a dramatic worsening of effectiveness against the examined cancer cell lines (IC_50_ = 84.70 ± 4.02 and > 100 μM, respectively) in comparison to bis-indoles **7**, which pointed out the importance of oxindole moiety for the antiproliferative activity. The most potent congeners **7e** and 9a against MCF-7 cells (IC_50_ = 0.44 ± 0.01 and 1.28 ± 0.04 μM, respectively) induced apoptosis in MCF-7 cells (23.7-, and 16.8-fold increase in the total apoptosis percentage) as evident by the externalization of plasma membrane phosphatidylserine detected by AnnexinV-FITC/PI assay. This evidence was supported by the Bax/Bcl-2 ratio augmentation (18.65- and 11.1-fold compared to control) with a concomitant increase in the level of caspase-3 (11.7- and 9.5-fold) and p53 (15.4- and 11.75-fold). Moreover, scrutinizing results of the cell cycle analysis unraveled that both compounds arrest the cell cycle mainly in the G2/M phase. On the other hand, **7e** and **9a** displayed good selectivity toward tumor cells (*S.I*. = 38.7 and 18.3), upon testing of their cytotoxicity toward non-tumorigenic breast MCF-10A cells. Finally, compounds **7a**, **7b**, **7d**, **7e**, and **9a** were examined for their plausible CDK2 inhibitory action. The obtained results (% inhibition range: 16%–58%) unveiled incompetence of the target bis-indoles to inhibit CDK2 significantly. Collectively, these results suggested that herein reported bis-indoles are good lead compounds for further optimization and development as potential efficient anti-breast cancer drugs. 

## Figures and Tables

**Table 1:** In vitro antiproliferative activity of **7a**–**f**, **9a**–**h**, and **11** against breast MCF-7 and MDA-MB-231 cancer cell lines.

Cpd.	R	R_1_	R_2_	IC_50_ (µM) ^a^	
				MCF-7	MDA-MB-231
**7a**	**F**	**H**	-	1.53 ± 0.02	9.04 ± 0.32
**7b**	Cl	H	-	8.87 ± 0.43	2.88 ± 0.08
**7c**	Br	H	-	36.19 ± 2.78	36.57 ± 1.81
**7d**	CH_3_	H	-	10.95 ± 0.81	0.34 ± 0.02
**7e**	NO_2_	H	-	0.44 ± 0.01	1.32 ± 0.03
**7f**	CH_3_	CH_3_	-	NA ^b^	77.30 ± 6.21
**9a**	H	-	−CH_2_CH=CH_2_	1.28 ± 0.04	18.24 ± 0.62
**9b**	H	-	−CH_2_CH_2_CH_3_	28.24 ± 1.53	51.27 ± 3.59
**9c**	H	-	−CH_2_CH(CH_3_)_2_	47.10 ± 3.65	NA ^b^
**9d**	H	-	−CH_2_C_6_H_5_	1.51 ± 0.03	4.14 ± 0.19
**9e**	H	-	−CH_2_C_6_H_4_-4-F	10.43 ± 0.81	17.66 ± 0.55
**9f**	H	-	−CH_2_C_6_H_4_-4-CN	8.72 ± 0.39	25.41 ± 1.56
**9g**	Br	-	−CH_2_C_6_H_5_	2.76 ± 0.14	2.85 ± 0.07
**9h**	Br	-	−CH_2_C_6_H_4_-4F	20.89 ± 0.04	2.29 ± 0.09
**11**	-	-	-	84.70 ± 4.02	NA ^b^
**Staurosporine**	-	-	-	6.81 ± 0.22	10.29 ± 0.72

**^a^** IC_50_ values are the mean ± S.D. of three separate experiments. ^b^ NA: Compounds having IC_50_ value > 100 µM.

**Table 2:** Cytotoxic activity toward non-tumorigenic human breast MCF-10A cell line, and selectivity index (MCF-10A/MCF-7).

Comp.	IC_50_ (µM)		Selectivity Index
	MCF-10A	MCF-7	MCF-10A/MCF-7
**7a**	14.06	1.53	9.2
**7b**	39.54	8.87	4.5
**7d**	54.92	10.95	5.0
**7e**	17.06	0.44	38.7
**9a**	23.47	1.28	18.3
**9d**	19.12	1.51	12.7
**9e**	48.39	10.43	4.7
**9f**	42.01	8.72	4.8
**9g**	17.16	2.76	6.2
**9h**	26.09	20.89	1.2

**Table 3:** Effect of compounds **7e** and **9a** on the phases of the cell cycle of MCF-7 cells.

Comp.	%G0-G1	%S	%G2/M	%Sub-G1
**7e**	31.66	25.44	42.9	33.61
**9a**	43.82	24.91	31.27	24.02
**Control**	57.26	28.59	14.15	1.79

**Table 4:** Effect of bis-indoles **7e** and **9a** on the expression levels of Bcl-2 and Bax in MCF-7 cancer cells.

Compound	Bax (pg/mg of Total Protein)	Bcl-2 (ng/mg of Total Protein)	Bax/Bcl-2
**7e**	318.0 ± 10.5	2.07 ± 0.14	153.6
**9a**	243.6 ± 12.4	2.67 ± 0.16	91.2
**Control**	38.3 ± 2.2	4.65 ± 0.23	8.2

**Table 5:** Effect of compounds **7e** and **9a** on the expression levels of active caspase-3 and p53 in MCF-7 cancer cells.

Compound	Caspase-3 (pg/mg)	p53 (pg/mg)
**7e**	409.2 ± 17.2	631.8 ± 35.8
**9a**	331.0 ± 12.5	482.3 ± 27.4
**Control**	35.92 ± 1.8	41.26 ± 2.7

**Table 6:** Distribution of apoptotic cells in the AnnexinV-FITC/PI dual staining assay in MCF-7 cells after treatment with bis-indoles **7e** and **9a**.

Compound	Early Apoptosis (Lower Right %)	Late Apoptosis (Upper Right %)	Total (L.R % + U.R %)
**7e**	9.56	21.76	31.32
**9a**	7.01	15.20	22.21
**Control**	1.03	0.29	1.32

**Table 7:** Inhibitory effect of bis-indoles **7a**, **7b**, **7d**, **7e**, and **9a** against CDK2 kinase activity at a single dose of 10 µM.

Compound	% Enzyme Inhibitory Activity
**7a**	41
**7b**	41
**7d**	58
**7e**	16
**9a**	23
**Staurosporine**	99

## Supplementary Materials

The following are available online at https://www.mdpi.com/1660-3397/18/4/190/s1.
