# Supplementary material for: Marine-Inspired Bis-indoles Possessing Antiproliferative Activity against Breast Cancer; Design, Synthesis, and Biological Evaluation
Source: Mar Drugs. 2020 Apr 2;18(4):190. doi: 10.3390/md18040190 (PMC7230731; doi:10.3390/md18040190)
Supplement: Supplementary file 1 [file marinedrugs-18-00190-s001.pdf]

Supporting Information

Marine-Inspired Bis-indoles Possessing Anti-proliferative Activity against Breast Cancer; Design, Synthesis and Biological Evaluation

Wagdy M. Eldehna, Ghada S. Hassan, Sara T. Al-Rashood, Hamad M. Alkahtani, Abdulrahman A. Almehizia, Ghada H. Al-Ansary

1. Anti-proliferative action toward human breast cell lines. 2
2. Cell Cycle Analysis. 2
3. ELISA Immunoassay. 3
4. Annexin V-FITC Apoptosis Assay. 3
5. CDK2 kinase inhibitory activity. 4
6. Representative Mass, NMR and IR spectra. 5-36

Biological Evaluations

1. Anti-proliferative action toward human breast cell lines

The two examined human breast cancer cell lines (MCF-7 and Breast MDA-MB-231), and non-tumorigenic human breast epithelial cell line (MCF-10A) have been obtained from American Type Culture Collection (ATCC). The cells were maintained in Dulbecco's modified Eagle's medium (DMEM) supplemented with 10% heat inactivated fetal calf serum (GIBCO), penicillin (100 U/ml) and streptomycin (100 μg/ml) at 37 ^{o}C in humidified atmosphere containing 5% CO_2. Cells at a concentration of 0.50 x 10^6 were grown in a 25 cm^2 flask in 5 ml of culture medium.

The anti-proliferative activity of the tested compounds was measured *in vitro* using the Sulfo-Rhodamine-B stain (SRB) assay. Briefly, Cells were inoculated in 96-well microtiter plate (5X10^4 cells/ well) for 24 h before treatment with the tested compounds to allow attachment of cell to the wall of the plate. Tested compounds were dissolved in DMSO at 1 mg/ml immediately before use and diluted to the appropriate volume just before addition to the cell culture. Different concentrations of tested compounds, doxorubicin and sorafenib were added to the cells (three wells were prepared for each individual dose). Cells were incubated with the compounds for 48 h at 37°C and in atmosphere of 5% CO_2. After 48 h cells were fixed, washed, and stained for 30 min with 0.4% (w/v) SRB dissolved in 1% acetic acid. Unbound dye was removed by four washes with 1% acetic acid, and attached stain was recovered with Tris-EDTA buffer. Color intensity was measured in an ELISA reader. The relation between percent of surviving fraction and drug concentration is plotted to get the survival curve for each cell line. The concentration required for 50% inhibition of cell viability (IC_{50}) was calculated.

2. Cell Cycle Analysis

Breast cancer MCF-7 cells were treated with bis-indoles **7e** and **9a** for 24 h (at their IC_{50} concentration), and then cells were washed twice with ice-cold phosphate buffered saline (PBS). Subsequently, the treated cells were collected by centrifugation, fixed in ice-cold 70% (*v/v*) ethanol, washed with PBS, re-suspended with 100 μg/mL RNase, stained with 40 μg/mL PI, and analyzed by flow cytometry using FACS Calibur (Becton Dickinson, BD, Franklin Lakes, NJ, USA). The cell cycle distributions were calculated using CellQuest software 5.1 (Becton Dickinson).

3. ELISA Immunoassay

The levels of the pro-apoptotic markers (Bax, caspase-3 and p53) as well as the anti-apoptotic marker Bcl-2 were determined using ELISA colorimetric kits per the manufacturer's instructions. Breast cancer MCF-7 cells were cultured as a monolayer in T-25 flasks and were seeded to attain 30% confluency prior to treatment. Cells were then treated separately with bis-indoles **7e** and **9a** at their IC_{50} concentrations for 48 h. At the end of treatment, cells were collected via trypsinization and centrifuged at 10,000 rpm. The pellet was then rinsed with PBS and lysed in RIPA lysis buffer at 4 °C for 45 min, then centrifuged at 14,000 rpm for 20 min to remove the cellular debris. Lysates were then collected and stored at −80 °C for later protein determination using Pierce BCA Protein Assay Kit according to manufacturer's recommendations.

The cell lysate was diluted 10 times, and 100 μL (50 mg protein) was added to the wells of four separate microtiter plates for the four ELISA kits that were pre-coated with primary antibodies specific to Bax, Bcl-2, caspase-3 and p53 proteins, respectively. A secondary biotin-linked antibody specific to the protein captured by the primary antibody was further added to bind the captured protein, forming a "sandwich" of specific antibodies around the desired protein in the cell lysate. The streptavidin-HRP complex was then used to bind the biotin-linked secondary antibody through its streptavidin portion. The HRP domain reacted with the added TMB substrate to form a colored product that measured at 450 nm by a plate reader ChroMate-4300 after the reaction was terminated *via* the addition of stop solution.

4. Annexin V-FITC Apoptosis Assay

Phosphatidylserine externalization was assayed using Annexin V-FITC/PI apoptosis detection kit (BD Biosciences, USA) according to the manufacturer's instructions. Breast cancer MCF-7 cells were cultured to a monolayer then treated with bis-indoles **7e** and **9a** at their IC_{50} concentration. Briefly, cells were then harvested *via* trypsinization, and rinsed twice in PBS followed by binding buffer. Moreover, cells were re-suspended in 100 μL of binding buffer with the addition of 1 μL of FITC-Annexin V followed by an incubation period of 30 min at 4 °C. Cells were then rinsed in binding buffer and resuspended in 150 μL of binding buffer with the addition of 1 μL of DAPI (1 μg/μL in PBS). Cells were then analyzed using the flow cytometer BD FACS Canto II and the results were interpreted with FlowJo7.6.4 software (Tree Star, Ashland, OR, USA).

5. CDK2 kinase inhibitory activity

Reaction Biology Corp. Kinase HotSpotSM service (http://www.reactionbiology.com) was used for screening of tested compounds. CDK2 Kinase inhibitory activity was assessed by the HotSpot assay platform, which contained specific kinase/substrate pairs along with required cofactors. Base reaction buffer: 20 mM Hepes (pH 7.5), 10 mM $MgCl_2$, 1 mM EGTA, 0.02% Brij35, 0.02 mg/ml BSA, 0.1 mM Na_3VO_4, 2 mM DTT, 1% DMSO. Testing compounds were dissolved in 100% DMSO to specific concentration. The serial dilution was conducted by Integra Viaflo Assist in DMSO. The reaction mixture containing the examined compound and 33P-ATP was incubated at room temperature for 2 h and radioactivity was detected by filter-binding method. Kinase activity data were expressed as the percent remaining kinase activity in test samples compared to vehicle (dimethyl sulfoxide) reactions.

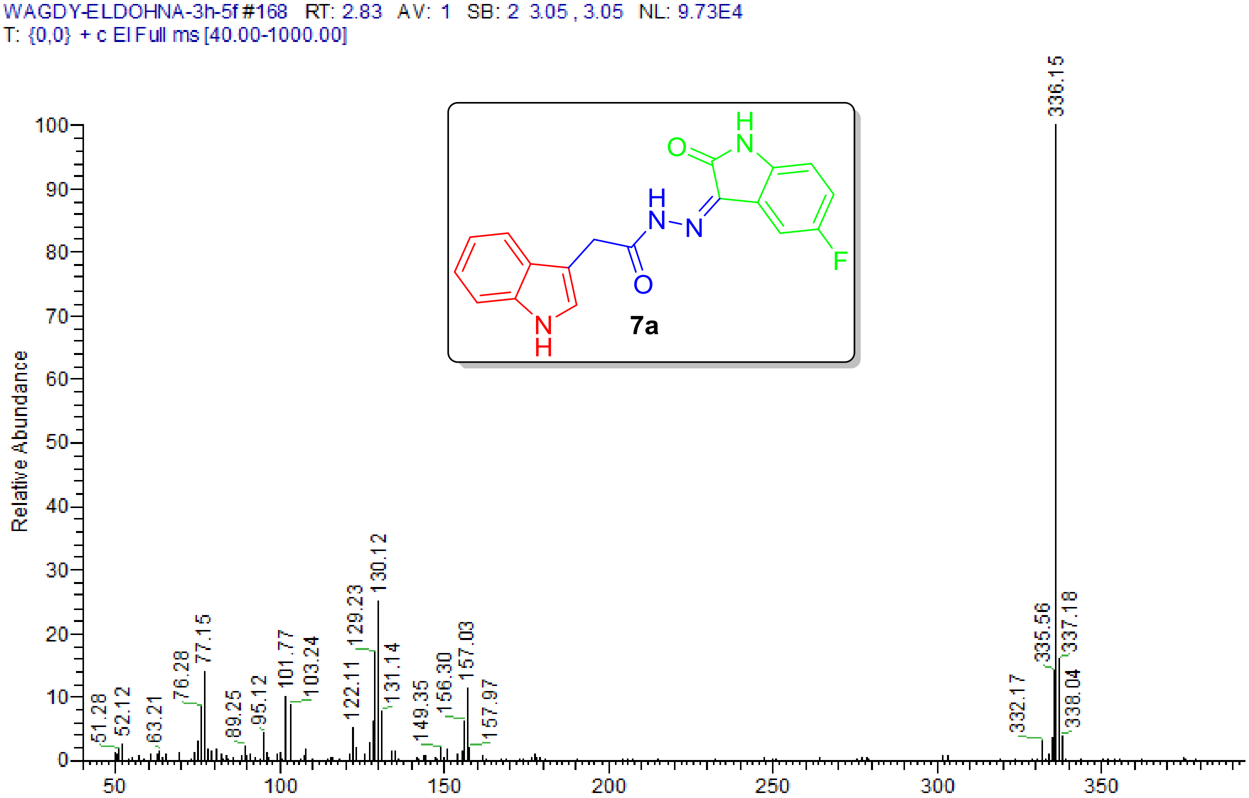
WAGDY-ELDOHNA-3h-5f #168 RT: 2.83 AV: 1 SB: 2 3.05 , 3.05 NL: 9.73E4
T: {0,0} + c EI Full ms [40.00-1000.00]
Relative Abundance
7a
336.15
130.12
129.23
77.15
76.28
101.77
103.24
157.03
156.30
157.97
149.35
131.14
122.11
95.12
89.25
63.21
52.12
51.28
335.56
337.18
338.04
332.17

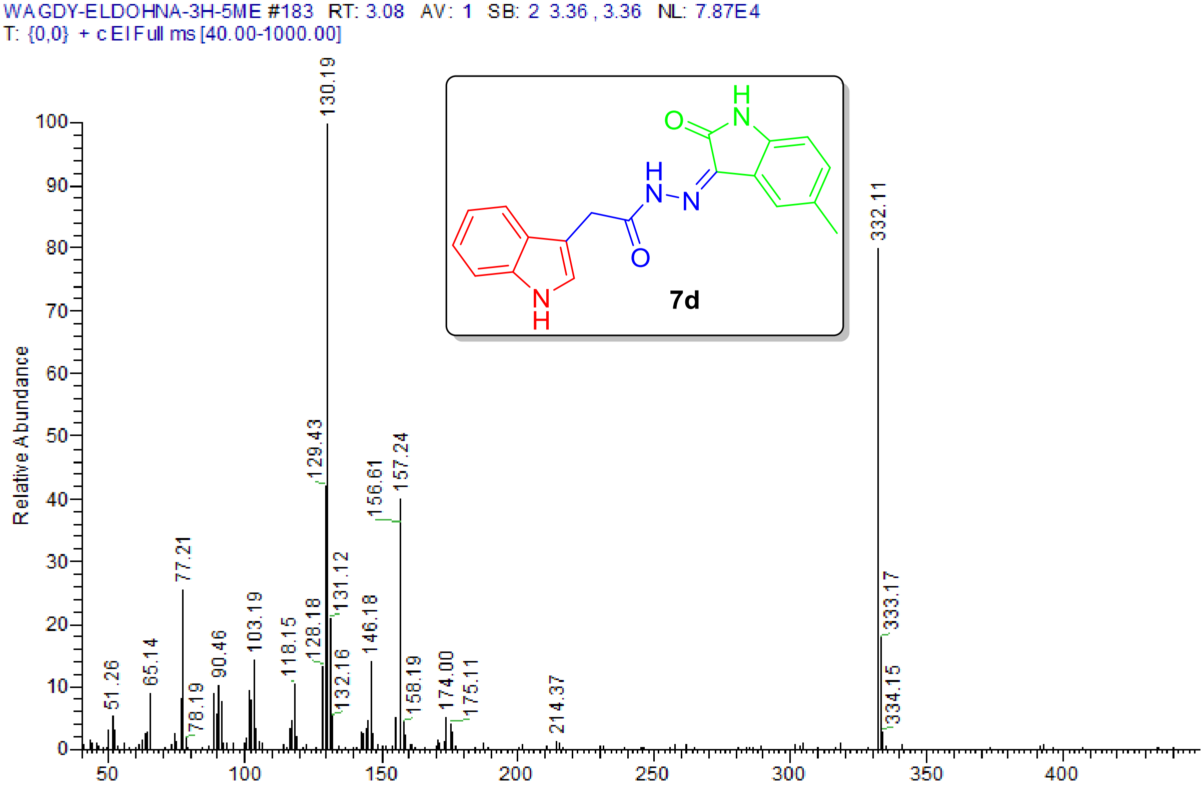
WAGDY-ELDOHNA-3H-5ME #183 RT: 3.08 AV: 1 SB: 2 3.36 , 3.36 NL: 7.87E4
T: {0,0} + c EI Full ms [40.00-1000.00]
Relative Abundance
7d
130.19
332.11
129.43
157.24
156.61
77.21
131.12
103.19
146.18
128.18
118.15
90.46
65.14
51.26
78.19
132.16
158.19
174.00
175.11
214.37
333.17
334.15

WAGDY-ELDOHNA-3h-isobuty #149 RT: 2.51 AV: 1 SB: 2 3.38 , 3.38 NL: 5.92E4
T: {0,0} + c EI Full ms [40.00-1000.00]

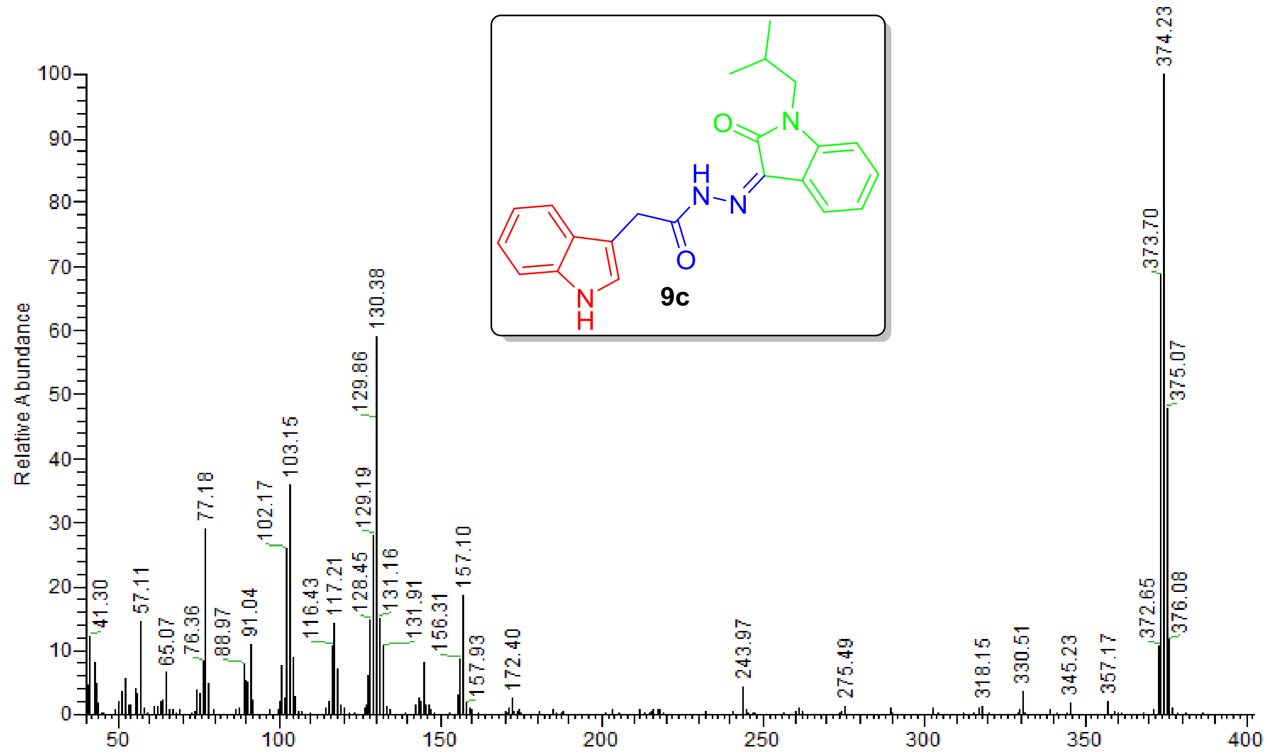

WAGDY-ELDOHNA-3H-NBZ #168 RT: 2.83 AV: 1 SB: 2 4.45 , 4.45 NL: 1.54E5
T: {0,0} + c EI Full ms [40.00-1000.00]

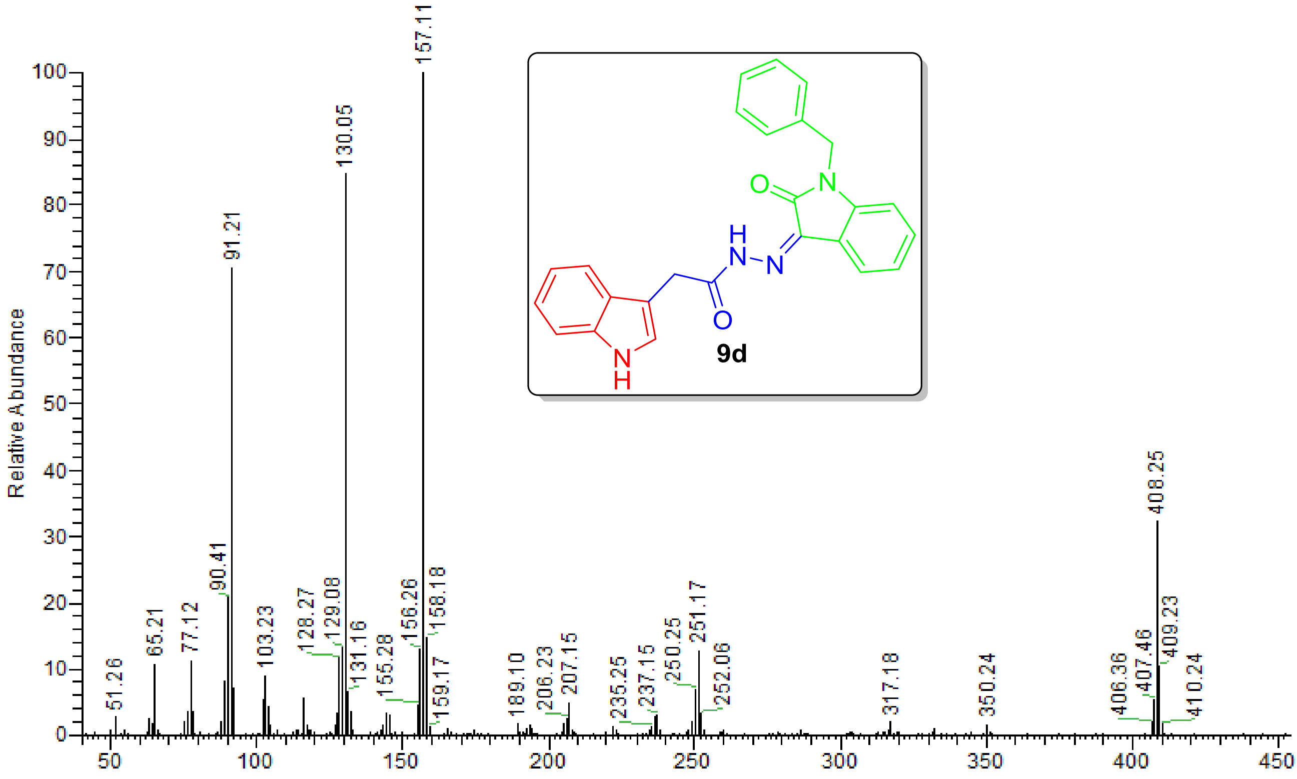

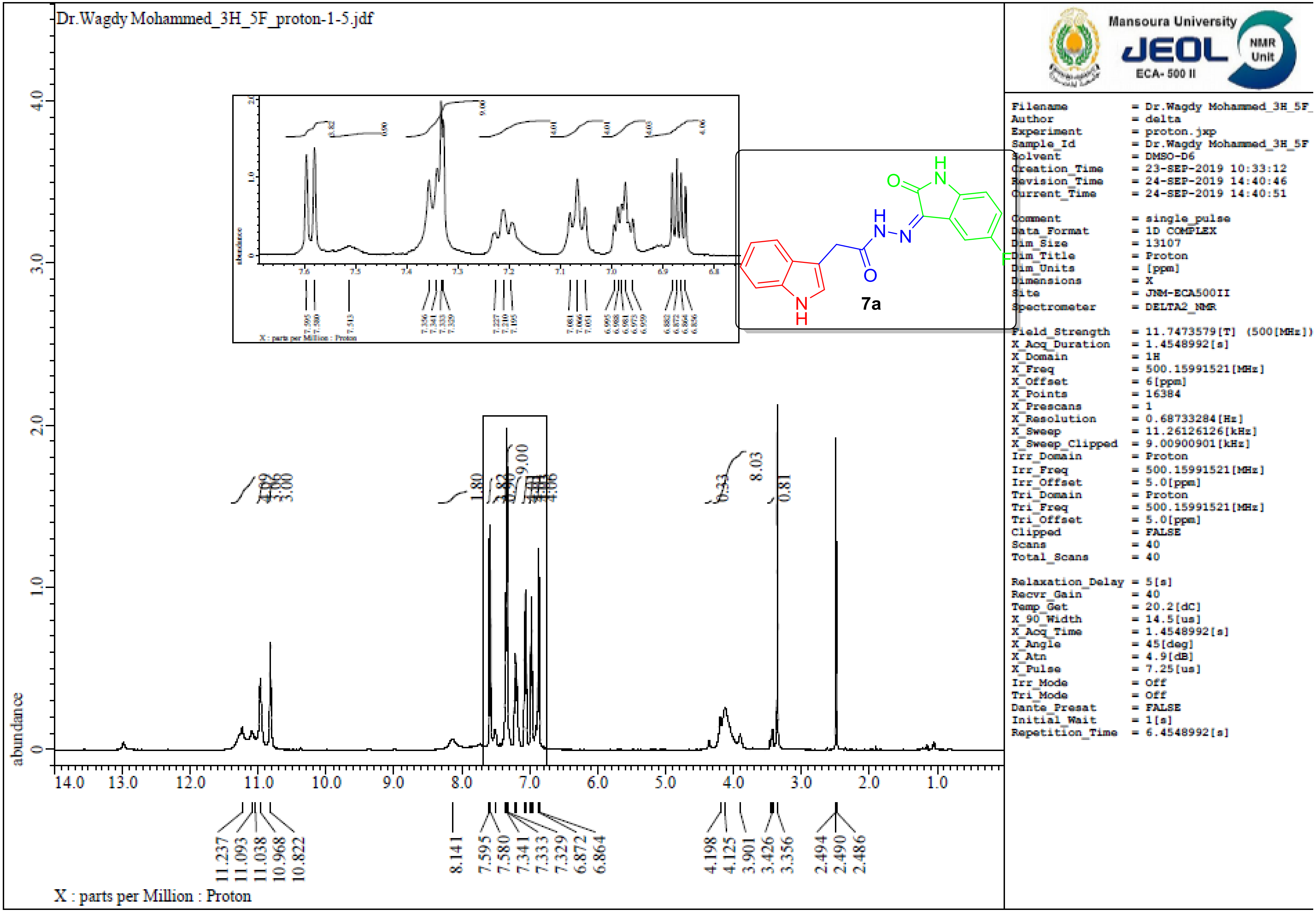

Dr.Wagdy Mohammed_3H_5F_proton-1-5.jdf
Mansoura University
JEOL
ECA- 500 II
NMR Unit
7a
X : parts per Million : Proton
abundance
11.237
11.093
11.038
10.968
10.822
8.141
7.595
7.580
7.341
7.333
7.329
6.872
6.864
4.198
4.125
3.901
3.426
3.356
2.494
2.490
2.486
Filename = Dr.Wagdy Mohammed_3H_5F_
Author = delta
Experiment = proton.jxp
Sample_Id = Dr.Wagdy Mohammed_3H_5F
Solvent = DMSO-D6
Creation_Time = 23-SEP-2019 10:33:12
Revision_Time = 24-SEP-2019 14:40:46
Current_Time = 24-SEP-2019 14:40:51
Comment = single_pulse
Data_Format = 1D COMPLEX
Dim_Size = 13107
Dim_Title = Proton
Dim_Units = [ppm]
Dimensions = X
Site = JNM-ECA500II
Spectrometer = DELTA2_NMR
Field_Strength = 11.7473579[T] (500[MHz])
X_Acq_Duration = 1.4548992[s]
X_Domain = 1H
X_Freq = 500.15991521[MHz]
X_Offset = 6[ppm]
X_Points = 16384
X_Prescans = 1
X_Resolution = 0.68733284[Hz]
X_Sweep = 11.26126126[kHz]
X_Sweep_Clipped = 9.00900901[kHz]
Irr_Domain = Proton
Irr_Freq = 500.15991521[MHz]
Irr_Offset = 5.0[ppm]
Tri_Domain = Proton
Tri_Freq = 500.15991521[MHz]
Tri_Offset = 5.0[ppm]
Clipped = FALSE
Scans = 40
Total_Scans = 40
Relaxation_Delay = 5[s]
Recvr_Gain = 40
Temp_Get = 20.2[dC]
X_90_Width = 14.5[us]
X_Acq_Time = 1.4548992[s]
X_Angle = 45[deg]
X_Atn = 4.9[dB]
X_Pulse = 7.25[us]
Irr_Mode = Off
Tri_Mode = Off
Dante_Presat = FALSE
Initial_Wait = 1[s]
Repetition_Time = 6.4548992[s]

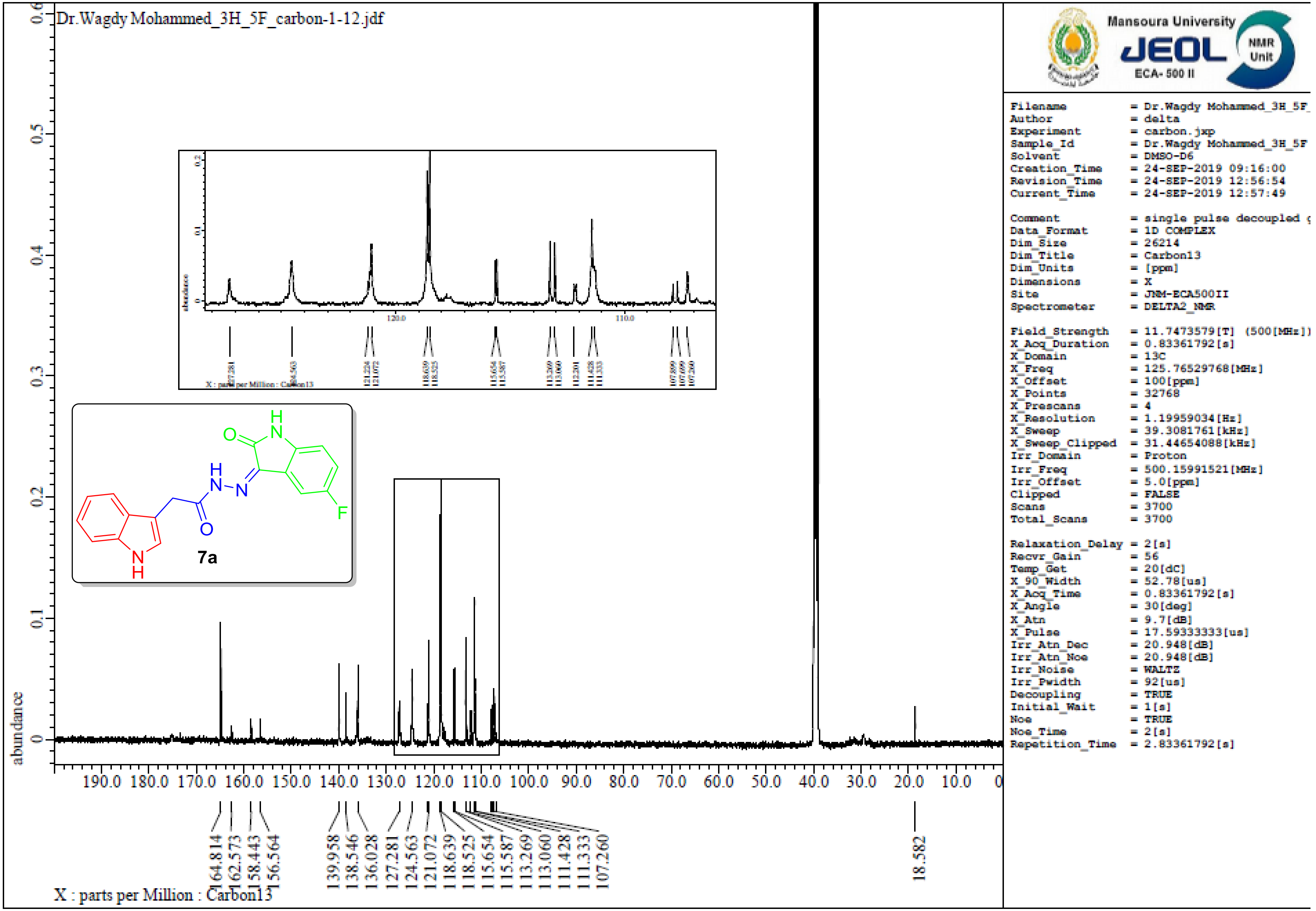

Dr.Wagdy Mohammed_3H_5F_carbon-1-12.jdf
Mansoura University
JEOL
NMR Unit
ECA- 500 II
Filename = Dr.Wagdy Mohammed_3H_5F_
Author = delta
Experiment = carbon.jxp
Sample_Id = Dr.Wagdy Mohammed_3H_5F
Solvent = DMSO-D6
Creation_Time = 24-SEP-2019 09:16:00
Revision_Time = 24-SEP-2019 12:56:54
Current_Time = 24-SEP-2019 12:57:49
Comment = single pulse decoupled g
Data_Format = 1D COMPLEX
Dim_Size = 26214
Dim_Title = Carbon13
Dim_Units = [ppm]
Dimensions = X
Site = JNM-ECA500II
Spectrometer = DELTA2_NMR
Field_Strength = 11.7473579[T] (500[MHz])
X_Acq_Duration = 0.83361792[s]
X_Domain = 13C
X_Freq = 125.76529768[MHz]
X_Offset = 100[ppm]
X_Points = 32768
X_Prescans = 4
X_Resolution = 1.19959034[Hz]
X_Sweep = 39.3081761[kHz]
X_Sweep_Clipped = 31.44654088[kHz]
Irr_Domain = Proton
Irr_Freq = 500.15991521[MHz]
Irr_Offset = 5.0[ppm]
Clipped = FALSE
Scans = 3700
Total_Scans = 3700
Relaxation_Delay = 2[s]
Recvr_Gain = 56
Temp_Get = 20[dC]
X_90_Width = 52.78[us]
X_Acq_Time = 0.83361792[s]
X_Angle = 30[deg]
X_Atn = 9.7[dB]
X_Pulse = 17.59333333[us]
Irr_Atn_Dec = 20.948[dB]
Irr_Atn_Noe = 20.948[dB]
Irr_Noise = WALTZ
Irr_Pwidth = 92[us]
Decoupling = TRUE
Initial_Wait = 1[s]
Noe = TRUE
Noe_Time = 2[s]
Repetition_Time = 2.83361792[s]
7a
abundance
X : parts per Million : Carbon13
164.814
162.573
158.443
156.564
139.958
138.546
136.028
127.281
124.563
121.072
118.639
118.525
115.654
115.587
113.269
113.060
111.428
111.333
107.260
18.582

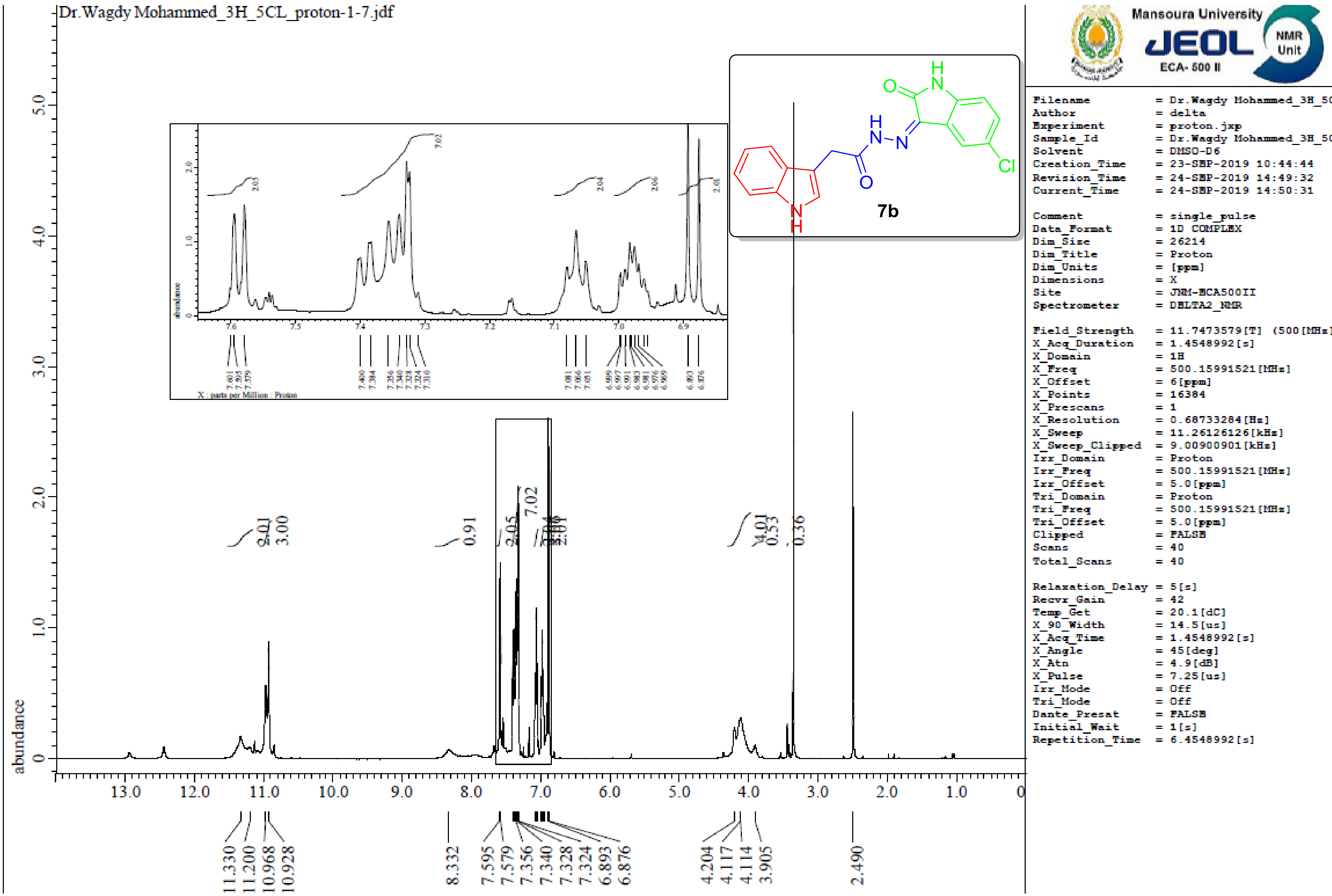
Dr.Wagdy Mohammed_3H_5CL_proton-1-7.jdf
Mansoura University
JEOL
NMR Unit
ECA- 500 II
7b
Filename = Dr.Wagdy Mohammed_3H_5C
Author = delta
Experiment = proton.jxp
Sample_Id = Dr.Wagdy Mohammed_3H_5C
Solvent = DMSO-D6
Creation_Time = 23-SEP-2019 10:44:44
Revision_Time = 24-SEP-2019 14:49:32
Current_Time = 24-SEP-2019 14:50:31
Comment = single_pulse
Data_Format = 1D COMPLEX
Dim_Size = 26214
Dim_Title = Proton
Dim_Units = [ppm]
Dimensions = X
Site = JNM-ECA500II
Spectrometer = DELTA2_NMR
Field_Strength = 11.7473579[T] (500[MHz
X_Acq_Duration = 1.4548992[s]
X_Domain = 1H
X_Freq = 500.15991521[MHz]
X_Offset = 6[ppm]
X_Points = 16384
X_Prescans = 1
X_Resolution = 0.68733284[Hz]
X_Sweep = 11.26126126[kHz]
X_Sweep_Clipped = 9.00900901[kHz]
Irr_Domain = Proton
Irr_Freq = 500.15991521[MHz]
Irr_Offset = 5.0[ppm]
Tri_Domain = Proton
Tri_Freq = 500.15991521[MHz]
Tri_Offset = 5.0[ppm]
Clipped = FALSE
Scans = 40
Total_Scans = 40
Relaxation_Delay = 5[s]
Recvr_Gain = 42
Temp_Get = 20.1[dC]
X_90_Width = 14.5[us]
X_Acq_Time = 1.4548992[s]
X_Angle = 45[deg]
X_Atn = 4.9[dB]
X_Pulse = 7.25[us]
Irr_Mode = Off
Tri_Mode = Off
Dante_Presat = FALSE
Initial_Wait = 1[s]
Repetition_Time = 6.4548992[s]
abundance
X : parts per Million : Proton
11.330 11.200 10.968 10.928 8.332 7.595 7.579 7.356 7.340 7.328 7.324 6.893 6.876 4.204 4.117 4.114 3.905 2.490

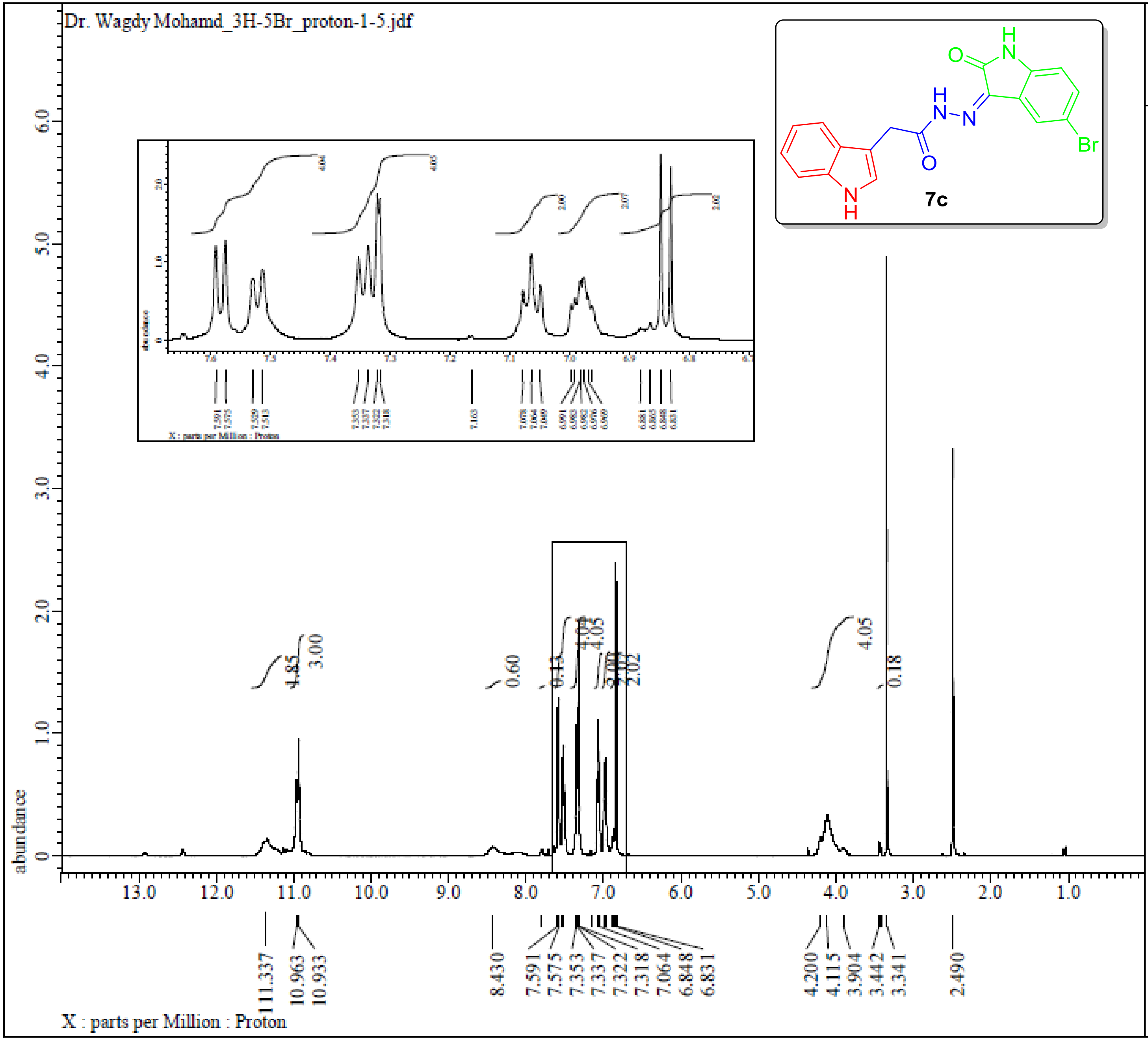

Mansoura University
JEOL ECA- 500 II
NMR Unit

Filename	=	Dr. Wagdy Mohamd_3H-5Br_
Author	=	delta
Experiment	=	proton.jxp
Sample_Id	=	Dr. Wagdy Mohamd_3H-5Br
Solvent	=	DMSO-D6
Creation_Time	=	17-SEP-2019 15:44:08
Revision_Time	=	17-SEP-2019 15:50:12
Current_Time	=	17-SEP-2019 15:50:40
Comment	=	single_pulse
Data_Format	=	1D COMPLEX
Dim_Size	=	26214
Dim_Title	=	Proton
Dim_Units	=	[ppm]
Dimensions	=	X
Site	=	JNM-ECA500II
Spectrometer	=	DELTA2_NMR
Field_Strength	=	11.7473579[T] (500[MHz])
X_Acq_Duration	=	1.4548992[s]
X_Domain	=	1H
X_Freq	=	500.15991521[MHz]
X_Offset	=	6[ppm]
X_Points	=	16384
X_Prescans	=	1
X_Resolution	=	0.68733284[Hz]
X_Sweep	=	11.26126126[kHz]
X_Sweep_Clipped	=	9.00900901[kHz]
Irr_Domain	=	Proton
Irr_Freq	=	500.15991521[MHz]
Irr_Offset	=	5.0[ppm]
Tri_Domain	=	Proton
Tri_Freq	=	500.15991521[MHz]
Tri_Offset	=	5.0[ppm]
Clipped	=	FALSE
Scans	=	40
Total_Scans	=	40
Relaxation_Delay	=	5[s]
Recvr_Gain	=	46
Temp_Get	=	19.9[dC]
X_90_Width	=	14.5[us]
X_Acq_Time	=	1.4548992[s]
X_Angle	=	45[deg]
X_Atn	=	4.9[dB]
X_Pulse	=	7.25[us]
Irr_Mode	=	Off
Tri_Mode	=	Off
Dante_Presat	=	FALSE
Initial_Wait	=	1[s]
Repetition_Time	=	6.4548992[s]

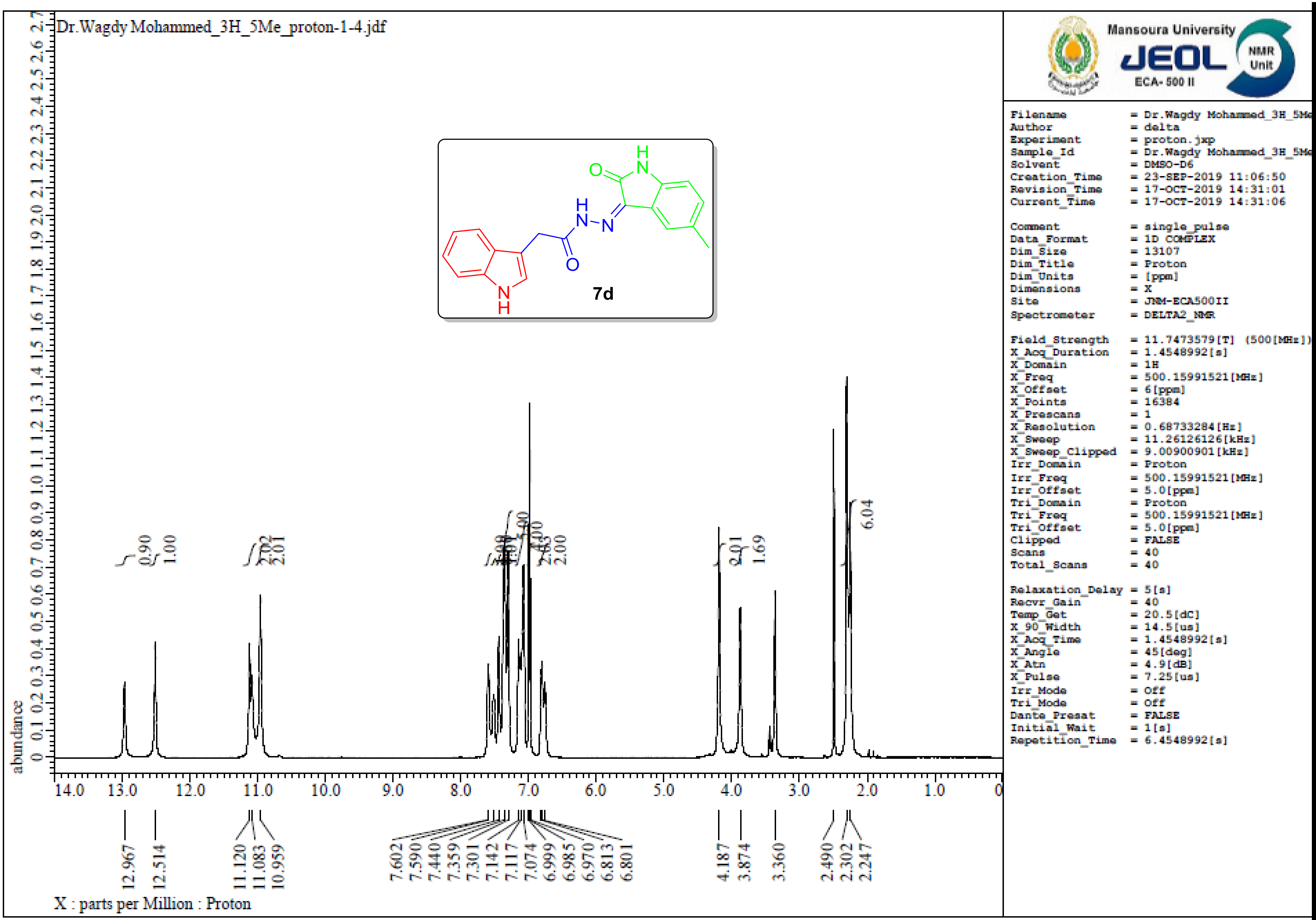
Dr.Wagdy Mohammed_3H_5Me_proton-1-4.jdf
Mansoura University
JEOL
NMR Unit
ECA- 500 II
7d
abundance
X : parts per Million : Proton
12.967
12.514
11.120
11.083
10.959
7.602
7.590
7.440
7.359
7.301
7.142
7.117
7.074
6.999
6.985
6.970
6.813
6.801
4.187
3.874
3.360
2.490
2.302
2.247
0.90
1.00
2.01
6.04
1.69
2.00
Filename = Dr.Wagdy Mohammed_3H_5Me
Author = delta
Experiment = proton.jxp
Sample_Id = Dr.Wagdy Mohammed_3H_5Me
Solvent = DMSO-D6
Creation_Time = 23-SEP-2019 11:06:50
Revision_Time = 17-OCT-2019 14:31:01
Current_Time = 17-OCT-2019 14:31:06
Comment = single_pulse
Data_Format = 1D COMPLEX
Dim_Size = 13107
Dim_Title = Proton
Dim_Units = [ppm]
Dimensions = X
Site = JNM-ECA500II
Spectrometer = DELTA2_NMR
Field_Strength = 11.7473579[T] (500[MHz])
X_Acq_Duration = 1.4548992[s]
X_Domain = 1H
X_Freq = 500.15991521[MHz]
X_Offset = 6[ppm]
X_Points = 16384
X_Prescans = 1
X_Resolution = 0.68733284[Hz]
X_Sweep = 11.26126126[kHz]
X_Sweep_Clipped = 9.00900901[kHz]
Irr_Domain = Proton
Irr_Freq = 500.15991521[MHz]
Irr_Offset = 5.0[ppm]
Tri_Domain = Proton
Tri_Freq = 500.15991521[MHz]
Tri_Offset = 5.0[ppm]
Clipped = FALSE
Scans = 40
Total_Scans = 40
Relaxation_Delay = 5[s]
Recvr_Gain = 40
Temp_Get = 20.5[dC]
X_90_Width = 14.5[us]
X_Acq_Time = 1.4548992[s]
X_Angle = 45[deg]
X_Atn = 4.9[dB]
X_Pulse = 7.25[us]
Irr_Mode = Off
Tri_Mode = Off
Dante_Presat = FALSE
Initial_Wait = 1[s]
Repetition_Time = 6.4548992[s]

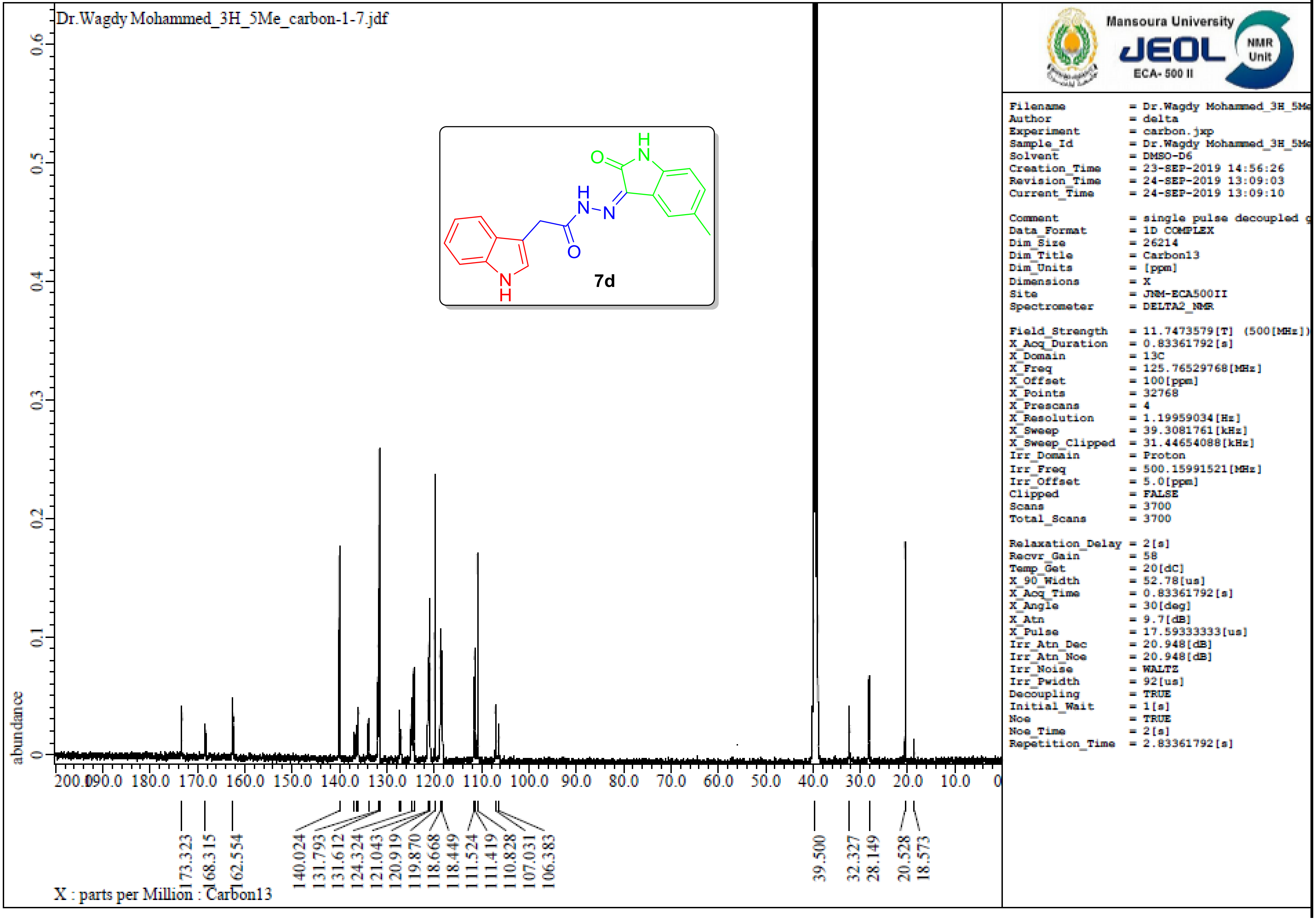

Dr.Wagdy Mohammed_3H_5Me_carbon-1-7.jdf
7d
abundance
X : parts per Million : Carbon13
173.323
168.315
162.554
140.024
131.793
131.612
124.324
121.043
120.919
119.870
118.668
118.449
111.524
111.419
110.828
107.031
106.383
39.500
32.327
28.149
20.528
18.573
Mansoura University
JEOL
ECA- 500 II
NMR Unit
Filename = Dr.Wagdy Mohammed_3H_5Me
Author = delta
Experiment = carbon.jxp
Sample_Id = Dr.Wagdy Mohammed_3H_5Me
Solvent = DMSO-D6
Creation_Time = 23-SEP-2019 14:56:26
Revision_Time = 24-SEP-2019 13:09:03
Current_Time = 24-SEP-2019 13:09:10
Comment = single pulse decoupled g
Data_Format = 1D COMPLEX
Dim_Size = 26214
Dim_Title = Carbon13
Dim_Units = [ppm]
Dimensions = X
Site = JNM-ECA500II
Spectrometer = DELTA2_NMR
Field_Strength = 11.7473579[T] (500[MHz])
X_Acq_Duration = 0.83361792[s]
X_Domain = 13C
X_Freq = 125.76529768[MHz]
X_Offset = 100[ppm]
X_Points = 32768
X_Prescans = 4
X_Resolution = 1.19959034[Hz]
X_Sweep = 39.3081761[kHz]
X_Sweep_Clipped = 31.44654088[kHz]
Irr_Domain = Proton
Irr_Freq = 500.15991521[MHz]
Irr_Offset = 5.0[ppm]
Clipped = FALSE
Scans = 3700
Total_Scans = 3700
Relaxation_Delay = 2[s]
Recvr_Gain = 58
Temp_Get = 20[dC]
X_90_Width = 52.78[us]
X_Acq_Time = 0.83361792[s]
X_Angle = 30[deg]
X_Atn = 9.7[dB]
X_Pulse = 17.59333333[us]
Irr_Atn_Dec = 20.948[dB]
Irr_Atn_Noe = 20.948[dB]
Irr_Noise = WALTZ
Irr_Pwidth = 92[us]
Decoupling = TRUE
Initial_Wait = 1[s]
Noe = TRUE
Noe_Time = 2[s]
Repetition_Time = 2.83361792[s]

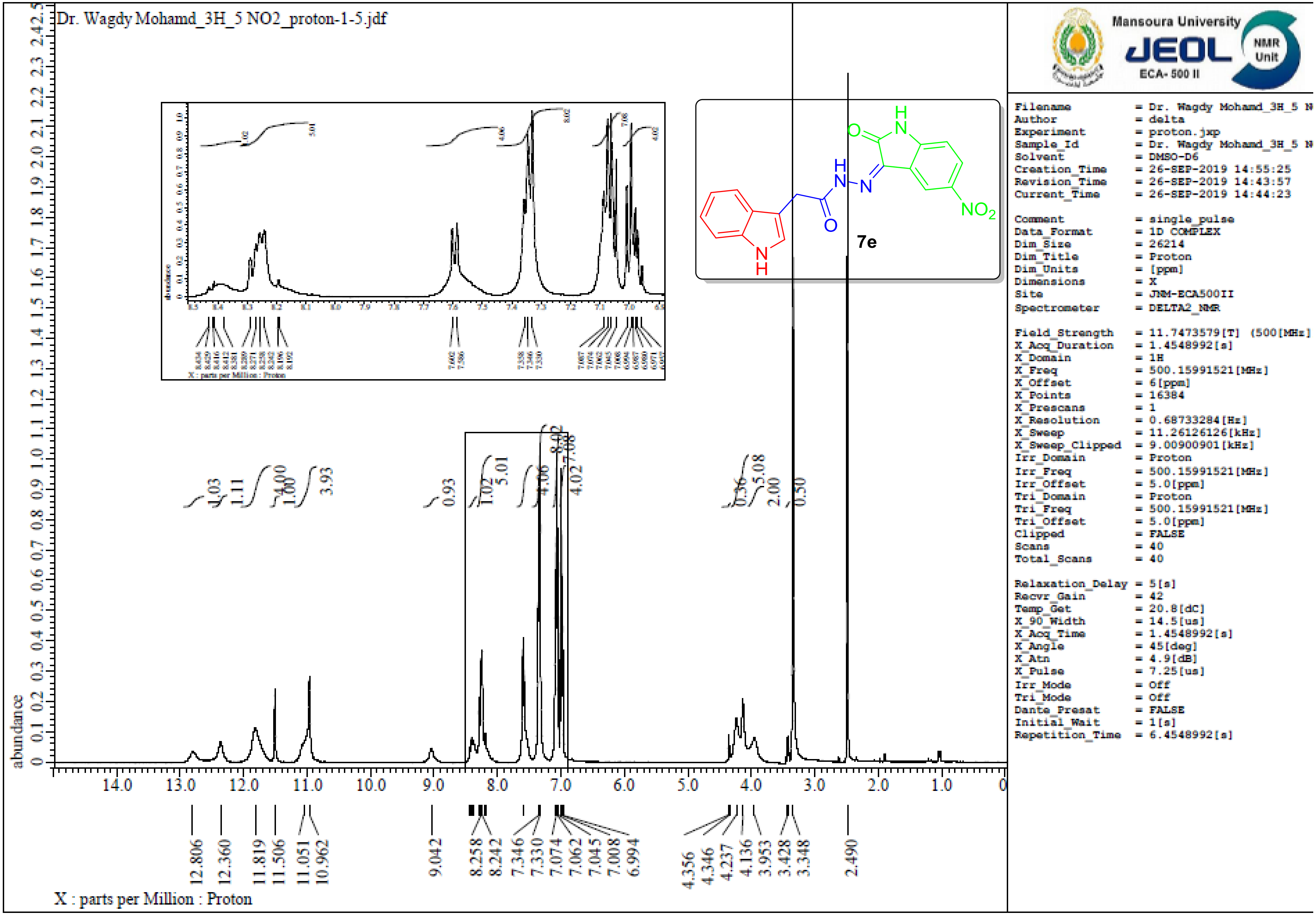
Dr. Wagdy Mohamd_3H_5 NO2_proton-1-5.jdf
Mansoura University
JEOL
NMR Unit
ECA-500 II
7e
NO2
X : parts per Million : Proton
abundance
12.806
12.360
11.819
11.506
11.051
10.962
9.042
8.258
8.242
7.346
7.330
7.074
7.062
7.045
7.008
6.994
4.356
4.346
4.237
4.136
3.953
3.428
3.348
2.490
Filename = Dr. Wagdy Mohamd_3H_5 N
Author = delta
Experiment = proton.jxp
Sample_Id = Dr. Wagdy Mohamd_3H_5 N
Solvent = DMSO-D6
Creation_Time = 26-SEP-2019 14:55:25
Revision_Time = 26-SEP-2019 14:43:57
Current_Time = 26-SEP-2019 14:44:23
Comment = single_pulse
Data_Format = 1D COMPLEX
Dim_Size = 26214
Dim_Title = Proton
Dim_Units = [ppm]
Dimensions = X
Site = JNM-ECA500II
Spectrometer = DELTA2_NMR
Field_Strength = 11.7473579[T] (500[MHz]
X_Acq_Duration = 1.4548992[s]
X_Domain = 1H
X_Freq = 500.15991521[MHz]
X_Offset = 6[ppm]
X_Points = 16384
X_Prescans = 1
X_Resolution = 0.68733284[Hz]
X_Sweep = 11.26126126[kHz]
X_Sweep_Clipped = 9.00900901[kHz]
Irr_Domain = Proton
Irr_Freq = 500.15991521[MHz]
Irr_Offset = 5.0[ppm]
Tri_Domain = Proton
Tri_Freq = 500.15991521[MHz]
Tri_Offset = 5.0[ppm]
Clipped = FALSE
Scans = 40
Total_Scans = 40
Relaxation_Delay = 5[s]
Recvr_Gain = 42
Temp_Get = 20.8[dC]
X_90_Width = 14.5[us]
X_Acq_Time = 1.4548992[s]
X_Angle = 45[deg]
X_Atn = 4.9[dB]
X_Pulse = 7.25[us]
Irr_Mode = Off
Tri_Mode = Off
Dante_Presat = FALSE
Initial_Wait = 1[s]
Repetition_Time = 6.4548992[s]

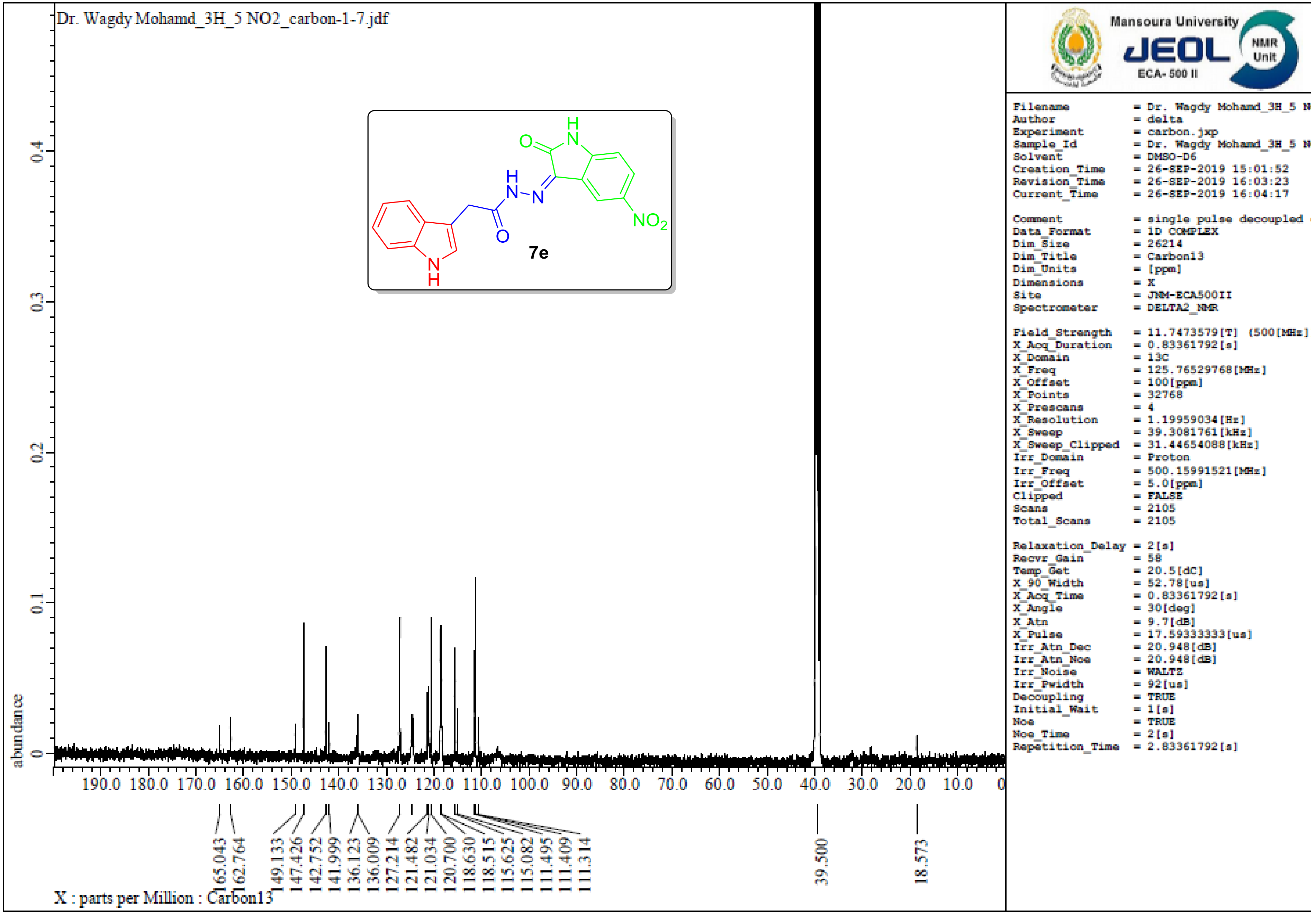

Dr. Wagdy Mohamd_3H_5 NO2_carbon-1-7.jdf
7e
NO2
abundance
X : parts per Million : Carbon13
165.043
162.764
149.133
147.426
142.752
141.999
136.123
136.009
127.214
121.482
121.034
120.700
118.630
118.515
115.625
115.082
111.495
111.409
111.314
39.500
18.573
Mansoura University
JEOL
NMR Unit
ECA- 500 II
Filename = Dr. Wagdy Mohamd_3H_5
Author = delta
Experiment = carbon.jxp
Sample_Id = Dr. Wagdy Mohamd_3H_5
Solvent = DMSO-D6
Creation_Time = 26-SEP-2019 15:01:52
Revision_Time = 26-SEP-2019 16:03:23
Current_Time = 26-SEP-2019 16:04:17
Comment = single pulse decoupled
Data_Format = 1D COMPLEX
Dim_Size = 26214
Dim_Title = Carbon13
Dim_Units = [ppm]
Dimensions = X
Site = JNM-ECA500II
Spectrometer = DELTA2_NMR
Field_Strength = 11.7473579[T] (500[MHz]
X_Acq_Duration = 0.83361792[s]
X_Domain = 13C
X_Freq = 125.76529768[MHz]
X_Offset = 100[ppm]
X_Points = 32768
X_Prescans = 4
X_Resolution = 1.19959034[Hz]
X_Sweep = 39.3081761[kHz]
X_Sweep_Clipped = 31.44654088[kHz]
Irr_Domain = Proton
Irr_Freq = 500.15991521[MHz]
Irr_Offset = 5.0[ppm]
Clipped = FALSE
Scans = 2105
Total_Scans = 2105
Relaxation_Delay = 2[s]
Recvr_Gain = 58
Temp_Get = 20.5[dC]
X_90_Width = 52.78[us]
X_Acq_Time = 0.83361792[s]
X_Angle = 30[deg]
X_Atn = 9.7[dB]
X_Pulse = 17.59333333[us]
Irr_Atn_Dec = 20.948[dB]
Irr_Atn_Noe = 20.948[dB]
Irr_Noise = WALTZ
Irr_Pwidth = 92[us]
Decoupling = TRUE
Initial_Wait = 1[s]
Noe = TRUE
Noe_Time = 2[s]
Repetition_Time = 2.83361792[s]

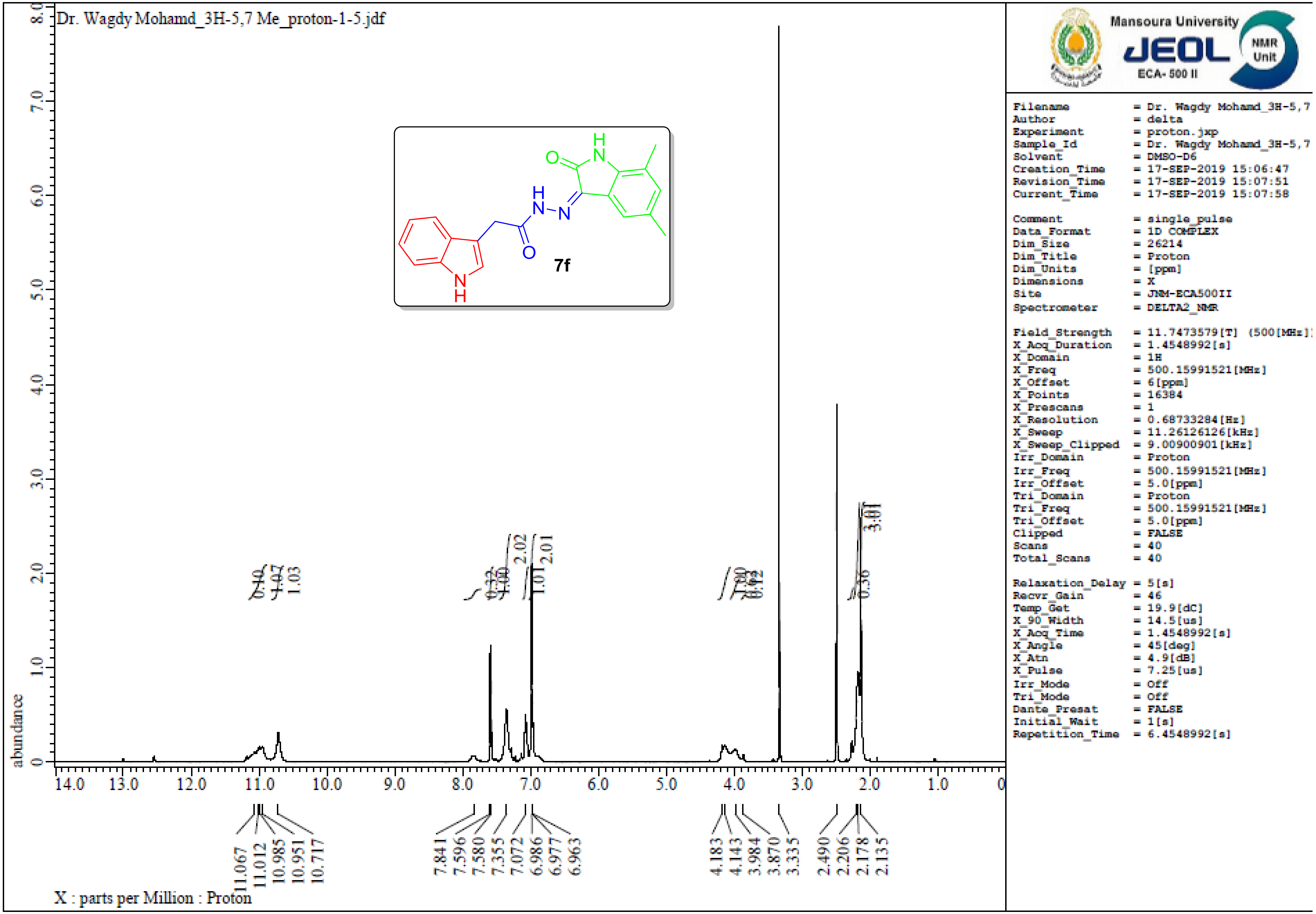
Dr. Wagdy Mohamd_3H-5,7 Me_proton-1-5.jdf
7f
Mansoura University
JEOL
ECA- 500 II
NMR Unit
Filename = Dr. Wagdy Mohamd_3H-5,7
Author = delta
Experiment = proton.jxp
Sample_Id = Dr. Wagdy Mohamd_3H-5,7
Solvent = DMSO-D6
Creation_Time = 17-SEP-2019 15:06:47
Revision_Time = 17-SEP-2019 15:07:51
Current_Time = 17-SEP-2019 15:07:58
Comment = single_pulse
Data_Format = 1D COMPLEX
Dim_Size = 26214
Dim_Title = Proton
Dim_Units = [ppm]
Dimensions = X
Site = JNM-ECA500II
Spectrometer = DELTA2_NMR
Field_Strength = 11.7473579[T] (500[MHz])
X_Acq_Duration = 1.4548992[s]
X_Domain = 1H
X_Freq = 500.15991521[MHz]
X_Offset = 6[ppm]
X_Points = 16384
X_Prescans = 1
X_Resolution = 0.68733284[Hz]
X_Sweep = 11.26126126[kHz]
X_Sweep_Clipped = 9.00900901[kHz]
Irr_Domain = Proton
Irr_Freq = 500.15991521[MHz]
Irr_Offset = 5.0[ppm]
Tri_Domain = Proton
Tri_Freq = 500.15991521[MHz]
Tri_Offset = 5.0[ppm]
Clipped = FALSE
Scans = 40
Total_Scans = 40
Relaxation_Delay = 5[s]
Recvr_Gain = 46
Temp_Get = 19.9[dC]
X_90_Width = 14.5[us]
X_Acq_Time = 1.4548992[s]
X_Angle = 45[deg]
X_Atn = 4.9[dB]
X_Pulse = 7.25[us]
Irr_Mode = Off
Tri_Mode = Off
Dante_Presat = FALSE
Initial_Wait = 1[s]
Repetition_Time = 6.4548992[s]
abundance
X : parts per Million : Proton
11.067 11.012 10.985 10.951 10.717
7.841 7.596 7.580 7.355 7.072 6.986 6.977 6.963
4.183 4.143 3.984 3.870 3.335
2.490 2.206 2.178 2.135
1.03 2.02 2.01 3.01

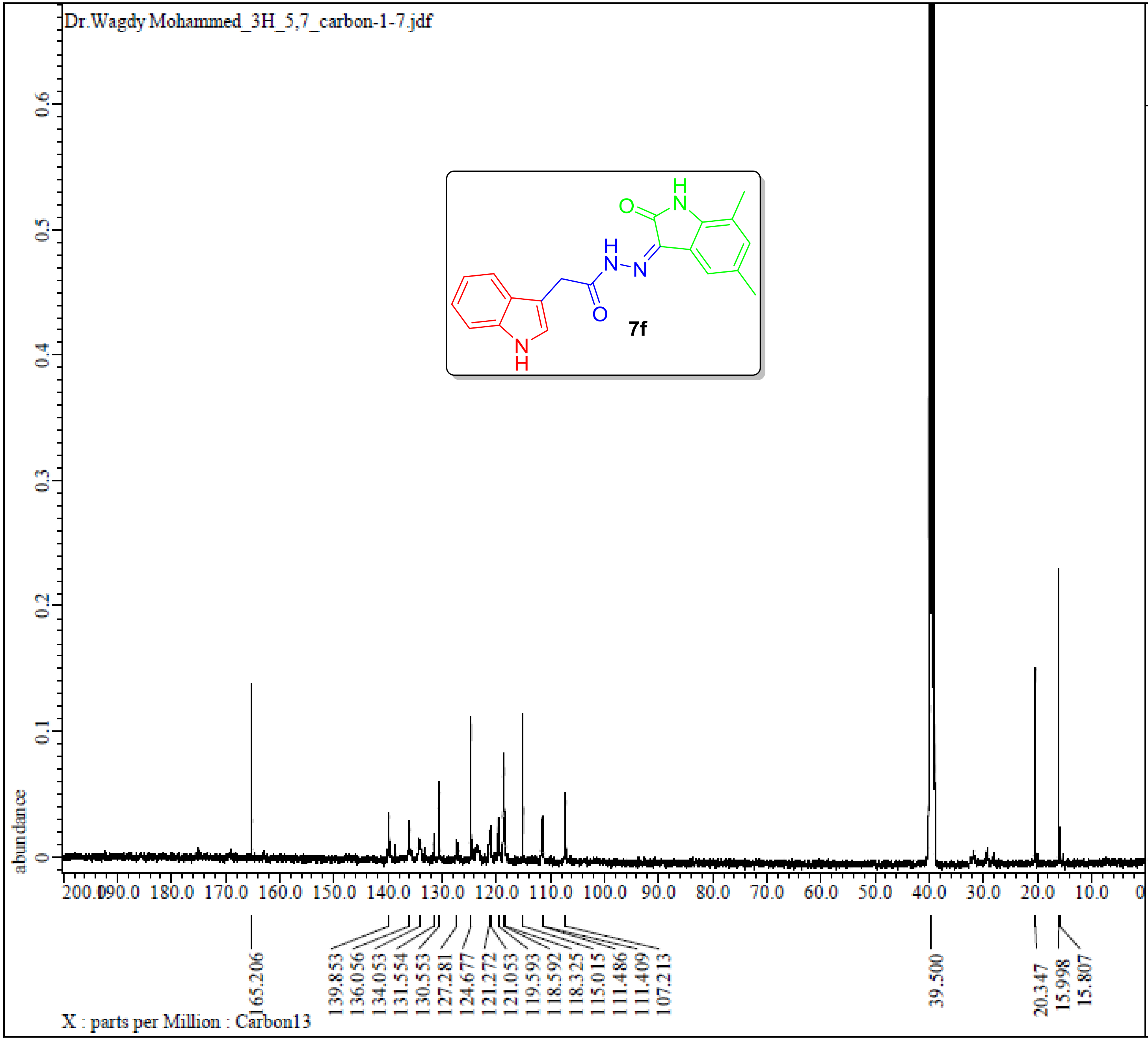

Mansoura University
JEOL
ECA- 500 II
NMR Unit

```
Filename            = Dr.Wagdy Mohammed_3H_5,7
Author              = delta
Experiment          = carbon.jxp
Sample_Id           = Dr.Wagdy Mohammed_3H_5,7
Solvent             = DMSO-D6
Creation_Time       = 24-SEP-2019 13:28:13
Revision_Time       = 24-SEP-2019 15:44:03
Current_Time        = 24-SEP-2019 15:44:11

Comment             = single pulse decoupled g
Data_Format         = 1D COMPLEX
Dim_Size            = 26214
Dim_Title           = Carbon13
Dim_Units           = [ppm]
Dimensions          = X
Site                = JNM-ECA500II
Spectrometer        = DELTA2_NMR

Field_Strength      = 11.7473579[T] (500[MHz])
X_Acq_Duration      = 0.83361792[s]
X_Domain            = 13C
X_Freq              = 125.76529768[MHz]
X_Offset            = 100[ppm]
X_Points            = 32768
X_Prescans          = 4
X_Resolution        = 1.19959034[Hz]
X_Sweep             = 39.3081761[kHz]
X_Sweep_Clipped     = 31.44654088[kHz]
Irr_Domain          = Proton
Irr_Freq            = 500.15991521[MHz]
Irr_Offset          = 5.0[ppm]
Clipped             = FALSE
Scans               = 3300
Total_Scans         = 3300

Relaxation_Delay    = 2[s]
Recvr_Gain          = 58
Temp_Get            = 19.8[dC]
X_90_Width          = 52.78[us]
X_Acq_Time          = 0.83361792[s]
X_Angle             = 30[deg]
X_Atn               = 9.7[dB]
X_Pulse             = 17.59333333[us]
Irr_Atn_Dec         = 20.948[dB]
Irr_Atn_Noe         = 20.948[dB]
Irr_Noise           = WALTZ
Irr_Pwidth          = 92[us]
Decoupling          = TRUE
Initial_Wait        = 1[s]
Noe                 = TRUE
Noe_Time            = 2[s]
Repetition_Time     = 2.83361792[s]
```

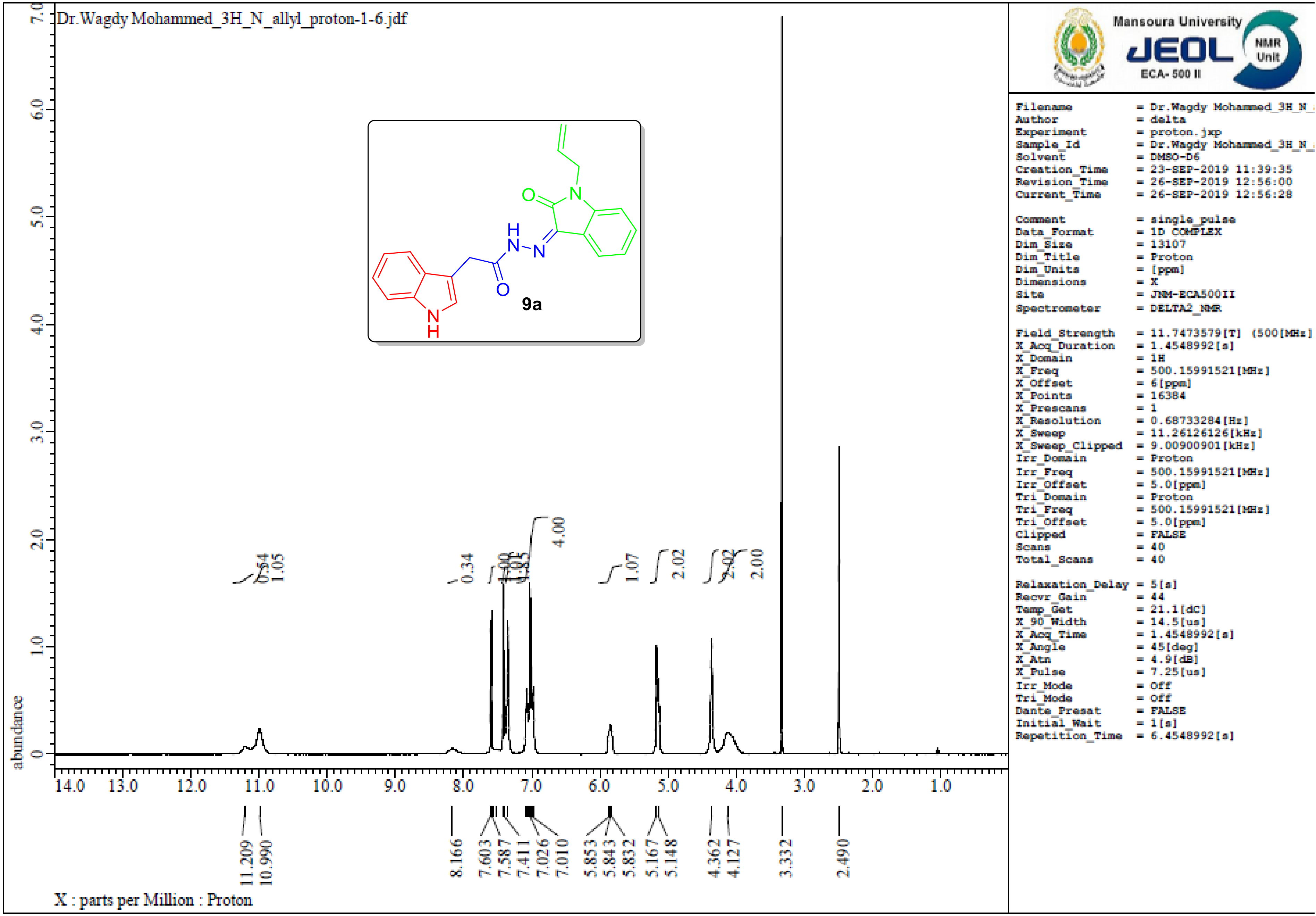
Dr.Wagdy Mohammed_3H_N_allyl_proton-1-6.jdf
9a
abundance
X : parts per Million : Proton
11.209
10.990
8.166
7.603
7.587
7.411
7.026
7.010
5.853
5.843
5.832
5.167
5.148
4.362
4.127
3.332
2.490
0.54
1.05
0.34
1.00
1.05
4.00
1.07
2.02
2.02
2.00
Mansoura University
JEOL
ECA- 500 II
NMR Unit
Filename = Dr.Wagdy Mohammed_3H_N_
Author = delta
Experiment = proton.jxp
Sample_Id = Dr.Wagdy Mohammed_3H_N_
Solvent = DMSO-D6
Creation_Time = 23-SEP-2019 11:39:35
Revision_Time = 26-SEP-2019 12:56:00
Current_Time = 26-SEP-2019 12:56:28
Comment = single_pulse
Data_Format = 1D COMPLEX
Dim_Size = 13107
Dim_Title = Proton
Dim_Units = [ppm]
Dimensions = X
Site = JNM-ECA500II
Spectrometer = DELTA2_NMR
Field_Strength = 11.7473579[T] (500[MHz]
X_Acq_Duration = 1.4548992[s]
X_Domain = 1H
X_Freq = 500.15991521[MHz]
X_Offset = 6[ppm]
X_Points = 16384
X_Prescans = 1
X_Resolution = 0.68733284[Hz]
X_Sweep = 11.26126126[kHz]
X_Sweep_Clipped = 9.00900901[kHz]
Irr_Domain = Proton
Irr_Freq = 500.15991521[MHz]
Irr_Offset = 5.0[ppm]
Tri_Domain = Proton
Tri_Freq = 500.15991521[MHz]
Tri_Offset = 5.0[ppm]
Clipped = FALSE
Scans = 40
Total_Scans = 40
Relaxation_Delay = 5[s]
Recvr_Gain = 44
Temp_Get = 21.1[dC]
X_90_Width = 14.5[us]
X_Acq_Time = 1.4548992[s]
X_Angle = 45[deg]
X_Atn = 4.9[dB]
X_Pulse = 7.25[us]
Irr_Mode = Off
Tri_Mode = Off
Dante_Presat = FALSE
Initial_Wait = 1[s]
Repetition_Time = 6.4548992[s]

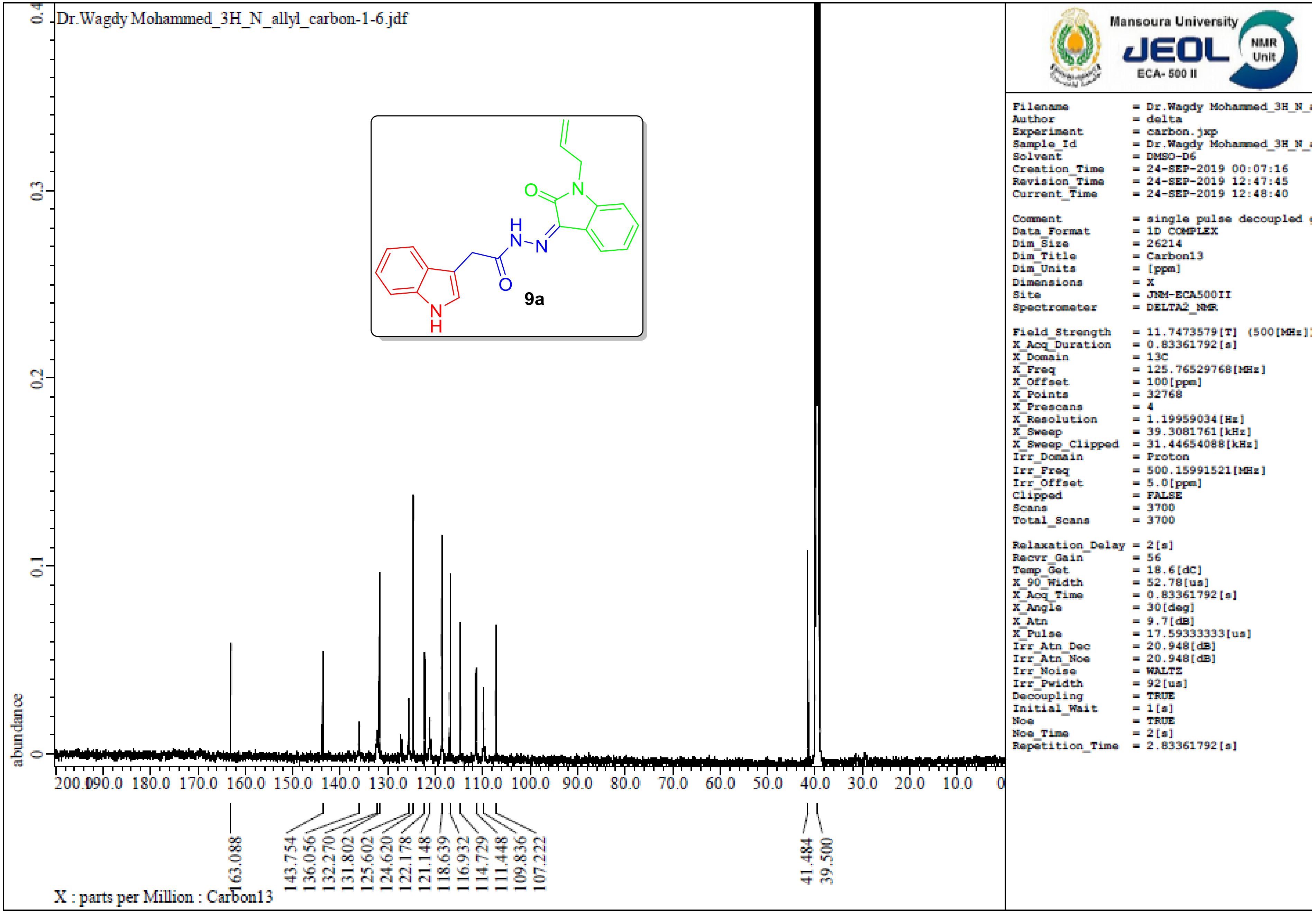

Dr.Wagdy Mohammed_3H_N_allyl_carbon-1-6.jdf
9a
Mansoura University
JEOL
NMR Unit
ECA- 500 II
Filename = Dr.Wagdy Mohammed_3H_N_
Author = delta
Experiment = carbon.jxp
Sample_Id = Dr.Wagdy Mohammed_3H_N_
Solvent = DMSO-D6
Creation_Time = 24-SEP-2019 00:07:16
Revision_Time = 24-SEP-2019 12:47:45
Current_Time = 24-SEP-2019 12:48:40
Comment = single pulse decoupled
Data_Format = 1D COMPLEX
Dim_Size = 26214
Dim_Title = Carbon13
Dim_Units = [ppm]
Dimensions = X
Site = JNM-ECA500II
Spectrometer = DELTA2_NMR
Field_Strength = 11.7473579[T] (500[MHz])
X_Acq_Duration = 0.83361792[s]
X_Domain = 13C
X_Freq = 125.76529768[MHz]
X_Offset = 100[ppm]
X_Points = 32768
X_Prescans = 4
X_Resolution = 1.19959034[Hz]
X_Sweep = 39.3081761[kHz]
X_Sweep_Clipped = 31.44654088[kHz]
Irr_Domain = Proton
Irr_Freq = 500.15991521[MHz]
Irr_Offset = 5.0[ppm]
Clipped = FALSE
Scans = 3700
Total_Scans = 3700
Relaxation_Delay = 2[s]
Recvr_Gain = 56
Temp_Get = 18.6[dC]
X_90_Width = 52.78[us]
X_Acq_Time = 0.83361792[s]
X_Angle = 30[deg]
X_Atn = 9.7[dB]
X_Pulse = 17.59333333[us]
Irr_Atn_Dec = 20.948[dB]
Irr_Atn_Noe = 20.948[dB]
Irr_Noise = WALTZ
Irr_Pwidth = 92[us]
Decoupling = TRUE
Initial_Wait = 1[s]
Noe = TRUE
Noe_Time = 2[s]
Repetition_Time = 2.83361792[s]
abundance
0
0.1
0.2
0.3
0.4
200.0 190.0 180.0 170.0 160.0 150.0 140.0 130.0 120.0 110.0 100.0 90.0 80.0 70.0 60.0 50.0 40.0 30.0 20.0 10.0 0
163.088
143.754
136.056
132.270
131.802
125.602
124.620
122.178
121.148
118.639
116.932
114.729
111.448
109.836
107.222
41.484
39.500
X : parts per Million : Carbon13

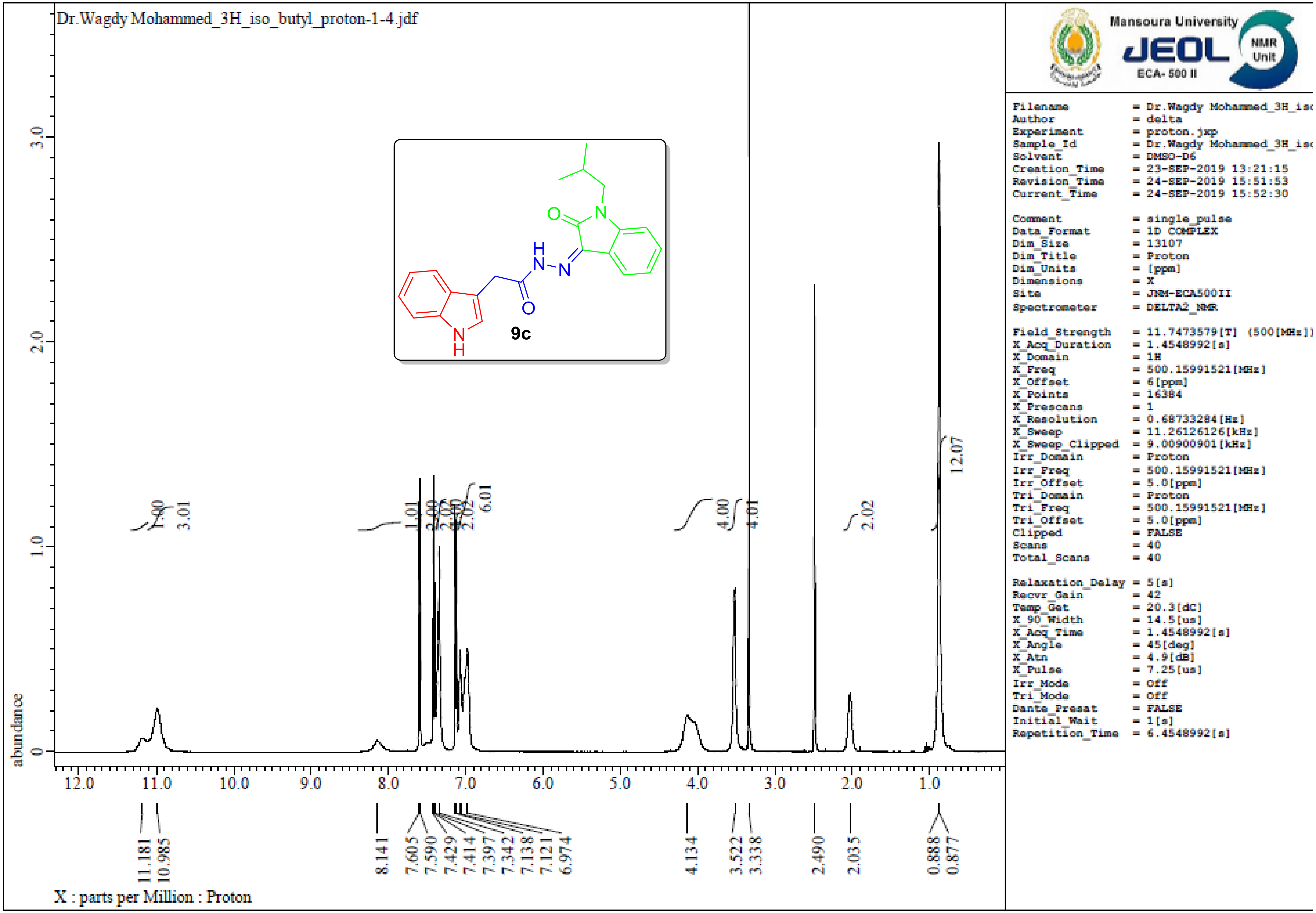
Dr.Wagdy Mohammed_3H_iso_butyl_proton-1-4.jdf
9c
Mansoura University
JEOL
ECA- 500 II
NMR Unit
Filename = Dr.Wagdy Mohammed_3H_iso
Author = delta
Experiment = proton.jxp
Sample_Id = Dr.Wagdy Mohammed_3H_iso
Solvent = DMSO-D6
Creation_Time = 23-SEP-2019 13:21:15
Revision_Time = 24-SEP-2019 15:51:53
Current_Time = 24-SEP-2019 15:52:30
Comment = single_pulse
Data_Format = 1D COMPLEX
Dim_Size = 13107
Dim_Title = Proton
Dim_Units = [ppm]
Dimensions = X
Site = JNM-ECA500II
Spectrometer = DELTA2_NMR
Field_Strength = 11.7473579[T] (500[MHz])
X_Acq_Duration = 1.4548992[s]
X_Domain = 1H
X_Freq = 500.15991521[MHz]
X_Offset = 6[ppm]
X_Points = 16384
X_Prescans = 1
X_Resolution = 0.68733284[Hz]
X_Sweep = 11.26126126[kHz]
X_Sweep_Clipped = 9.00900901[kHz]
Irr_Domain = Proton
Irr_Freq = 500.15991521[MHz]
Irr_Offset = 5.0[ppm]
Tri_Domain = Proton
Tri_Freq = 500.15991521[MHz]
Tri_Offset = 5.0[ppm]
Clipped = FALSE
Scans = 40
Total_Scans = 40
Relaxation_Delay = 5[s]
Recvr_Gain = 42
Temp_Get = 20.3[dC]
X_90_Width = 14.5[us]
X_Acq_Time = 1.4548992[s]
X_Angle = 45[deg]
X_Atn = 4.9[dB]
X_Pulse = 7.25[us]
Irr_Mode = Off
Tri_Mode = Off
Dante_Presat = FALSE
Initial_Wait = 1[s]
Repetition_Time = 6.4548992[s]
abundance
3.0
2.0
1.0
0
3.01
1.01
6.01
4.00
4.01
2.02
12.07
12.0
11.0
10.0
9.0
8.0
7.0
6.0
5.0
4.0
3.0
2.0
1.0
11.181
10.985
8.141
7.605
7.590
7.429
7.414
7.397
7.342
7.138
7.121
6.974
4.134
3.522
3.338
2.490
2.035
0.888
0.877
X : parts per Million : Proton

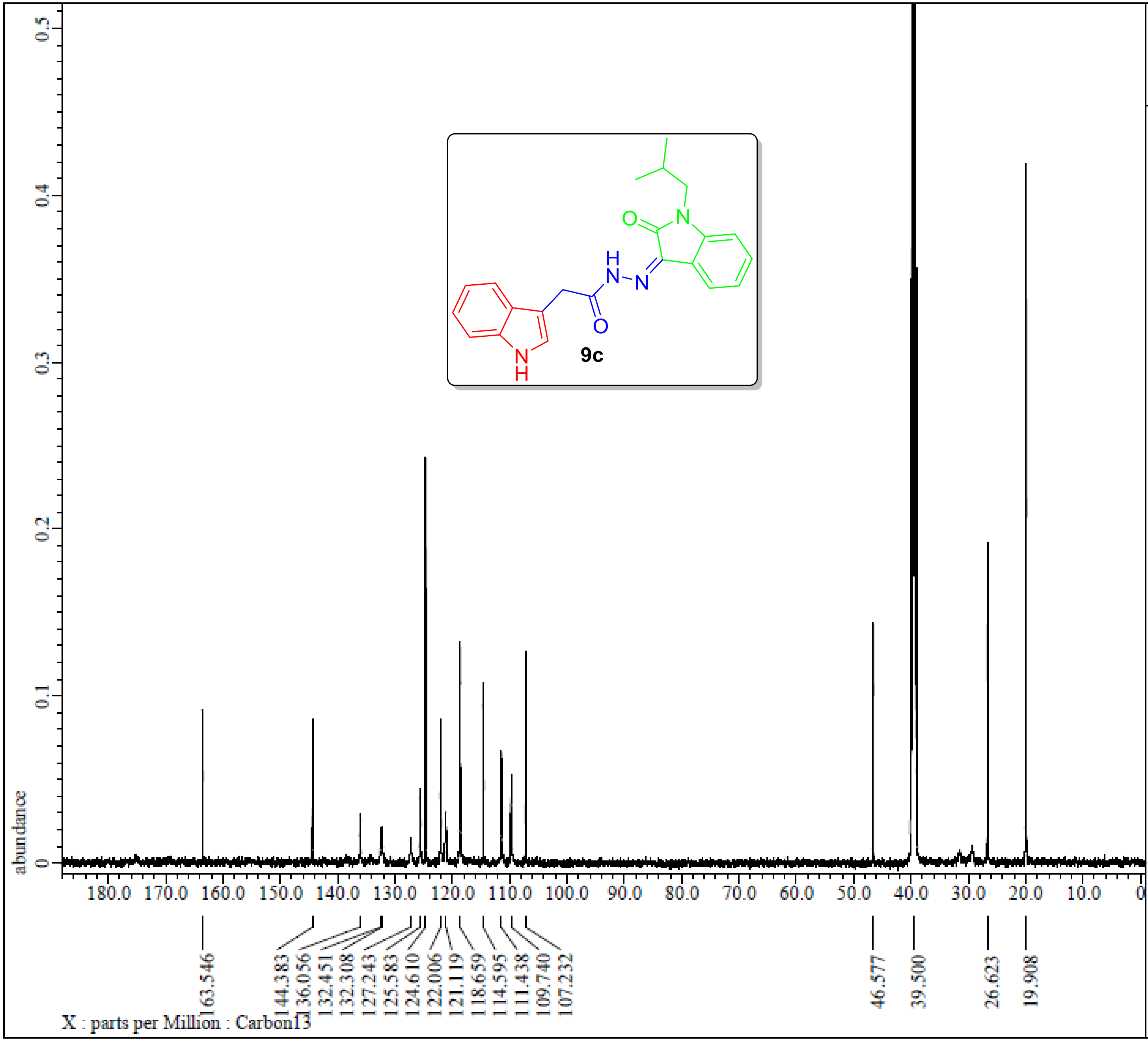

Mansoura University JEOL ECA-500 II NMR Unit

Filename	= Dr.Wagdy Mohammed_3H_isc
Author	= delta
Experiment	= carbon.jxp
Sample_Id	= Dr.Wagdy Mohammed_3H_isc
Solvent	= DMSO-D6
Creation_Time	= 25-SEP-2019 02:48:45
Revision_Time	= 25-SEP-2019 09:25:27
Current_Time	= 25-SEP-2019 09:28:42
Comment	= single pulse decoupled g
Data_Format	= 1D REAL
Dim_Size	= 26214
Dim_Title	= Carbon13
Dim_Units	= [ppm]
Dimensions	= X
Site	= JNM-ECA500II
Spectrometer	= DELTA2_NMR
Field_Strength	= 11.7473579[T] (500[MHz])
X_Acq_Duration	= 0.83361792[s]
X_Domain	= 13C
X_Freq	= 125.76529768[MHz]
X_Offset	= 100[ppm]
X_Points	= 32768
X_Prescans	= 4
X_Resolution	= 1.19959034[Hz]
X_Sweep	= 39.3081761[kHz]
X_Sweep_Clipped	= 31.44654088[kHz]
Irr_Domain	= Proton
Irr_Freq	= 500.15991521[MHz]
Irr_Offset	= 5.0[ppm]
Clipped	= FALSE
Scans	= 3700
Total_Scans	= 3700
Relaxation_Delay	= 2[s]
Recvr_Gain	= 56
Temp_Get	= 18.8[dC]
X_90_Width	= 52.78[us]
X_Acq_Time	= 0.83361792[s]
X_Angle	= 30[deg]
X_Atn	= 9.7[dB]
X_Pulse	= 17.59333333[us]
Irr_Atn_Dec	= 20.948[dB]
Irr_Atn_Noe	= 20.948[dB]
Irr_Noise	= WALTZ
Irr_Pwidth	= 92[us]
Decoupling	= TRUE
Initial_Wait	= 1[s]
Noe	= TRUE
Noe_Time	= 2[s]
Repetition_Time	= 2.83361792[s]

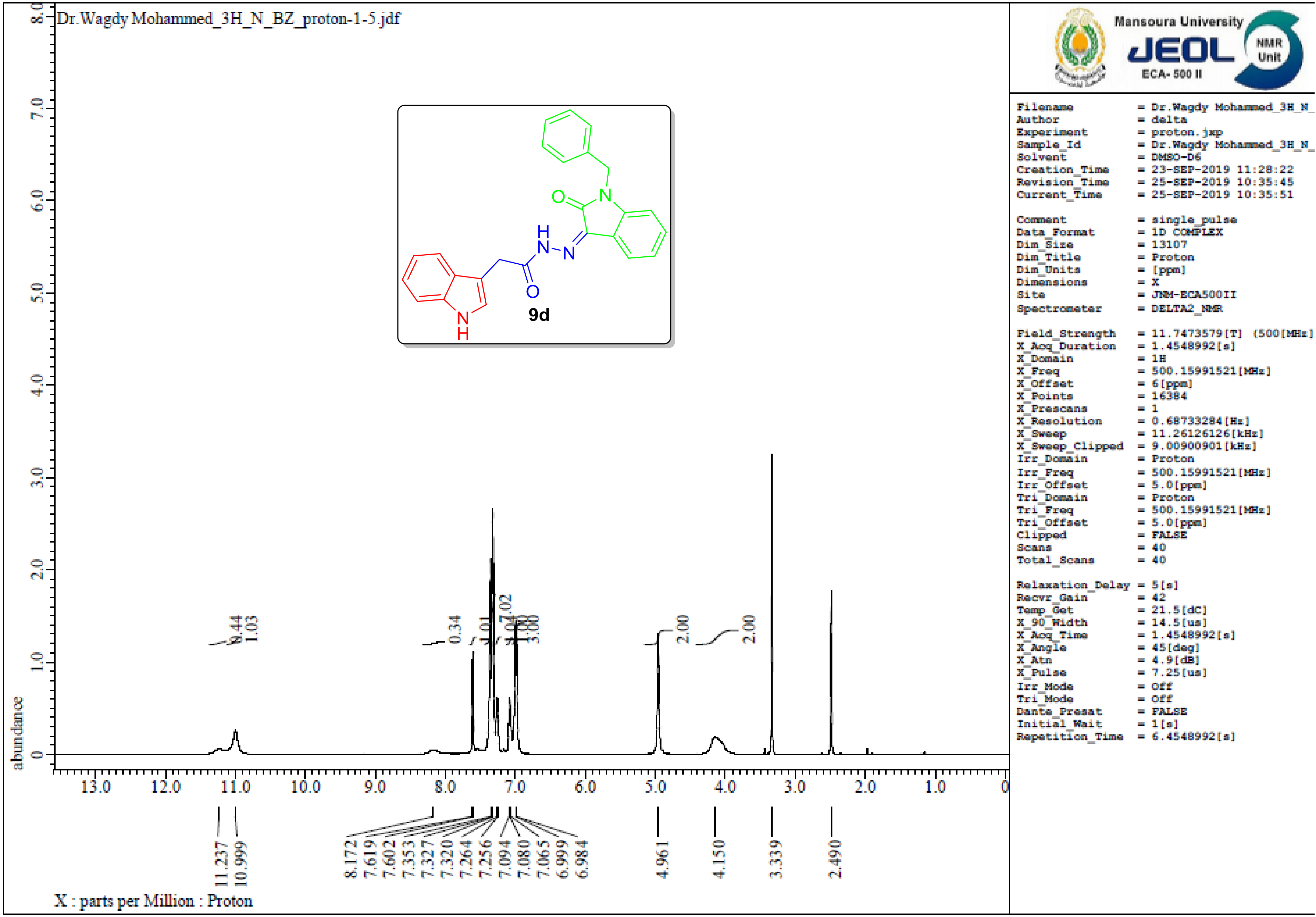
Dr.Wagdy Mohammed_3H_N_BZ_proton-1-5.jdf
9d
Mansoura University
JEOL
NMR Unit
ECA- 500 II
Filename = Dr.Wagdy Mohammed_3H_N_
Author = delta
Experiment = proton.jxp
Sample_Id = Dr.Wagdy Mohammed_3H_N_
Solvent = DMSO-D6
Creation_Time = 23-SEP-2019 11:28:22
Revision_Time = 25-SEP-2019 10:35:45
Current_Time = 25-SEP-2019 10:35:51
Comment = single_pulse
Data_Format = 1D COMPLEX
Dim_Size = 13107
Dim_Title = Proton
Dim_Units = [ppm]
Dimensions = X
Site = JNM-ECA500II
Spectrometer = DELTA2_NMR
Field_Strength = 11.7473579[T] (500[MHz]
X_Acq_Duration = 1.4548992[s]
X_Domain = 1H
X_Freq = 500.15991521[MHz]
X_Offset = 6[ppm]
X_Points = 16384
X_Prescans = 1
X_Resolution = 0.68733284[Hz]
X_Sweep = 11.26126126[kHz]
X_Sweep_Clipped = 9.00900901[kHz]
Irr_Domain = Proton
Irr_Freq = 500.15991521[MHz]
Irr_Offset = 5.0[ppm]
Tri_Domain = Proton
Tri_Freq = 500.15991521[MHz]
Tri_Offset = 5.0[ppm]
Clipped = FALSE
Scans = 40
Total_Scans = 40
Relaxation_Delay = 5[s]
Recvr_Gain = 42
Temp_Get = 21.5[dC]
X_90_Width = 14.5[us]
X_Acq_Time = 1.4548992[s]
X_Angle = 45[deg]
X_Atn = 4.9[dB]
X_Pulse = 7.25[us]
Irr_Mode = Off
Tri_Mode = Off
Dante_Presat = FALSE
Initial_Wait = 1[s]
Repetition_Time = 6.4548992[s]
abundance
X : parts per Million : Proton
11.237
10.999
8.172
7.619
7.602
7.353
7.327
7.320
7.264
7.256
7.094
7.080
7.065
6.999
6.984
4.961
4.150
3.339
2.490

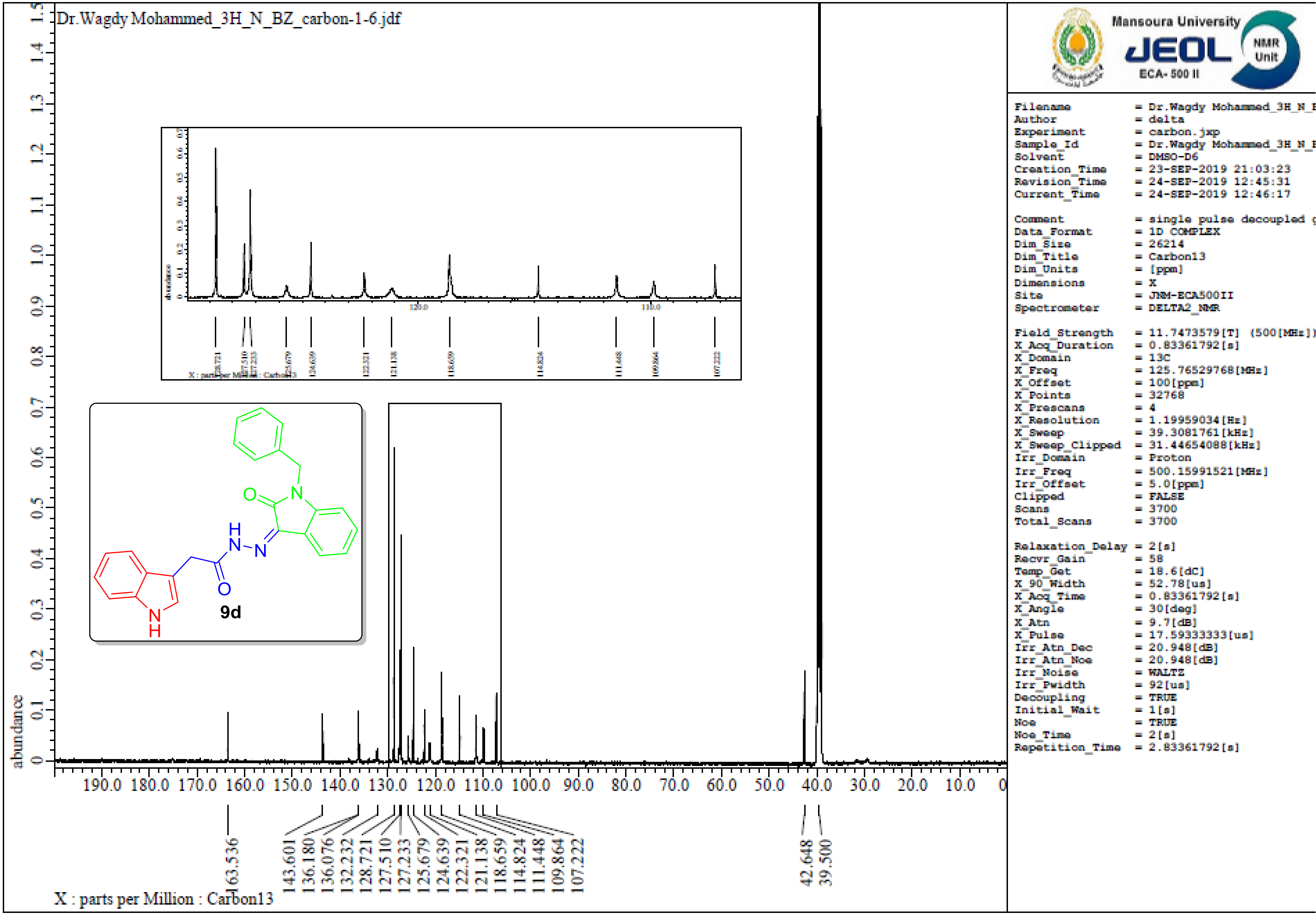

Dr.Wagdy Mohammed_3H_N_BZ_carbon-1-6.jdf
abundance
X : parts per Million : Carbon13
190.0 180.0 170.0 160.0 150.0 140.0 130.0 120.0 110.0 100.0 90.0 80.0 70.0 60.0 50.0 40.0 30.0 20.0 10.0 0
163.536
143.601
136.180
136.076
132.232
128.721
127.510
127.233
125.679
124.639
122.321
121.138
118.659
114.824
111.448
109.864
107.222
42.648
39.500
9d
Mansoura University
JEOL
NMR Unit
ECA- 500 II
Filename = Dr.Wagdy Mohammed_3H_N_B
Author = delta
Experiment = carbon.jxp
Sample_Id = Dr.Wagdy Mohammed_3H_N_B
Solvent = DMSO-D6
Creation_Time = 23-SEP-2019 21:03:23
Revision_Time = 24-SEP-2019 12:45:31
Current_Time = 24-SEP-2019 12:46:17
Comment = single pulse decoupled g
Data_Format = 1D COMPLEX
Dim_Size = 26214
Dim_Title = Carbon13
Dim_Units = [ppm]
Dimensions = X
Site = JNM-ECA500II
Spectrometer = DELTA2_NMR
Field_Strength = 11.7473579[T] (500[MHz])
X_Acq_Duration = 0.83361792[s]
X_Domain = 13C
X_Freq = 125.76529768[MHz]
X_Offset = 100[ppm]
X_Points = 32768
X_Prescans = 4
X_Resolution = 1.19959034[Hz]
X_Sweep = 39.3081761[kHz]
X_Sweep_Clipped = 31.44654088[kHz]
Irr_Domain = Proton
Irr_Freq = 500.15991521[MHz]
Irr_Offset = 5.0[ppm]
Clipped = FALSE
Scans = 3700
Total_Scans = 3700
Relaxation_Delay = 2[s]
Recvr_Gain = 58
Temp_Get = 18.6[dC]
X_90_Width = 52.78[us]
X_Acq_Time = 0.83361792[s]
X_Angle = 30[deg]
X_Atn = 9.7[dB]
X_Pulse = 17.59333333[us]
Irr_Atn_Dec = 20.948[dB]
Irr_Atn_Noe = 20.948[dB]
Irr_Noise = WALTZ
Irr_Pwidth = 92[us]
Decoupling = TRUE
Initial_Wait = 1[s]
Noe = TRUE
Noe_Time = 2[s]
Repetition_Time = 2.83361792[s]

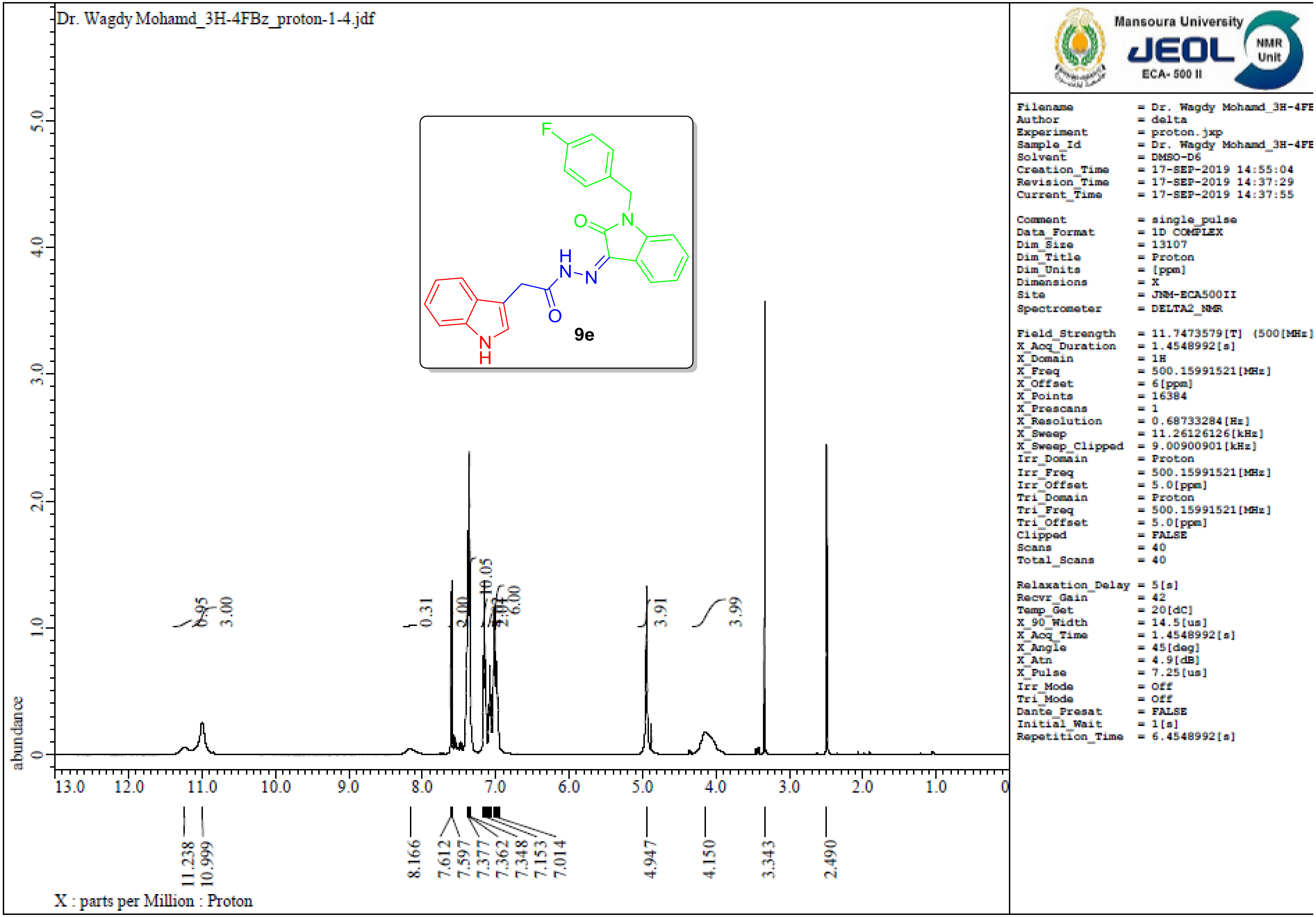

Dr. Wagdy Mohamd_3H-4FBz_proton-1-4.jdf
Mansoura University
JEOL
NMR Unit
ECA- 500 II
F
O
N
H
N
N
O
N
H
9e
Filename = Dr. Wagdy Mohamd_3H-4FB
Author = delta
Experiment = proton.jxp
Sample_Id = Dr. Wagdy Mohamd_3H-4FB
Solvent = DMSO-D6
Creation_Time = 17-SEP-2019 14:55:04
Revision_Time = 17-SEP-2019 14:37:29
Current_Time = 17-SEP-2019 14:37:55
Comment = single_pulse
Data_Format = 1D COMPLEX
Dim_Size = 13107
Dim_Title = Proton
Dim_Units = [ppm]
Dimensions = X
Site = JNM-ECA500II
Spectrometer = DELTA2_NMR
Field_Strength = 11.7473579[T] (500[MHz
X_Acq_Duration = 1.4548992[s]
X_Domain = 1H
X_Freq = 500.15991521[MHz]
X_Offset = 6[ppm]
X_Points = 16384
X_Prescans = 1
X_Resolution = 0.68733284[Hz]
X_Sweep = 11.26126126[kHz]
X_Sweep_Clipped = 9.00900901[kHz]
Irr_Domain = Proton
Irr_Freq = 500.15991521[MHz]
Irr_Offset = 5.0[ppm]
Tri_Domain = Proton
Tri_Freq = 500.15991521[MHz]
Tri_Offset = 5.0[ppm]
Clipped = FALSE
Scans = 40
Total_Scans = 40
Relaxation_Delay = 5[s]
Recvr_Gain = 42
Temp_Get = 20[dC]
X_90_Width = 14.5[us]
X_Acq_Time = 1.4548992[s]
X_Angle = 45[deg]
X_Atn = 4.9[dB]
X_Pulse = 7.25[us]
Irr_Mode = Off
Tri_Mode = Off
Dante_Presat = FALSE
Initial_Wait = 1[s]
Repetition_Time = 6.4548992[s]
abundance
0
1.0
2.0
3.0
4.0
5.0
3.00
0.31
10.05
6.00
3.91
3.99
13.0
12.0
11.0
10.0
9.0
8.0
7.0
6.0
5.0
4.0
3.0
2.0
1.0
11.238
10.999
8.166
7.612
7.597
7.377
7.362
7.348
7.153
7.014
4.947
4.150
3.343
2.490
X : parts per Million : Proton

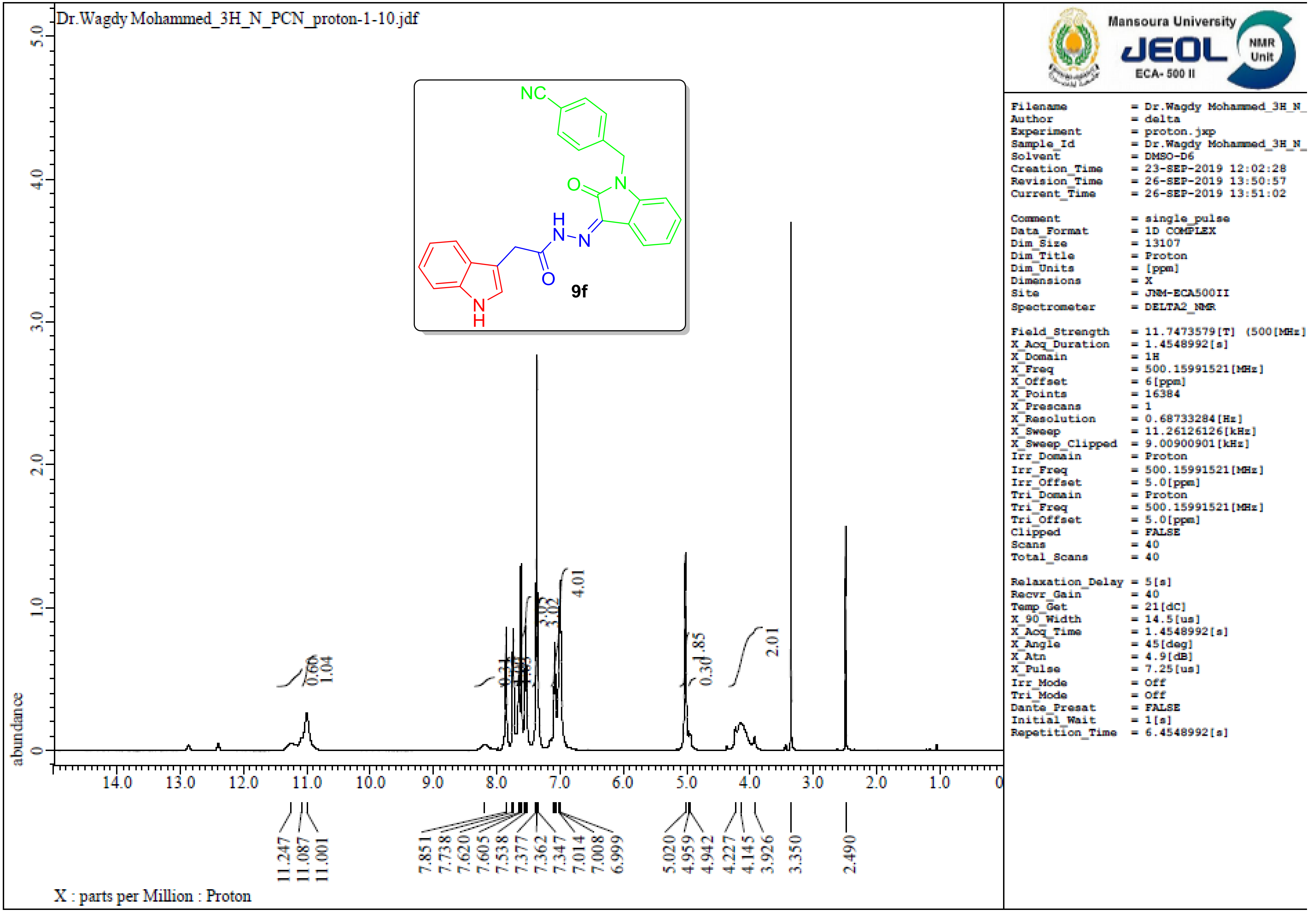
Dr.Wagdy Mohammed_3H_N_PCN_proton-1-10.jdf
NC
O
N
H
N
N
O
N
H
9f
abundance
X : parts per Million : Proton
11.247
11.087
11.001
7.851
7.738
7.620
7.605
7.538
7.377
7.362
7.347
7.014
7.008
6.999
5.020
4.959
4.942
4.227
4.145
3.926
3.350
2.490
Mansoura University
JEOL
NMR Unit
ECA- 500 II
Filename = Dr.Wagdy Mohammed_3H_N_
Author = delta
Experiment = proton.jxp
Sample_Id = Dr.Wagdy Mohammed_3H_N_
Solvent = DMSO-D6
Creation_Time = 23-SEP-2019 12:02:28
Revision_Time = 26-SEP-2019 13:50:57
Current_Time = 26-SEP-2019 13:51:02
Comment = single_pulse
Data_Format = 1D COMPLEX
Dim_Size = 13107
Dim_Title = Proton
Dim_Units = [ppm]
Dimensions = X
Site = JNM-ECA500II
Spectrometer = DELTA2_NMR
Field_Strength = 11.7473579[T] (500[MHz]
X_Acq_Duration = 1.4548992[s]
X_Domain = 1H
X_Freq = 500.15991521[MHz]
X_Offset = 6[ppm]
X_Points = 16384
X_Prescans = 1
X_Resolution = 0.68733284[Hz]
X_Sweep = 11.26126126[kHz]
X_Sweep_Clipped = 9.00900901[kHz]
Irr_Domain = Proton
Irr_Freq = 500.15991521[MHz]
Irr_Offset = 5.0[ppm]
Tri_Domain = Proton
Tri_Freq = 500.15991521[MHz]
Tri_Offset = 5.0[ppm]
Clipped = FALSE
Scans = 40
Total_Scans = 40
Relaxation_Delay = 5[s]
Recvr_Gain = 40
Temp_Get = 21[dC]
X_90_Width = 14.5[us]
X_Acq_Time = 1.4548992[s]
X_Angle = 45[deg]
X_Atn = 4.9[dB]
X_Pulse = 7.25[us]
Irr_Mode = Off
Tri_Mode = Off
Dante_Presat = FALSE
Initial_Wait = 1[s]
Repetition_Time = 6.4548992[s]

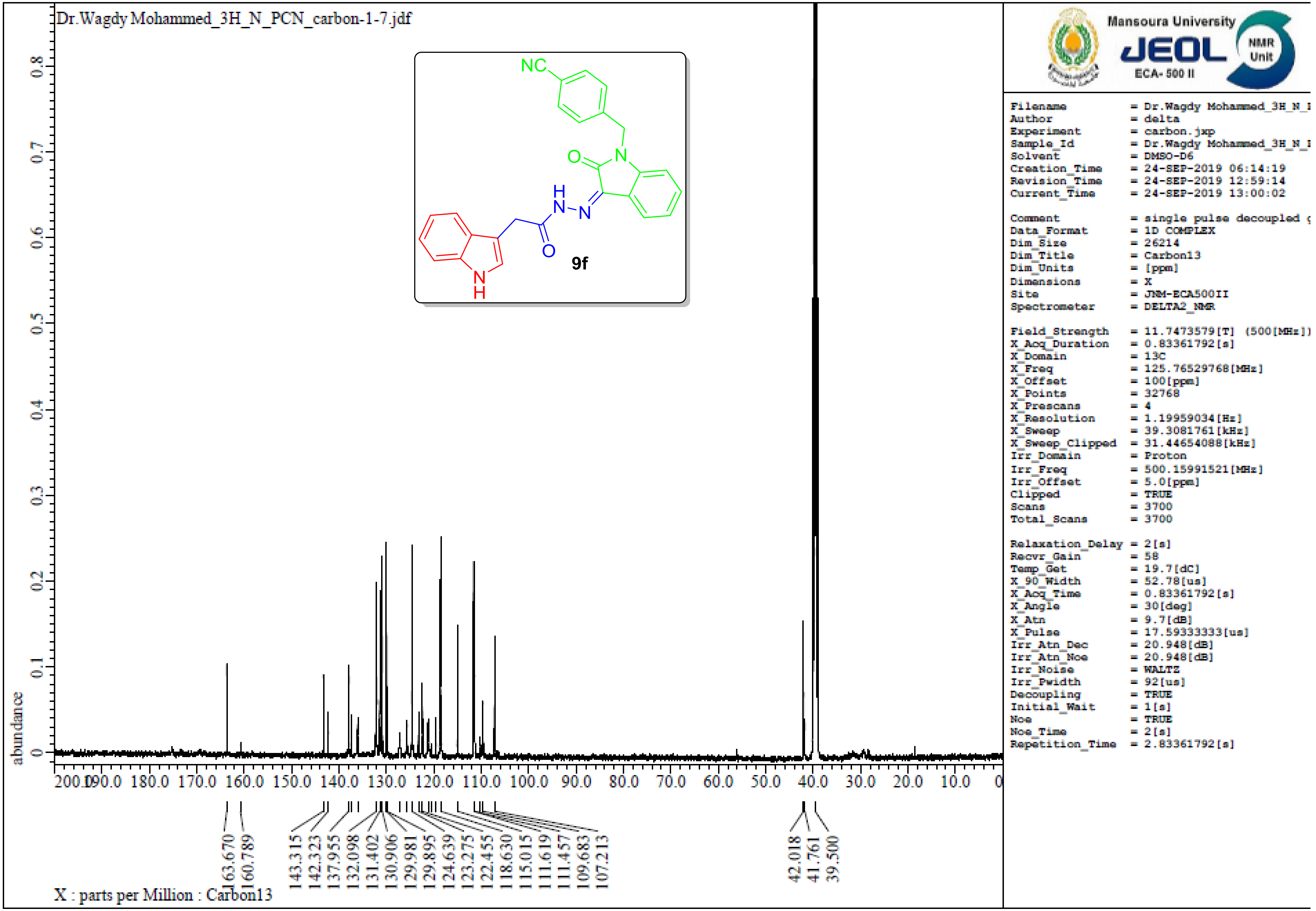
Dr.Wagdy Mohammed_3H_N_PCN_carbon-1-7.jdf
NC
O
N
H
N
N
N
H
O
9f
abundance
0 0.1 0.2 0.3 0.4 0.5 0.6 0.7 0.8
200.0 190.0 180.0 170.0 160.0 150.0 140.0 130.0 120.0 110.0 100.0 90.0 80.0 70.0 60.0 50.0 40.0 30.0 20.0 10.0 0
163.670 160.789 143.315 142.323 137.955 132.098 131.402 130.906 129.981 129.895 124.639 123.275 122.455 118.630 115.015 111.619 111.457 109.683 107.213 42.018 41.761 39.500
X : parts per Million : Carbon13
Mansoura University
JEOL
ECA- 500 II
NMR Unit
Filename = Dr.Wagdy Mohammed_3H_N_I
Author = delta
Experiment = carbon.jxp
Sample_Id = Dr.Wagdy Mohammed_3H_N_I
Solvent = DMSO-D6
Creation_Time = 24-SEP-2019 06:14:19
Revision_Time = 24-SEP-2019 12:59:14
Current_Time = 24-SEP-2019 13:00:02
Comment = single pulse decoupled g
Data_Format = 1D COMPLEX
Dim_Size = 26214
Dim_Title = Carbon13
Dim_Units = [ppm]
Dimensions = X
Site = JNM-ECA500II
Spectrometer = DELTA2_NMR
Field_Strength = 11.7473579[T] (500[MHz])
X_Acq_Duration = 0.83361792[s]
X_Domain = 13C
X_Freq = 125.76529768[MHz]
X_Offset = 100[ppm]
X_Points = 32768
X_Prescans = 4
X_Resolution = 1.19959034[Hz]
X_Sweep = 39.3081761[kHz]
X_Sweep_Clipped = 31.44654088[kHz]
Irr_Domain = Proton
Irr_Freq = 500.15991521[MHz]
Irr_Offset = 5.0[ppm]
Clipped = TRUE
Scans = 3700
Total_Scans = 3700
Relaxation_Delay = 2[s]
Recvr_Gain = 58
Temp_Get = 19.7[dC]
X_90_Width = 52.78[us]
X_Acq_Time = 0.83361792[s]
X_Angle = 30[deg]
X_Atn = 9.7[dB]
X_Pulse = 17.59333333[us]
Irr_Atn_Dec = 20.948[dB]
Irr_Atn_Noe = 20.948[dB]
Irr_Noise = WALTZ
Irr_Pwidth = 92[us]
Decoupling = TRUE
Initial_Wait = 1[s]
Noe = TRUE
Noe_Time = 2[s]
Repetition_Time = 2.83361792[s]

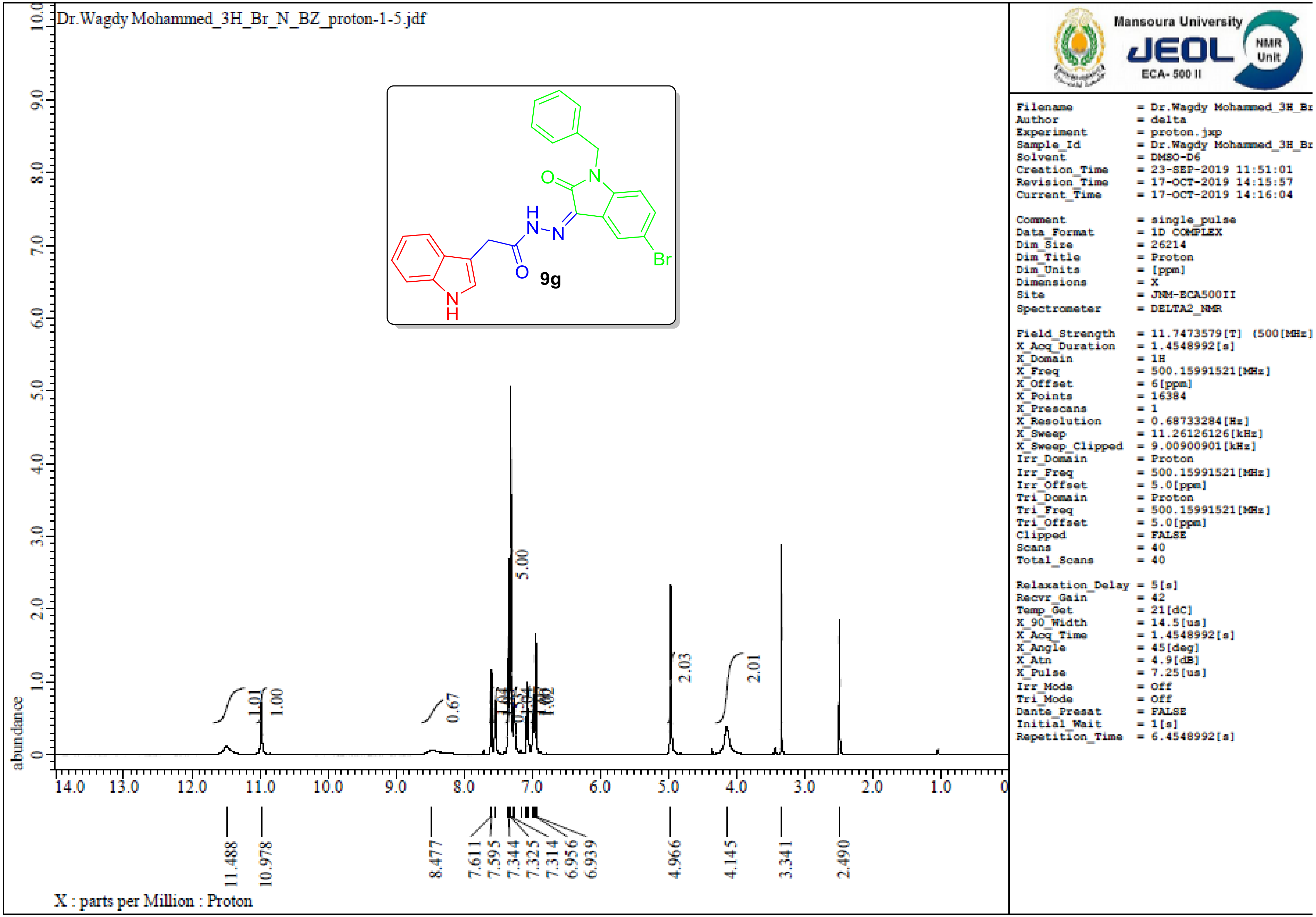

Dr.Wagdy Mohammed_3H_Br_N_BZ_proton-1-5.jdf
9g
Br
Mansoura University
JEOL
NMR Unit
ECA- 500 II
Filename = Dr.Wagdy Mohammed_3H_Br
Author = delta
Experiment = proton.jxp
Sample_Id = Dr.Wagdy Mohammed_3H_Br
Solvent = DMSO-D6
Creation_Time = 23-SEP-2019 11:51:01
Revision_Time = 17-OCT-2019 14:15:57
Current_Time = 17-OCT-2019 14:16:04
Comment = single_pulse
Data_Format = 1D COMPLEX
Dim_Size = 26214
Dim_Title = Proton
Dim_Units = [ppm]
Dimensions = X
Site = JNM-ECA500II
Spectrometer = DELTA2_NMR
Field_Strength = 11.7473579[T] (500[MHz]
X_Acq_Duration = 1.4548992[s]
X_Domain = 1H
X_Freq = 500.15991521[MHz]
X_Offset = 6[ppm]
X_Points = 16384
X_Prescans = 1
X_Resolution = 0.68733284[Hz]
X_Sweep = 11.26126126[kHz]
X_Sweep_Clipped = 9.00900901[kHz]
Irr_Domain = Proton
Irr_Freq = 500.15991521[MHz]
Irr_Offset = 5.0[ppm]
Tri_Domain = Proton
Tri_Freq = 500.15991521[MHz]
Tri_Offset = 5.0[ppm]
Clipped = FALSE
Scans = 40
Total_Scans = 40
Relaxation_Delay = 5[s]
Recvr_Gain = 42
Temp_Get = 21[dC]
X_90_Width = 14.5[us]
X_Acq_Time = 1.4548992[s]
X_Angle = 45[deg]
X_Atn = 4.9[dB]
X_Pulse = 7.25[us]
Irr_Mode = Off
Tri_Mode = Off
Dante_Presat = FALSE
Initial_Wait = 1[s]
Repetition_Time = 6.4548992[s]
abundance
1.01
1.00
0.67
5.00
2.03
2.01
11.488
10.978
8.477
7.611
7.595
7.344
7.325
7.314
6.956
6.939
4.966
4.145
3.341
2.490
X : parts per Million : Proton

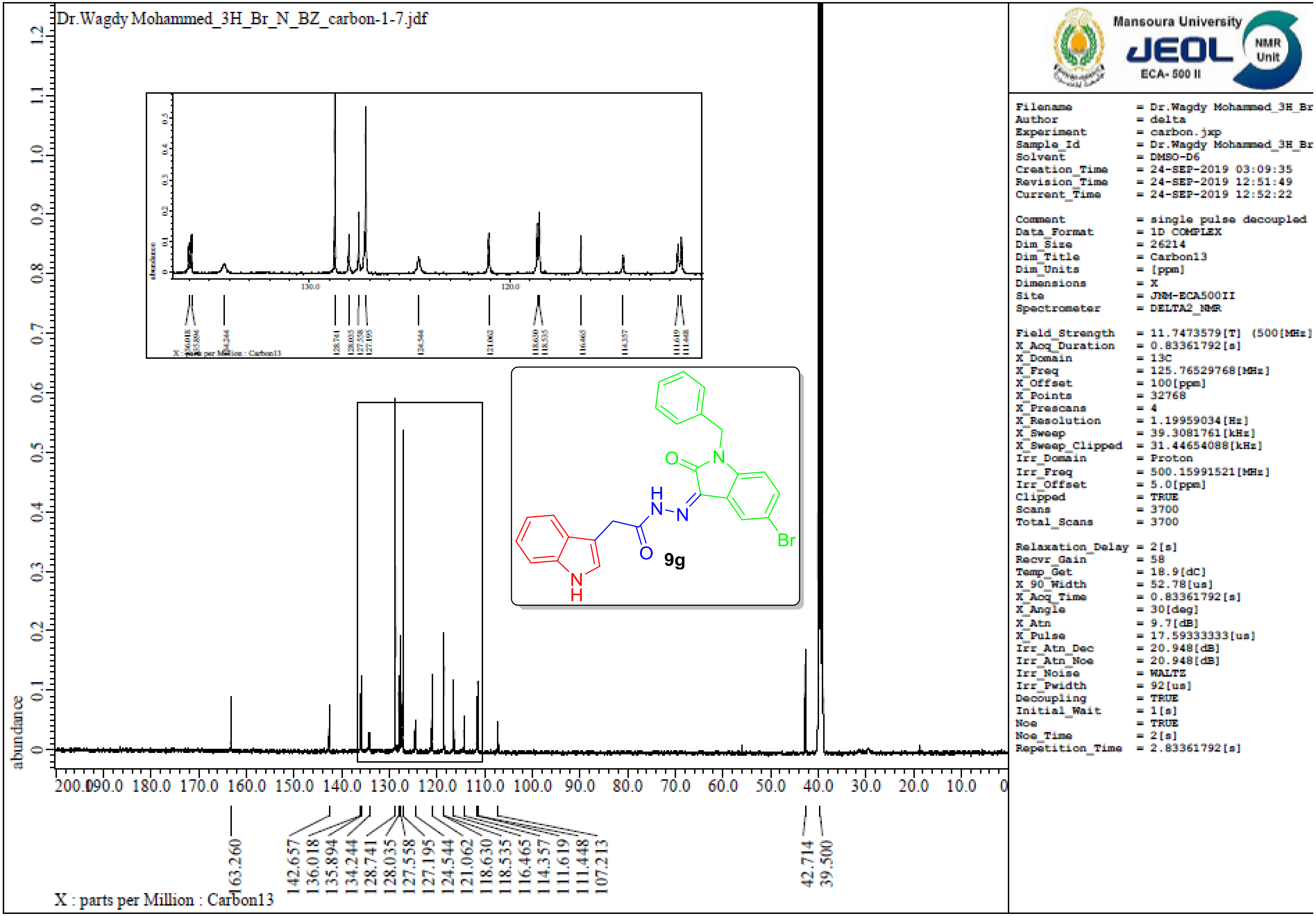
Dr.Wagdy Mohammed_3H_Br_N_BZ_carbon-1-7.jdf
Mansoura University
JEOL
NMR Unit
ECA- 500 II
Filename = Dr.Wagdy Mohammed_3H_Br
Author = delta
Experiment = carbon.jxp
Sample_Id = Dr.Wagdy Mohammed_3H_Br
Solvent = DMSO-D6
Creation_Time = 24-SEP-2019 03:09:35
Revision_Time = 24-SEP-2019 12:51:49
Current_Time = 24-SEP-2019 12:52:22
Comment = single pulse decoupled
Data_Format = 1D COMPLEX
Dim_Size = 26214
Dim_Title = Carbon13
Dim_Units = [ppm]
Dimensions = X
Site = JNM-ECA500II
Spectrometer = DELTA2_NMR
Field_Strength = 11.7473579[T] (500[MHz]
X_Acq_Duration = 0.83361792[s]
X_Domain = 13C
X_Freq = 125.76529768[MHz]
X_Offset = 100[ppm]
X_Points = 32768
X_Prescans = 4
X_Resolution = 1.19959034[Hz]
X_Sweep = 39.3081761[kHz]
X_Sweep_Clipped = 31.44654088[kHz]
Irr_Domain = Proton
Irr_Freq = 500.15991521[MHz]
Irr_Offset = 5.0[ppm]
Clipped = TRUE
Scans = 3700
Total_Scans = 3700
Relaxation_Delay = 2[s]
Recvr_Gain = 58
Temp_Get = 18.9[dC]
X_90_Width = 52.78[us]
X_Acq_Time = 0.83361792[s]
X_Angle = 30[deg]
X_Atn = 9.7[dB]
X_Pulse = 17.59333333[us]
Irr_Atn_Dec = 20.948[dB]
Irr_Atn_Noe = 20.948[dB]
Irr_Noise = WALTZ
Irr_Pwidth = 92[us]
Decoupling = TRUE
Initial_Wait = 1[s]
Noe = TRUE
Noe_Time = 2[s]
Repetition_Time = 2.83361792[s]
9g
Br
abundance
X : parts per Million : Carbon13
163.260
142.657
136.018
135.894
134.244
128.741
128.035
127.558
127.195
124.544
121.062
118.630
118.535
116.465
114.357
111.619
111.448
107.213
42.714
39.500

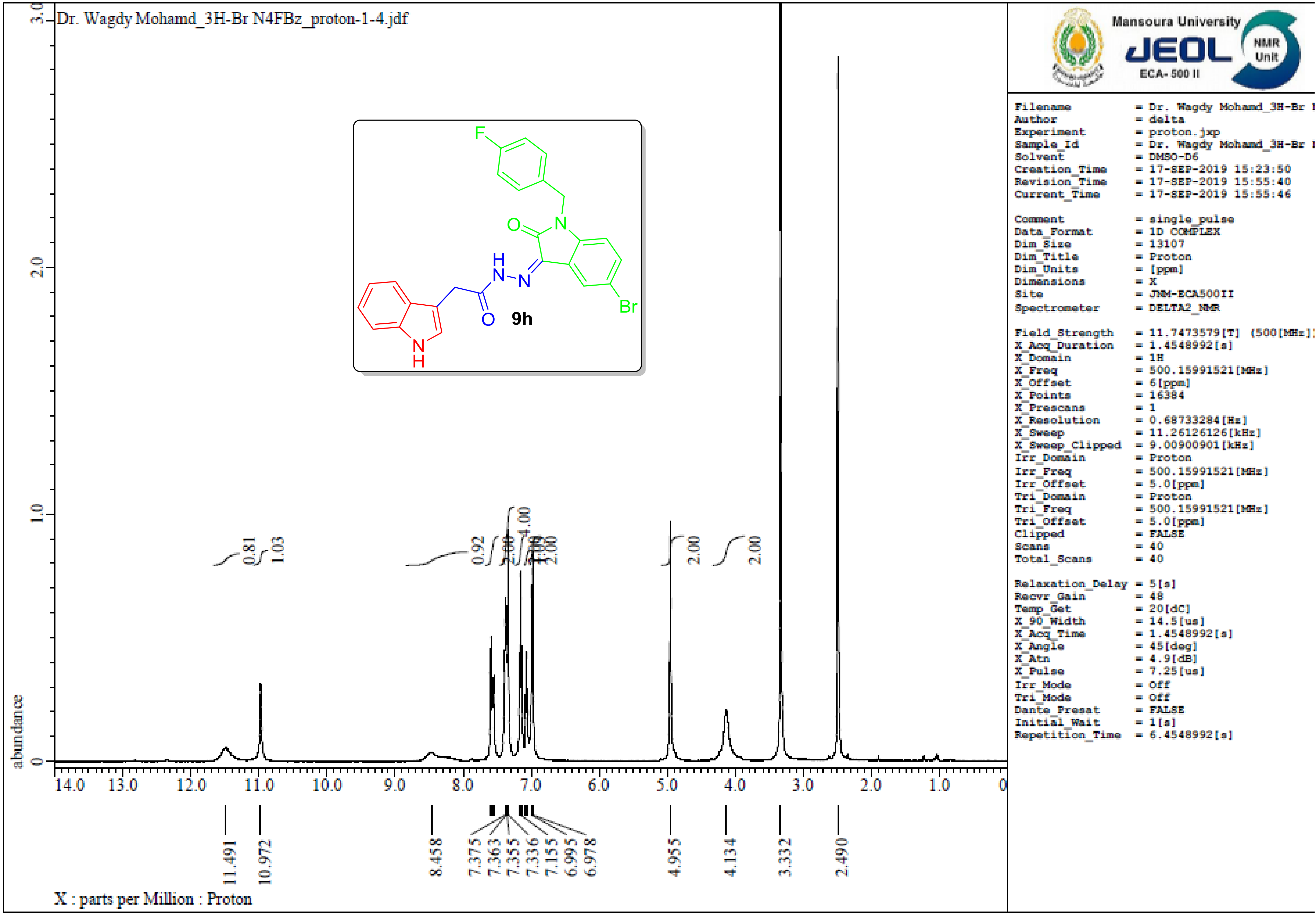
Dr. Wagdy Mohamd_3H-Br N4FBz_proton-1-4.jdf
9h
Mansoura University
JEOL
ECA- 500 II
NMR Unit
Filename = Dr. Wagdy Mohamd_3H-Br
Author = delta
Experiment = proton.jxp
Sample_Id = Dr. Wagdy Mohamd_3H-Br
Solvent = DMSO-D6
Creation_Time = 17-SEP-2019 15:23:50
Revision_Time = 17-SEP-2019 15:55:40
Current_Time = 17-SEP-2019 15:55:46
Comment = single_pulse
Data_Format = 1D COMPLEX
Dim_Size = 13107
Dim_Title = Proton
Dim_Units = [ppm]
Dimensions = X
Site = JNM-ECA500II
Spectrometer = DELTA2_NMR
Field_Strength = 11.7473579[T] (500[MHz])
X_Acq_Duration = 1.4548992[s]
X_Domain = 1H
X_Freq = 500.15991521[MHz]
X_Offset = 6[ppm]
X_Points = 16384
X_Prescans = 1
X_Resolution = 0.68733284[Hz]
X_Sweep = 11.26126126[kHz]
X_Sweep_Clipped = 9.00900901[kHz]
Irr_Domain = Proton
Irr_Freq = 500.15991521[MHz]
Irr_Offset = 5.0[ppm]
Tri_Domain = Proton
Tri_Freq = 500.15991521[MHz]
Tri_Offset = 5.0[ppm]
Clipped = FALSE
Scans = 40
Total_Scans = 40
Relaxation_Delay = 5[s]
Recvr_Gain = 48
Temp_Get = 20[dC]
X_90_Width = 14.5[us]
X_Acq_Time = 1.4548992[s]
X_Angle = 45[deg]
X_Atn = 4.9[dB]
X_Pulse = 7.25[us]
Irr_Mode = Off
Tri_Mode = Off
Dante_Presat = FALSE
Initial_Wait = 1[s]
Repetition_Time = 6.4548992[s]
0.81
1.03
0.92
2.00
4.00
2.00
2.00
2.00
11.491
10.972
8.458
7.375
7.363
7.355
7.336
7.155
6.995
6.978
4.955
4.134
3.332
2.490
abundance
X : parts per Million : Proton

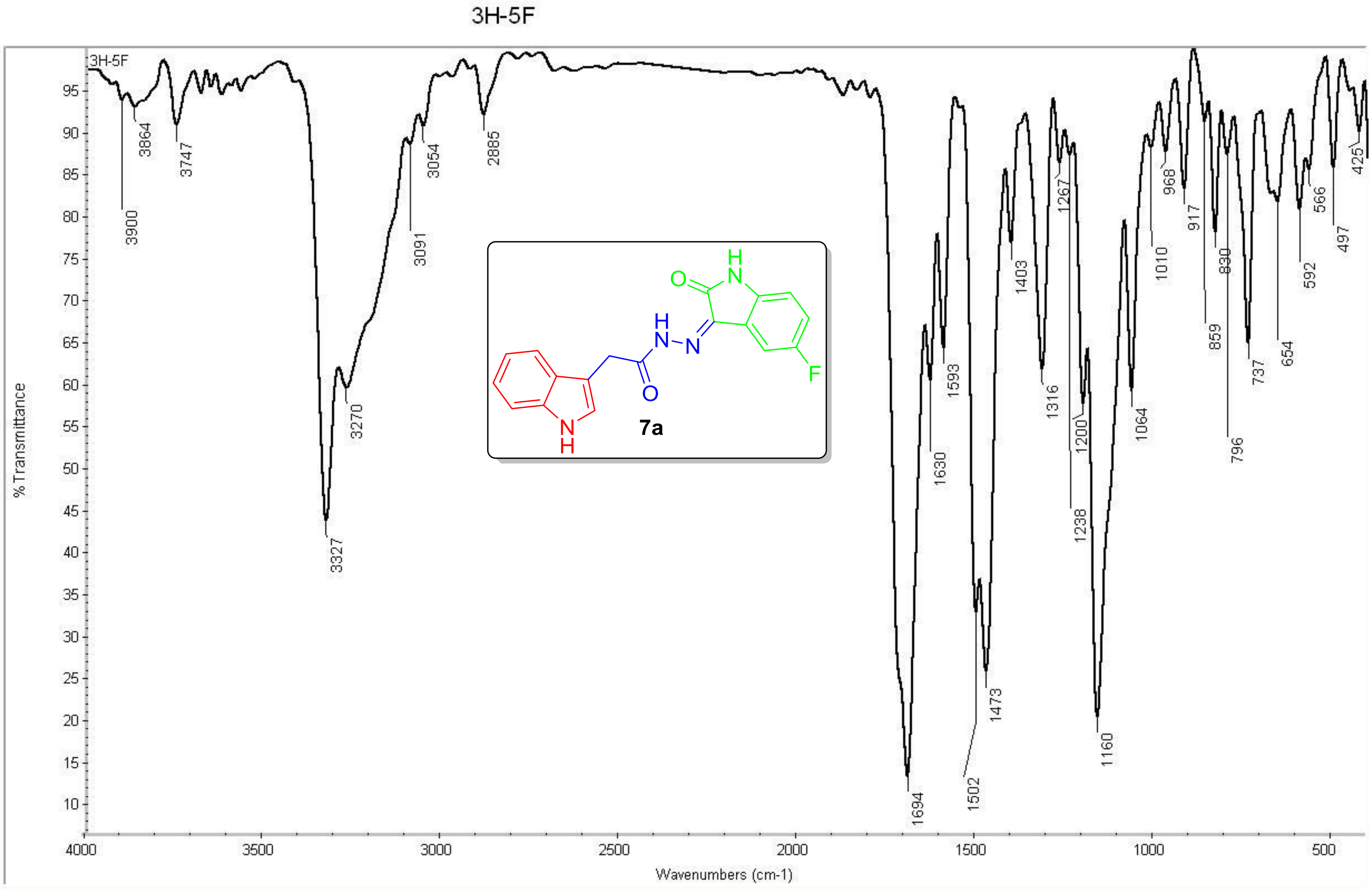

3H-5F
3H-5F
% Transmittance
Wavenumbers (cm-1)
3900
3864
3747
3327
3270
3091
3054
2885
1694
1630
1593
1502
1473
1403
1316
1267
1238
1200
1160
1064
1010
968
917
859
830
796
737
654
592
566
497
425
7a

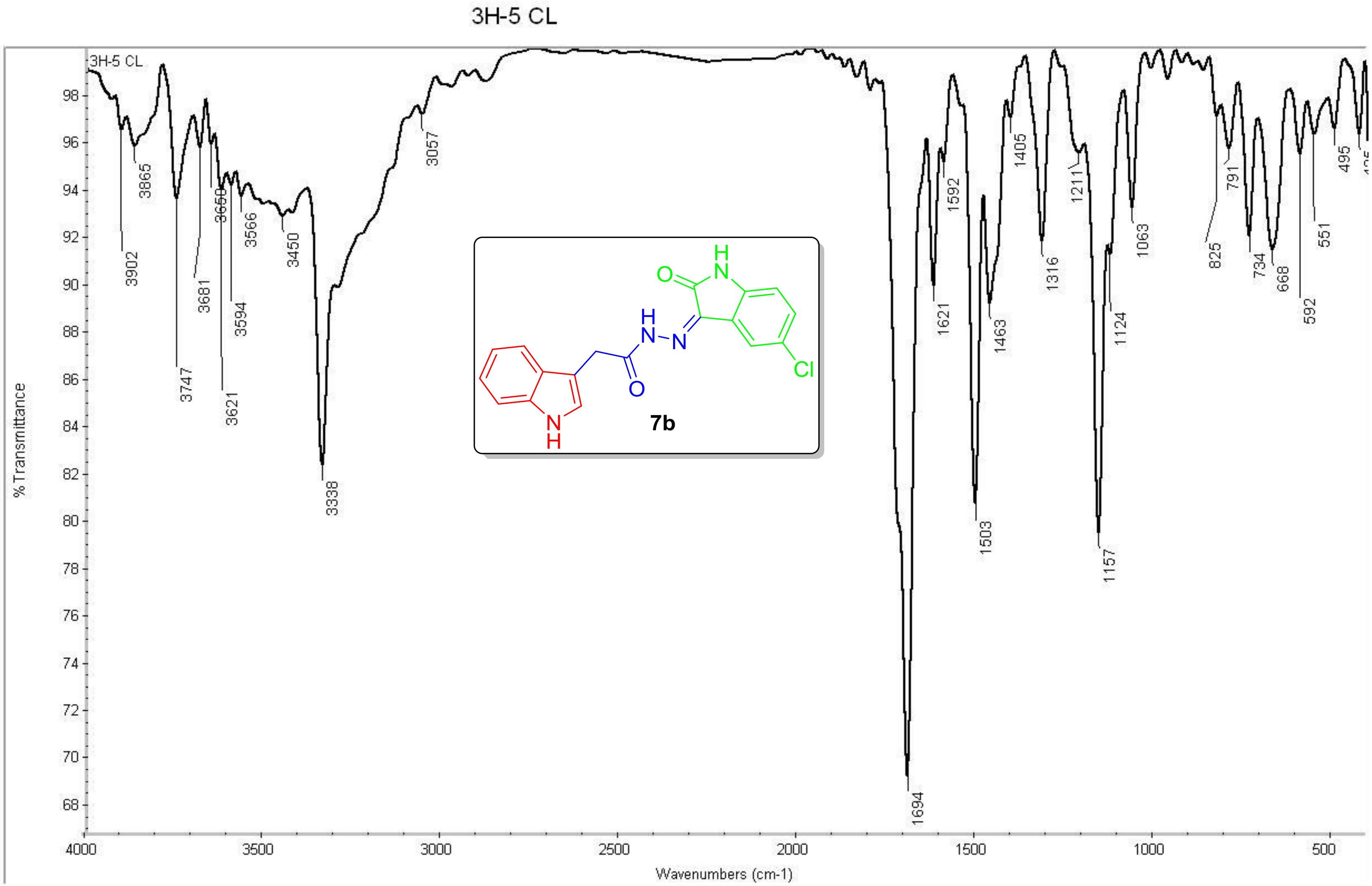
3H-5 CL
3H-5 CL
% Transmittance
Wavenumbers (cm-1)
4000
3500
3000
2500
2000
1500
1000
500
98
96
94
92
90
88
86
84
82
80
78
76
74
72
70
68
3902
3865
3747
3681
3650
3621
3594
3566
3450
3338
3057
1694
1621
1592
1503
1463
1405
1316
1211
1157
1124
1063
825
791
734
668
592
551
495
7b
Cl

3H-5Me

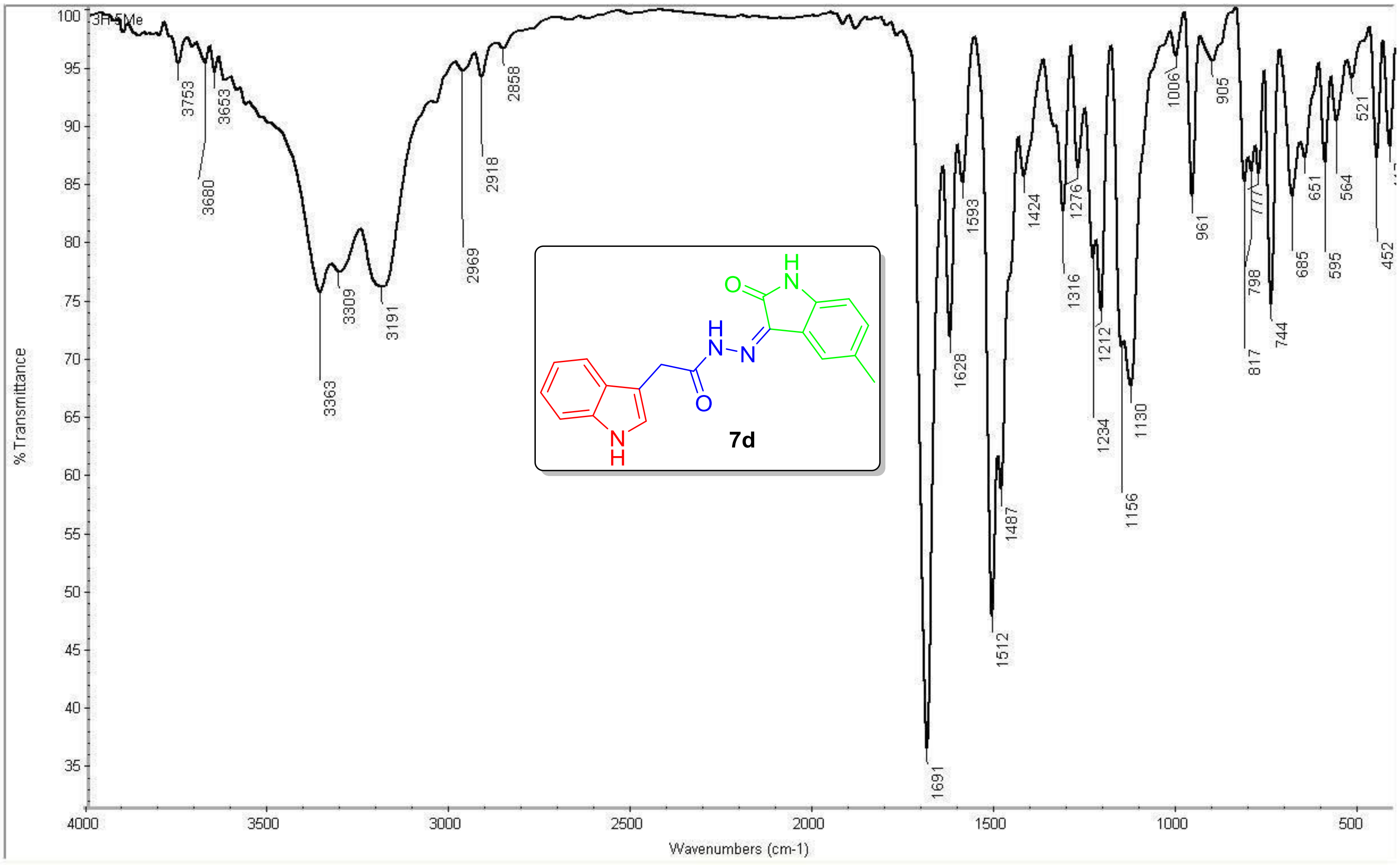

3H-5Me
% Transmittance
Wavenumbers (cm-1)
3753
3680
3653
3363
3309
3191
2969
2918
2858
1691
1628
1593
1512
1487
1424
1316
1276
1234
1212
1156
1130
1006
961
905
817
798
744
685
651
595
564
521
452
7d

3H-5-7 dimethyl

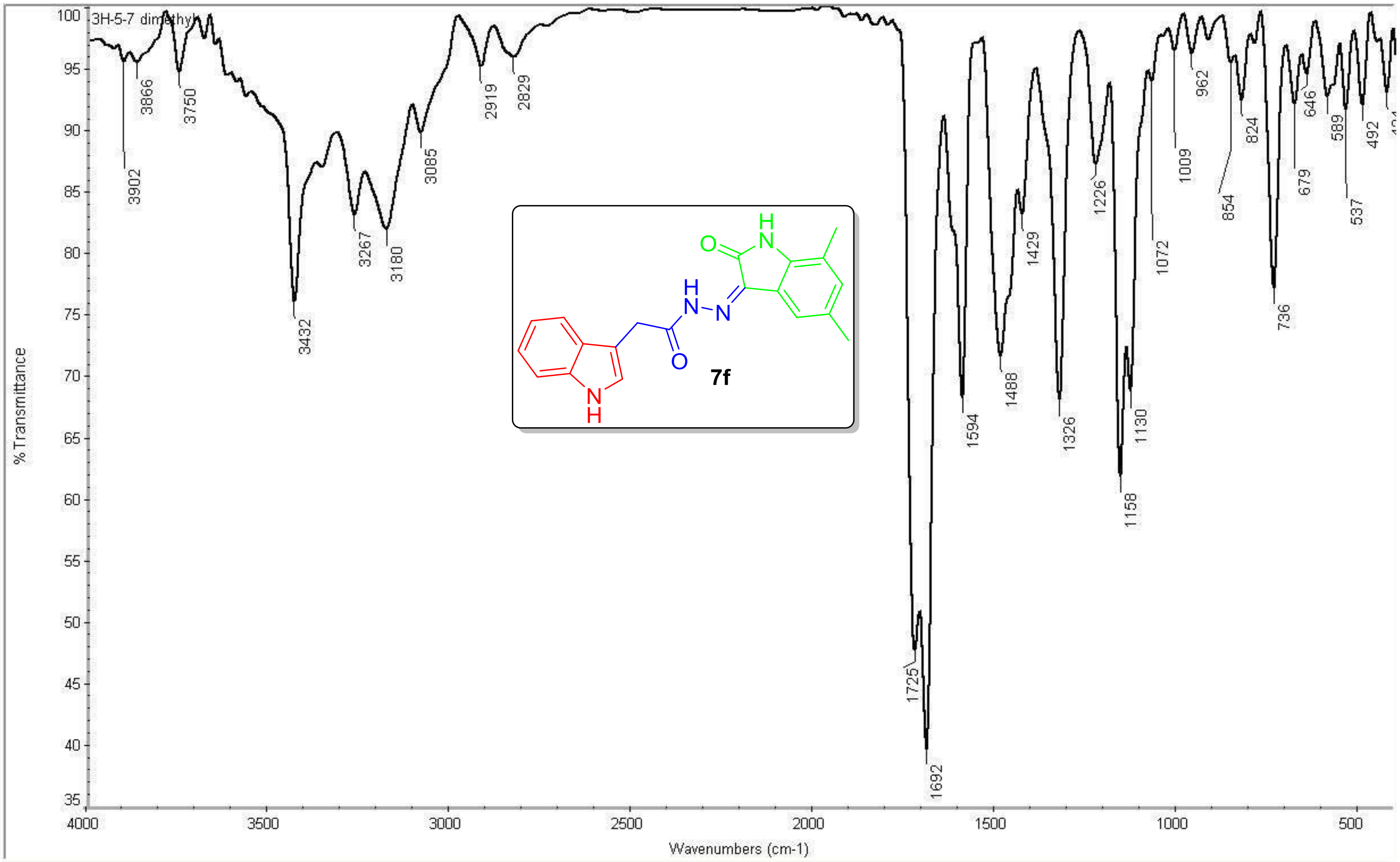

3H-iso bidyl

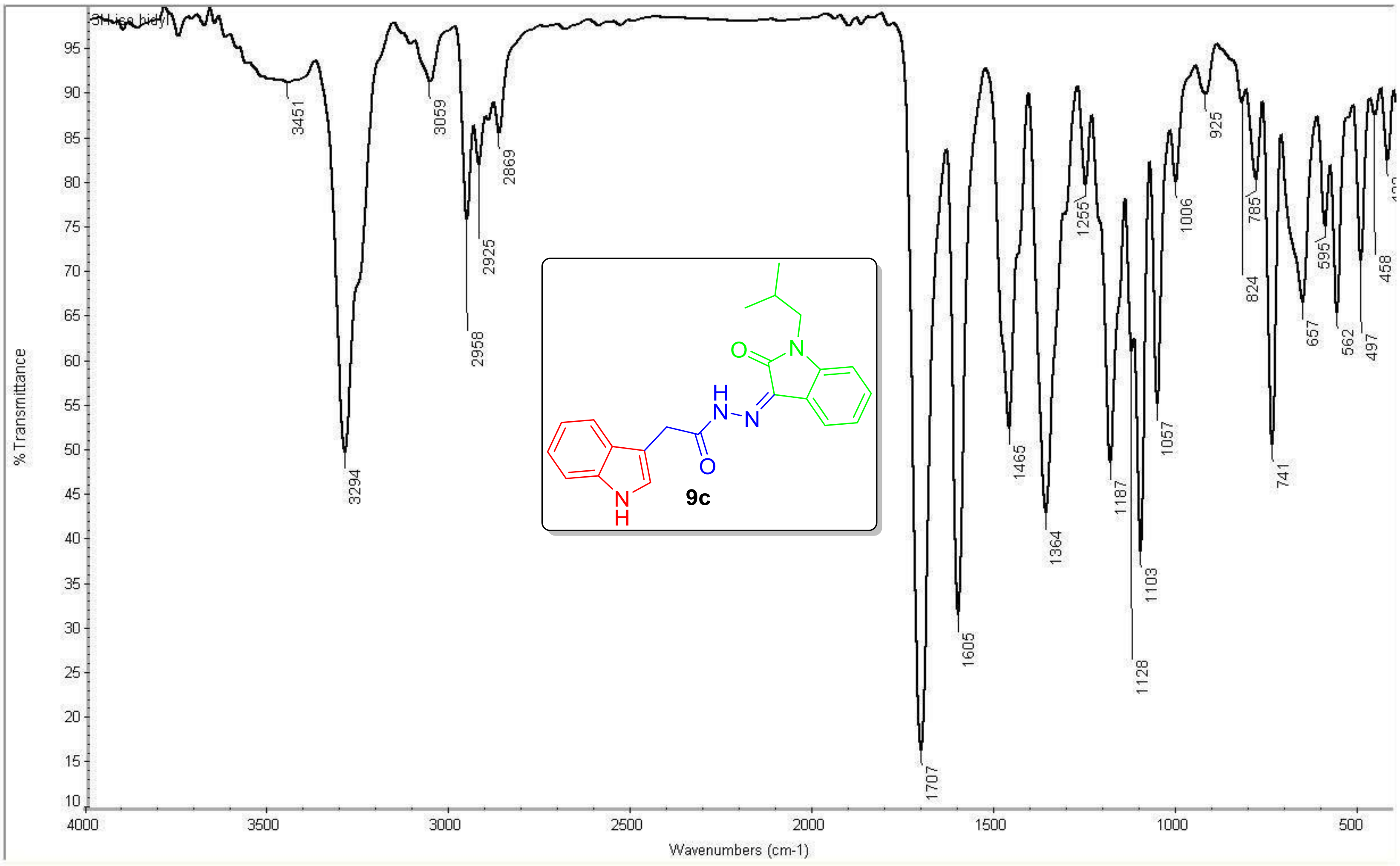
3H-iso bidyl
% Transmittance
Wavenumbers (cm-1)
95
90
85
80
75
70
65
60
55
50
45
40
35
30
25
20
15
10
4000
3500
3000
2500
2000
1500
1000
500
3451
3294
3059
2958
2925
2869
1707
1605
1465
1364
1255
1187
1128
1103
1057
1006
925
824
785
741
657
595
562
497
458
9c

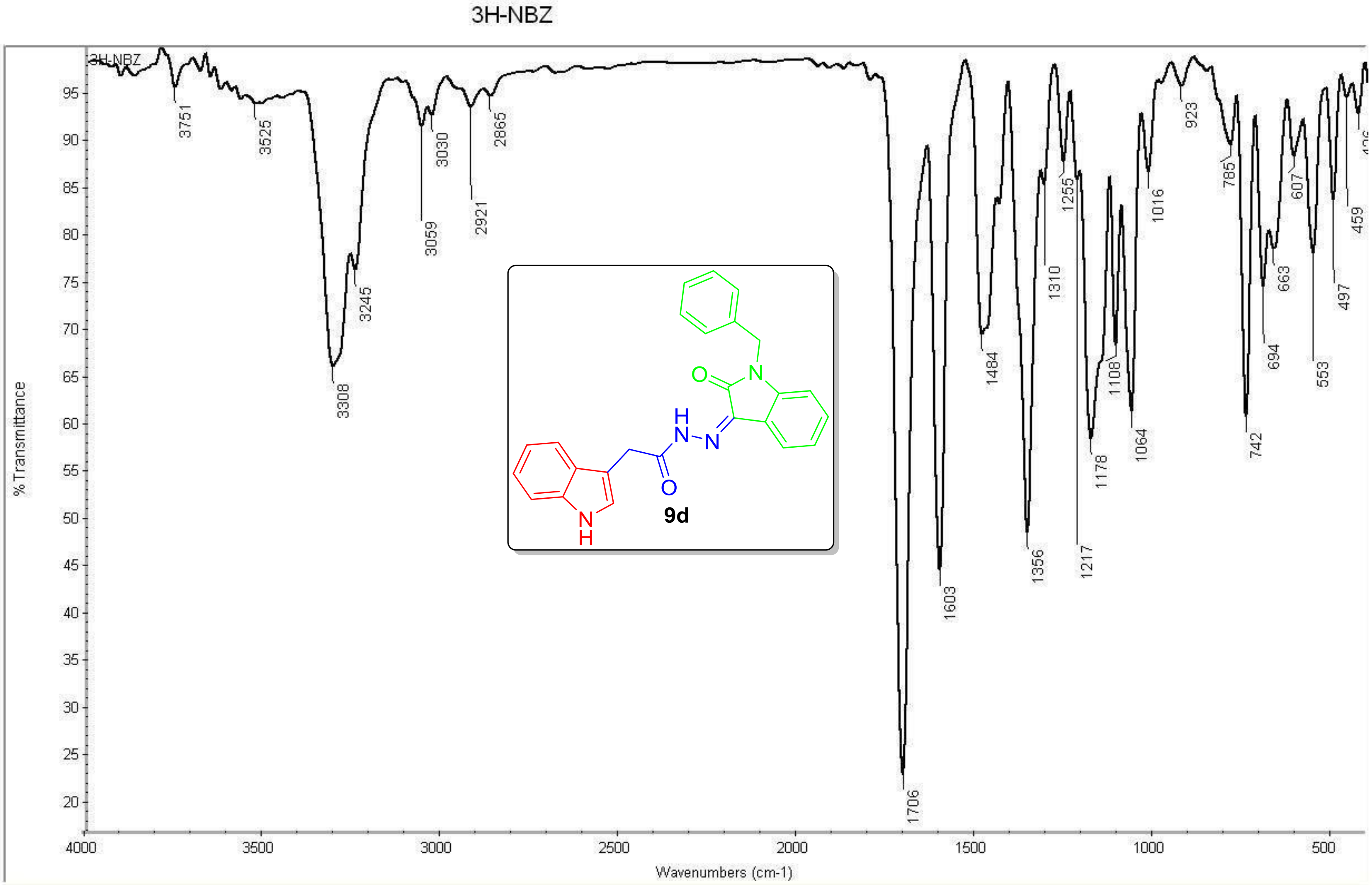
3H-NBZ
3H-NBZ
% Transmittance
Wavenumbers (cm-1)
3751
3525
3308
3245
3059
3030
2921
2865
1706
1603
1484
1356
1310
1255
1217
1178
1108
1064
1016
923
785
742
694
663
607
553
497
459
9d

3H-NPCN

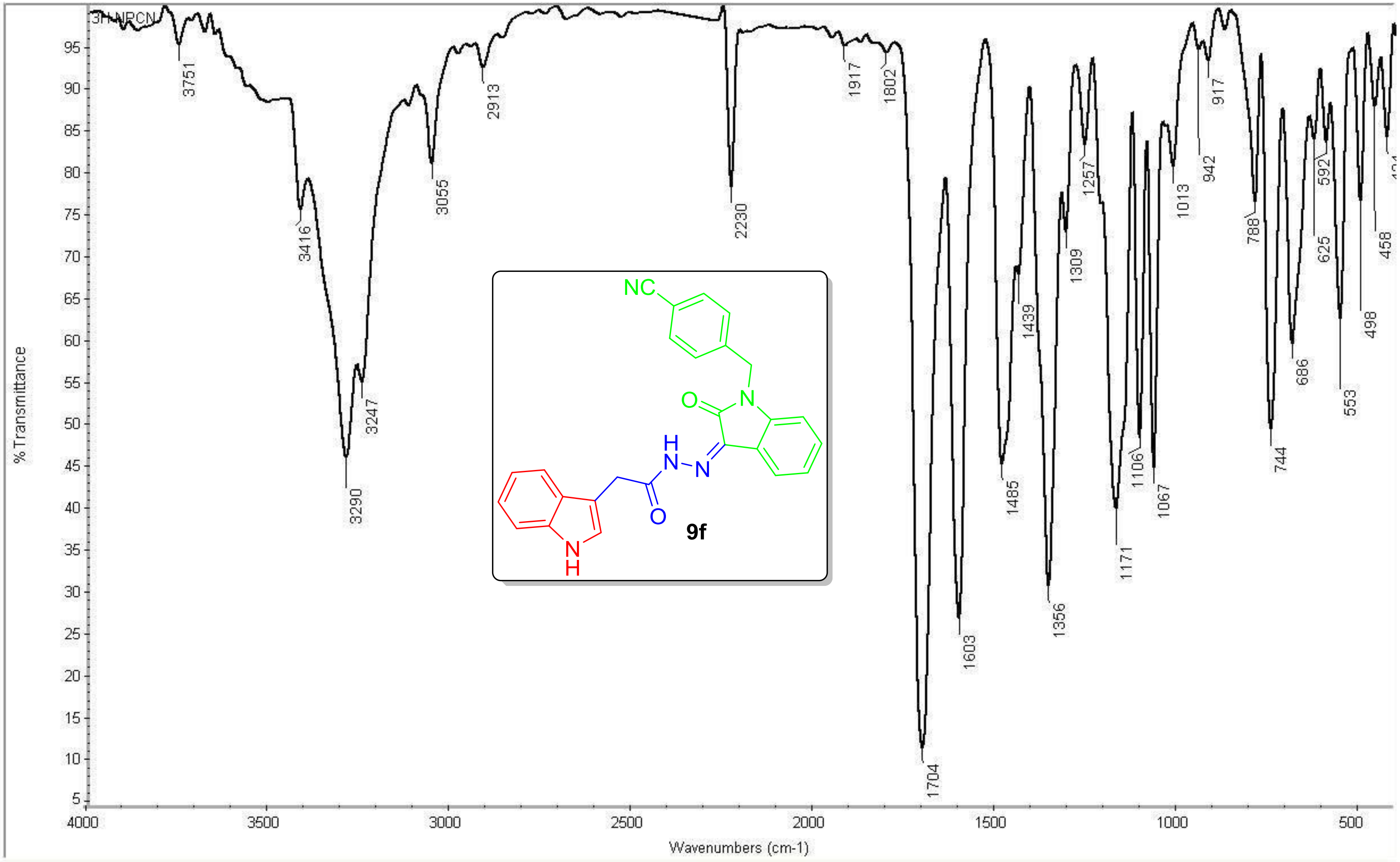
3H-NPCN
% Transmittance
Wavenumbers (cm-1)
4000
3500
3000
2500
2000
1500
1000
500
95
90
85
80
75
70
65
60
55
50
45
40
35
30
25
20
15
10
5
3751
3416
3290
3247
3055
2913
2230
1917
1802
1704
1603
1485
1439
1356
1309
1257
1171
1106
1067
1013
942
917
788
744
686
625
592
553
498
458
NC
O
N
H
N
N
O
N
H
9f

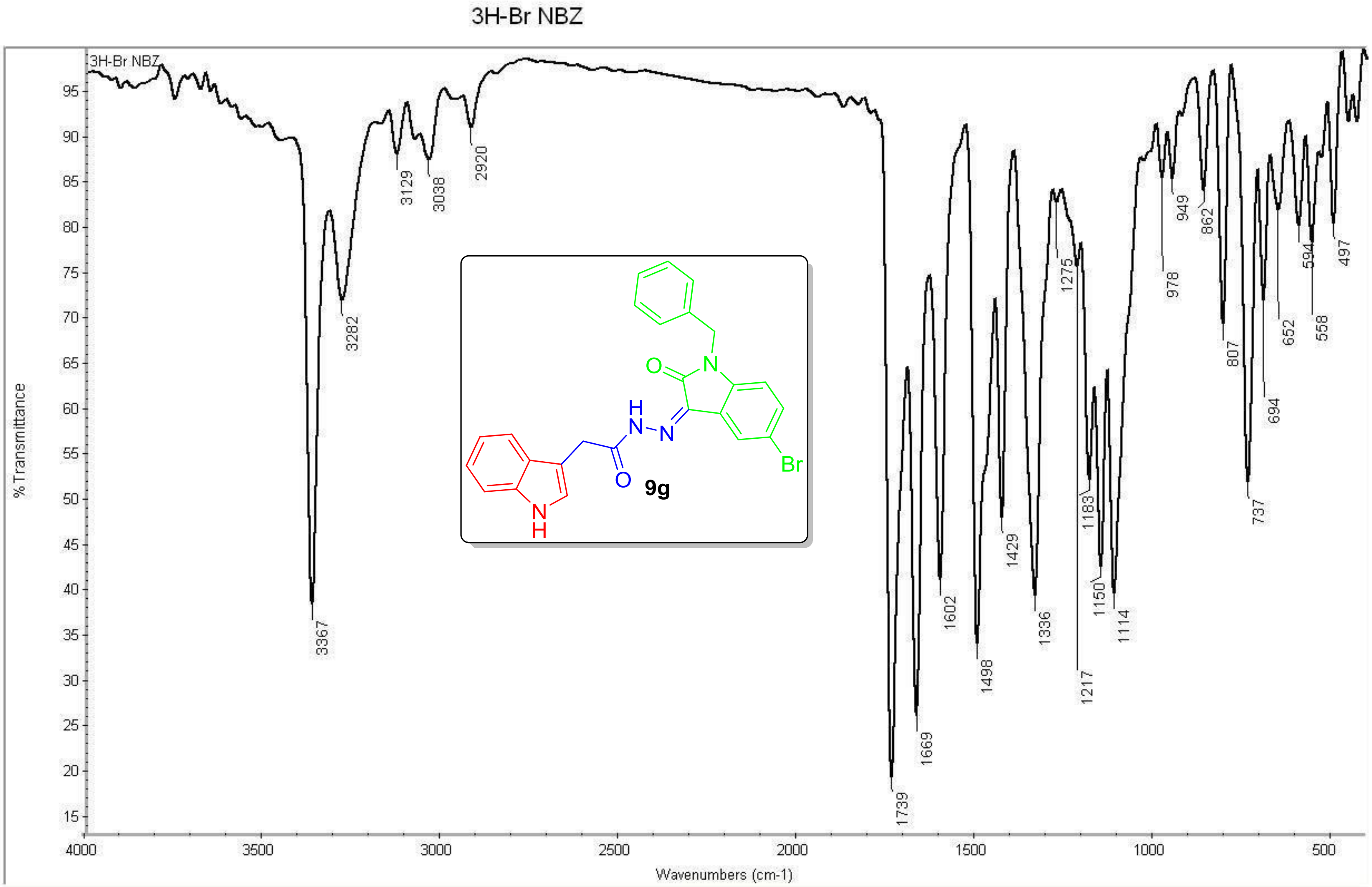
3H-Br NBZ
3H-Br NBZ
% Transmittance
Wavenumbers (cm-1)
3367
3282
3129
3038
2920
1739
1669
1602
1498
1429
1336
1275
1217
1183
1150
1114
978
949
862
807
737
694
652
594
558
497
9g
Br
N
O
H
N
N
O
N
H
